# Structure and Evolution of *Acinetobacter baumannii* Plasmids

**DOI:** 10.3389/fmicb.2020.01283

**Published:** 2020-06-18

**Authors:** Abraham D. Salgado-Camargo, Semiramis Castro-Jaimes, Rosa-Maria Gutierrez-Rios, Luis F. Lozano, Luis Altamirano-Pacheco, Jesús Silva-Sanchez, Ángeles Pérez-Oseguera, Patricia Volkow, Santiago Castillo-Ramírez, Miguel A. Cevallos

**Affiliations:** ^1^Programa de Genómica Evolutiva, Centro de Ciencias Genómicas, Universidad Nacional Autónoma de México, Cuernavaca, Mexico; ^2^Departamento de Microbiología Molecular, Instituto de Biotecnología, Universidad Nacional Autónoma de México, Cuernavaca, Mexico; ^3^Grupo de Resistencia Bacteriana, Centro de Investigaciones Sobre Enfermedades Infecciosas, Instituto Nacional de Salud Pública, Cuernavaca, Mexico; ^4^Departamento de Infectología, Instituto Nacional de Cancerología, Mexico City, Mexico

**Keywords:** *A. baumannii*, plasmids, Rep proteins, antibiotic resistance genes, plasmid maintenance functions, IS

## Abstract

*Acinetobacter baumannii* is an emergent bacterial pathogen that provokes many types of infections in hospitals around the world. The genome of this organism consists of a chromosome and plasmids. These plasmids vary over a wide size range and many of them have been linked to the acquisition of antibiotic-resistance genes. Our bioinformatic analyses indicate that *A. baumannii* plasmids belong to a small number of plasmid lineages. The general structure of these lineages seems to be very stable and consists not only of genes involved in plasmid maintenance functions but of gene sets encoding poorly characterized proteins, not obviously linked to survival in the hospital setting, and opening the possibility that they improve the parasitic properties of plasmids. An analysis of genes involved in replication, suggests that members of the same plasmid lineage are part of the same plasmid incompatibility group. The same analysis showed the necessity of classifying the Rep proteins in ten new groups, under the scheme proposed by Bertini et al. (2010). Also, we show that some plasmid lineages have the potential capacity to replicate in many bacterial genera including those embracing human pathogen species, while others seem to replicate only within the limits of the *Acinetobacter* genus. Moreover, some plasmid lineages are widely distributed along the *A. baumannii* phylogenetic tree. Despite this, a number of them lack genes involved in conjugation or mobilization functions. Interestingly, only 34.6% of the plasmids analyzed here possess antibiotic resistance genes and most of them belong to fourteen plasmid lineages of the twenty one described here. Gene flux between plasmid lineages appears primarily limited to transposable elements, which sometimes carry antibiotic resistance genes. In most plasmid lineages transposable elements and antibiotic resistance genes are secondary acquisitions. Finally, broad host-range plasmids appear to have played a crucial role.

## Introduction

*Acinetobacter baumannii* is a global emergent nosocomial pathogen that causes a wide variety of infections, especially in severely ill patients, in intensive care units. This pathogen is a major cause of morbidity and mortality in hospitals worldwide, and the recent success of this species as a pathogen seems to be linked to the ability of this organism to acquire antibiotic resistance genes, form biofilms and resist desiccation; these characteristics facilitate the persistence of this bacterium in the hospital setting and promote the emergence of outbreaks (Antunes et al., 2014). A large fraction of the nosocomial outbreaks in Europe, Asia, and North America are produced by a limited number of strains belonging to three different international clones (IC-I, IC-II, and IC-III) (Zarrilli et al., 2013). Most of these international clones are resistant to antibiotics belonging to three or more different families, a characteristic that defines these clones as being multidrug resistant (MDR) (Diancourt et al., 2010; Roca et al., 2012).

Plasmids are extrachromosomal DNA molecules, usually circular, that replicate independently of the chromosome and have the potential to be transferred frequently, but not exclusively by conjugation, not only to members of the same species but also to distantly related bacteria (Partridge et al., 2018). Plasmids play a leading role in the spread of antibiotic resistance genes among bacterial pathogens that cause community- or hospital-acquired infections, including *A. baumannii* (Carattoli, 2013; San Millan, 2018). A wide variety of *A. baumannii* plasmids carrying antibiotic resistance genes with different sizes and characteristics have been described in recent literature. There has been particular interest in plasmids carrying genes encoding serine carbapenemases (OXA-type beta-lactamases), which facilitate the most predominant mechanism for carbapenem resistance in this species (Higgins et al., 2010; Mosqueda et al., 2014; Hujer et al., 2017; Cameranesi et al., 2018; Wibberg et al., 2018).

Despite the apparent importance of plasmids in the spread of virulence and antibiotic resistance genes among *A. baumannii* isolates, only a few papers that have analyzed the structures, relationships and evolution of *A. baumannii* plasmids as a whole have been published (Fondi et al., 2010; Lean and Yeo, 2017; Salto et al., 2018). In this work, taking advantage of the increasing interest in *A. baumannii* and the large number of complete genome sequences for this organism that have been deposited in GenBank in the last decade, we performed a comparative plasmid sequence analysis to gain insights into the structures, relatedness, and evolution of these plasmids. We were able to determine that the *A. baumannii* plasmids belong to a small number of plasmid lineages, some of them widely distributed among the different *A. baumannii* clades, while others seem to be restricted to a small number of clades. Surprisingly, some widespread plasmids do not have genes linked to conjugation or plasmid mobilization, suggesting that other mechanisms or horizontal transfer play an important role in the dissemination of *A. baumannii* plasmids. Genes encoding initiator replication proteins and the corresponding surrounding DNA sequences within each plasmid lineage are similar enough to suggest that each lineage represents plasmids of the same incompatibility group. This suggestion is also supported by the observation that plasmids of the same strain have different replication proteins. Each plasmid lineage possesses a common gene set that contains not only genes involved in plasmid maintenance but also a set of genes encoding hypothetical or poorly characterized proteins. Despite the antibiotic or metal resistance genes that some plasmids possess, the remaining genes that are not involved in plasmid maintenance are not obviously linked with properties that allow survival in the hospital setting, suggesting that these genes could be associated with plasmid survival functions. Additionally, we determined that gene transfer from one plasmid lineage to another is highly limited and restricted to a few gene classes.

## Results and Discussion

### The Plasmid Collection

Next-generation sequencing platforms have been a crucial means to obtain the complete sequences of all types of bacterial genomes, including those of many important human pathogens. We took advantage of the large amount of information generated in this manner to analyze the structure and evolution of the *A. baumannii* plasmids. For this purpose, we used the 155 complete plasmid sequences deposited in NCBI as of August 14, 2017. However, considering that most of these plasmid sequences were obtained from isolates of international clones and/or from a restricted set of countries, we incorporated the sequences of 18 plasmids obtained from the genome sequences of 10 nosocomial strains that represent some of the most prevalent STs circulating in Mexico to increase the plasmid diversity included in our investigation (see Materials and Methods). In total, our study collection comprised 173 plasmids of a wide variety of sizes, ranging from 1,109 to 216,780 bp. Moreover, our plasmid set originated from 103 different isolates, each carrying up to six plasmids. These isolates belonged to at least 47 different STs and originated from 17 countries (see Supplementary Table S1).

### *A. baumannii* Plasmids Belong to a Very Restricted Number of Plasmid Lineages

Plasmids have been visualized as molecules that possess genes involved in self-maintenance (plasmid backbone) and genes that could be important for the ability of bacteria to exploit new ecological niches or acquire new capabilities (Frost et al., 2005). These genes are commonly described as plasmid *cargo*. Antibiotic resistance genes are a perfect example of such genes, particularly for organisms in hospital settings (Tschäpe, 1994; Carattoli, 2013; San Millan, 2018).

To understand how plasmids are organized and to define which are the relationship between them, several plasmid classification systems have been proposed. Some of these systems relay in the phenotypic features that plasmids confer, assuming that plasmids sharing such characteristics are phylogenetically related. Plasmid incompatibility or the inability of two plasmids to reside in the same cell has been another way to classify plasmids. Plasmids belonging to the same incompatibility group have identical or very similar replication and/or segregation gene modules (Novick, 1987; Austin and Nordström, 1990). With this idea in mind, some authors have developed typing systems based on the nucleotide sequence identity of the genes encoding replication initiation proteins. Other authors designed methods to classify conjugative plasmids based on the sequence of the relaxase, a gene crucial for conjugation. The problem with these classification systems is that they are based on a limited number of genes or traits. However, considering the diversity of genes carried on plasmids and the different mechanisms that plasmid use for their maintenance makes a futile dream to design a universal plasmid taxonomy system. Nevertheless, we can design a classification system that takes into account, in an unbiased way, the whole gene content of plasmids, to determine which are the relationships between them and to have a picture of how these plasmids evolve. This was the approach that we follow in this work.

Plasmid evolution can be thought to occur via two basic pathways: first, plasmids are entities that are prone to rapid loss and gain of genes such that, in a short period of time, descendants of one plasmid are only recognizable because they share the same set of genes involved in the basic maintenance functions of the plasmid (Hülter et al., 2017; Brandt et al., 2019). Second, the ability of plasmids to gain or lose genetic information can be assumed to be more or less limited, and the plasmids persist for long durations within bacterial populations as plasmid lineages, where plasmid lineages are groups of plasmids that are closely related by gene content, including, but not restricted to, genes responsible for plasmid maintenance (Yau et al., 2010).

Our first interest was to evaluate, precisely, the type of evolution undergone by *A. baumannii* plasmids. For this purpose, our strategy was to compare the degree and extent of DNA sequence identity between the plasmids in our collection. We used nucleotide MEGABLAST (BLASTn) searches instead of Protein BLAST (BLASTp), as described by other authors, for two reasons: first, BLASTn comparisons are less sensitive to sequencing errors introduced during the assembly process (false frame shifts or incorrect stop codons) than BLASTp, and second, a BLASTp approach does not take into consideration intergenic regions and regions essential for plasmid function, such as the origin of replication. We made pairwise MEGABLAST (BLASTn) comparisons of each plasmid of our collection against the others. To filter BLAST results, we constructed networks with the following rule: two plasmids are linked if at least 85% of the regions of the largest plasmid (for each comparison) are covered by the smaller plasmid, and those regions exhibit at least 90% of DNA sequence identity. To belong to a specific network, one plasmid must fulfill the above-mentioned cutoff values of identity and coverage not with all, but with at least one member of the group. Being a member of a specific network does no mean that all plasmids of this network have at least 85% coverage with the rest of the members. The minimal requirement is to accomplish the cutoff values with at least one member of the network, for example, the shortest with the next in size.

After these analyses, we determined that 124 *A. baumannii* plasmids were organized into 23 groups, and 39 plasmids remained without an assigned group. The plasmid composition of each group is listed in Supplementary Table S1. As shown in Supplementary Figure S1, the plasmid networks constructed as mentioned above are densely interconnected, and all members of a determined group have the same or a very closely related gene encoding a DNA replication initiator (Rep) protein. Notably, plasmids within a group share, in general, several genes that are involved in plasmid maintenance.

To evaluate the coherence of these groups, we repeated the analysis, raising plasmid coverage to 90% again, with 90% DNA sequence identity. In general, the groups remained almost the same (some groups lost a few members). On the one hand, lowering coverage to 50% and retaining 90% of DNA sequence identity, allows the incorporation of some orphans into different groups and led to the fusion of six lineages: Group_17 with Group_22, Group_7 with Group_8 and Group_3 with Group_14. Members of lineages Group_17 share sequence identity of approximately 70% with components of Group_22, including the replication module and nearby sequences. This grouping suggest that Group_17 and Group_22 have a common evolutionary origin. Likewise, members of Group_3_and Group_14 have similar but not identical Rep proteins, indicating also that they have a hypothetical common ancestor. In contrast, members of Group_8 do not have the same replication module as those belonging to Group_7 and for this reason we do not contemplate them having a common ancestry.

Groups formed using 85% coverage and 90% of sequence identity as cutoff values represent a useful method for identification of *A. baumannii* plasmid lineages. Lowering the coverage cutoff value to 50% may be useful to recognize ancestral relationships, as long as the shared sequences include the replication/maintenance module. Therefore, hereinafter, we will consider each one of the groups identified with this methodology as a plasmid lineage.

However, to indicate that Group_3_and Group_14 had a common origin but now each one of the groups has a different evolutionary path, these were named as plasmid lineages LN_3A and LN_3B, respectively. With these considerations, members of our plasmid collection belong to 21 plasmid lineages and 39 plasmids remain as orphans (not assigned to a plasmid lineage). Interestingly, 88 plasmids, or 50.8% of our collection, were clustered in only four plasmid lineages: LN-1, LN_2; LN_3 and LN_4. The other 17 groups are very small, as most of them contained only two members (Supplementary Figure S1).

With only one exception, we elected the largest and most interconnected member of the group as the representative plasmid of each lineage. The exception is lineage 2 (LN_2), in which the largest and most interconnected member has a very large duplicated region. The duplicated regions include the replication genes indicating that this sequence has assembly problems, considering that plasmids with duplicated replication regions are highly unstable and they are rapidly eliminated of the population (Summers et al., 1993). Therefore, the second largest most interconnected plasmid (pPKAB07) was selected as the representative of this particular lineage. In conjunction, these analyses indicate that *A. baumannii* plasmids evolve as lineages and that most of the *A. baumannii* plasmids in circulation worldwide belong to a few lineages.

The general structure of the members of each one of the plasmid lineages is very stable, considering that some of the strains were isolated many years ago. For example, strain A1 was isolated in 1982, and one of the plasmids of this strain, pA1-1, belongs to lineage LN_2. This plasmid has a very similar gene content and organization as other plasmids isolated in 2015 that belong to the same lineage (plasmid unnamed2, GenBank accession number CP014293). Similarly, plasmid pALAC4-2 of LN_4 belongs to a strain isolated in 1997 and has a very similar structure to other plasmids of the same lineage isolated a decade later (i.e., plasmid pMRSN3527-6, GenBank accession number NZ_CM003318.1). Additionally, plasmid p4ABAYE (GenBank accession number NC_010403.1), described in 2001, shared 98% sequence identity with pMRSN58-2.7 (GenBank accession number NZ_CM003316.1), isolated in 2013. Members of LN_19 are almost identical. The oldest member of the lineage was isolated in 2001 and the most recent in 2010 (Supplementary Table S1).

The *A. baumannii* plasmid sequences deposited in NCBI have increased since we last performed the analyses. On April 28, 2020, this database embraced the complete sequence of 422 A baumannii plasmids. To make a rapid evaluation of the prevalence of plasmid lineages LN_1, LN_2, LN_3A, LN_3B, and LN_4, we performed BLASTn on all members of these lineages against the new database. Using this strategy 30 new plasmids were incorporated in LN_1, 23 in LN_2, 12 in LN_3A+3B, and finally, 10 new plasmids were included in LN_4. Now, these lineages contain 38.4% of the *A baumannii* plasmids. However, we must say that the only way to identify all new members of the plasmid lineages is by reconstructing the networks with the rules mentioned above. These observations confirm that a few plasmid lineages encompass most of *A. baumannii* plasmids.

### Plasmid Lineage Gene Composition

Comparisons of all members of a particular plasmid lineage with their representative plasmid show that the genome core of a plasmid lineage includes genes that are not involved in plasmid maintenance functions (the backbone) (Figure 1 and Supplementary Figures S2–S12).

**FIGURE 1:**
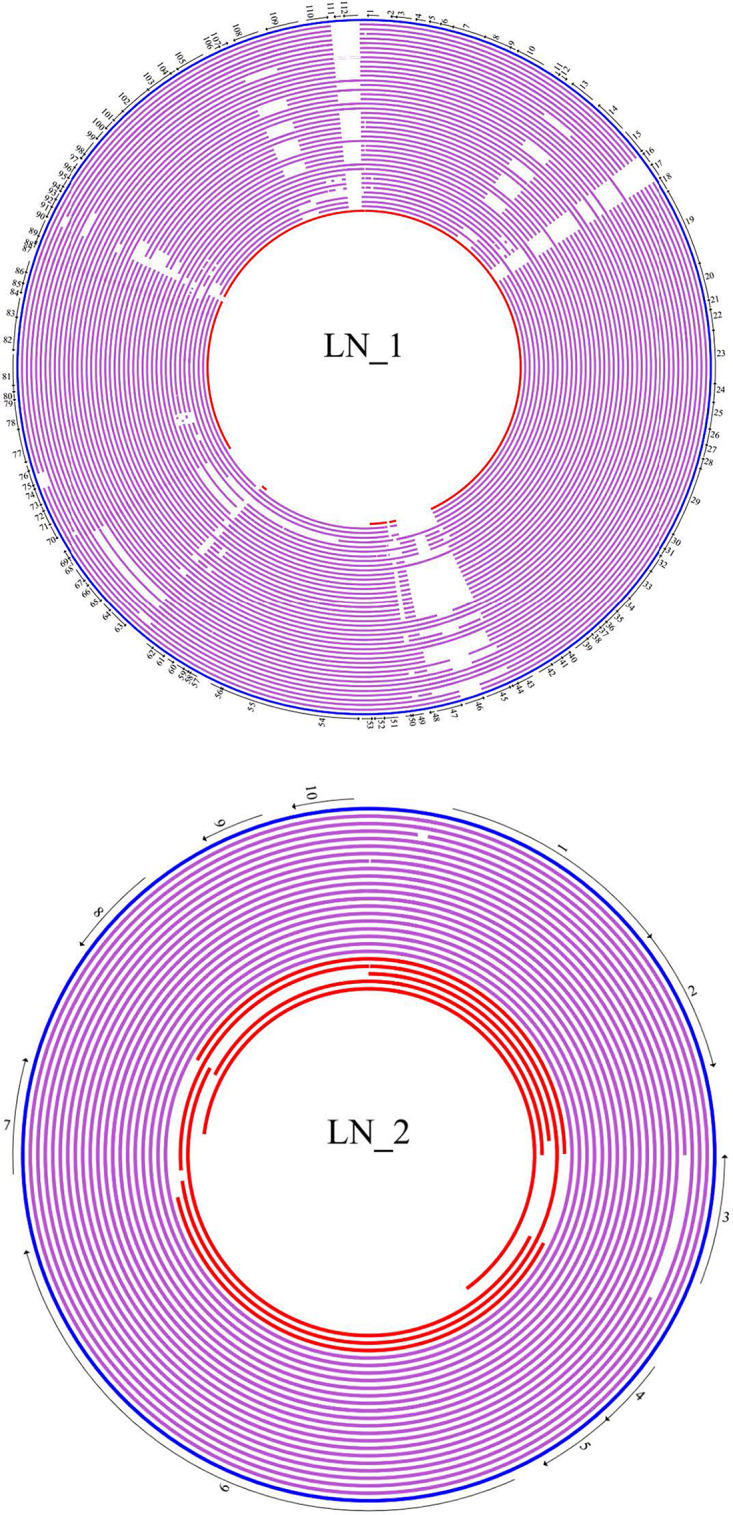
Cicular map of members of plasmid lineage LN_1 and linear map of members of LN_2. Plasmid sequences of each lineage were compared with its reference with BLASTn and mapped using GenVision a component of DNASTAR’s Lasergene Core Suite. The genes core are in bold letters. The representative plasmid of LN_1 is plasmid AB34299 (blue ring) and numbers around this plasmid indicate the position of the following genes: **1. Nuclease; 2. Hypothetical protein; 3. Hypothetical protein; 4. Hypothetical protein; 5. Hypothetical protein; 6. Hypothetical protein; 7. Zeta-antitoxin; 8. Zeta-Toxin; 9. Hypothetical protein; 10. Plasmid replicase; 11. Hypothetical protein**; 12. Hypothetical protein; 13. Hypothetical protein; 14. DNA polymerase; 15. Hypothetical protein; 16. ISAba1; 17. ISAba1; **18. Transglycosylase; 19. Conjugal protein TraG; 20. Conjugal protein TraH; 21. Hypothetical protein; 22. Conjugal protein TraF; 23. Conjugal protein TraN; 24. Conjugal protein TrbC; 25. Conjugal protein TraU; 26. Conjugal protein TraW; 27. Peptidase; 28. Hypothetical protein; 29. Conjugal protein TraC; 30. Conjugal protein Tra; 31. Hypothetical protein; 32. Protein-disulfide isomerase; 33. Conjugal protein TraB; 34. Conjugal protein TraK; 35. Conjugal protein TraE; 36. Conjugal protein TraL; 37. Hypothetical protein; 38. Hypothetical protein; 39. Murein transglycosylase; 40. Hypothetical protein; 41. Hypothetical protein; 42. Resolvase; 43. Hypothetical protein**; 44. Hypothetical protein; 45. ISAba125; 46. Aminoglycoside phosphotransferase; 47. ISAba125; 48. Hypothetical protein; 49. Hypothetical protein; 50. Hypothetical protein; **51. Hypothetical protein**; 52. Hypothetical protein; 53. Hypothetical protein; 54. Relaxase MOBF; 55. Type IV secretion system protein VirD4; 56. Hypothetical protein; 57. Hypothetical protein; 58. Hypothetical protein; 59. Hypothetical protein; 60. Hypothetical protein; 61. Molecular chaperone DnaJ; 62. Hypothetical protein; 63. Hypothetical protein; 64. Hypothetical protein; 65. Hypothetical protein; 66. Hypothetical protein; 67. Hypothetical protein; 68. Addiction module toxin; 69. DNA-binding protein; **70. Hypothetical protein; 71. Hypothetical protein;** 72. Hypothetical protein; 73. Hypothetical protein; **74. Hypothetical protein**; 75. Hypothetical protein; 76. Hypothetical protein; 77. Hypothetical protein; **78. Toxic anion resistance protein TelA; 79. Hypothetical protein; 80. Hypothetical protein; 81. Hypothetical protein; 82. ParB family partition protein; 83. ParA family protein; 84. Hypothetical protein; 85. Hypothetical protein; 86. Hypothetical protein; 87. Hypothetical protein; 88. Hypothetical protein**; 89. Hypothetical protein; 90. Hypothetical; protein; 91. Hypothetical protein; 92. Hypothetical protein; 93. Hypothetical protein; 94. DNA-binding protein; 95. Nuclease; **96. Hypothetical protein; 97. Hypothetical protein; 98. Hypothetical protein; 99. Hypothetical protein; 100. Hypothetical protein; 101. Hypothetical protein; 102. Zeta-antitoxin; 103. Zeta-toxin; 104. Hypothetical protein; 105. Hypothetical protein; 106. Hypothetical protein; 107. Hypothetical protein**; 108. Hypothetical protein; **109. DNA polymerase**; 110. Hypothetical protein; 111. Transposase; 112. Transposase. Purple rings: Purple rings: from outside to inside: pNaval18-74, p2ABTCDC0715, pAC30c, pAba10042b,pAba9102a, pAba7847b, pACICU2, ABKp1, p1ABST2, pNaval81-67, pOIFC143-70, pIS123-67, pABUH1-74, p1AB5075, pAB04-2, plasmid YU-R612, pAba3207b, pCMCVTAb2-Ab4, plasmid CMC-CR-MDR-Ab66, plasmid KAB01, plasmid KAB02, plasmidKAB03, plasmid KAB04, plasmid KAB05, plasmid KAB06, pSSA12_1, pSSMA17_1,pJBA13_1, p15A34_1, pUSA2_1, pUSA15_1, pA85-3, pCS01A, pCS01B, pCR17A,pCR17B, pAba7835b, plasmid KAB07, plasmid KAB08, p15A5_1. Additional orphan plasmid is in red ring: pAC29b. LN_2. The representative plasmid of lineage LN_2 is pPKAB07 (blue bar). Numbers along this bar indicate the position of genes: **1. RepB family plasmid replication initiator protein; 2. DNA-binding protein**; 3. Hypothetical protein; 4. Toxin-Antitoxin system spITA (COG3514); 5 Toxin-Antitoxin system spITA (DUF497); 6. TonB dependent receptor; 7. Hypothetical protein; 8. Hypothetical protein; 9. Hypothetical protein; 10. Hypothetical protein. Purple bars: from top to bottom: p1ABTCDC0715, pAC12, pAC30a, p2ABAYE, pAB0057, p1BJAB0868, pCanadaBC5-8.7, pABUH6a-8.8, pMRSN7339-8.7, pMRSN58-8.7, pAB0057, plasmid_2 AB34299, p2AB5075, pAC29a, pA1-1, p15A5_2, pSSA12_2, pA85-2, pAB5075. Additional orphan plasmids are in red bars: from top to bottom: pAB2, p1ABST78, pORAB01-3, p MEX11594, pYU-R612.

**FIGURE 2:**
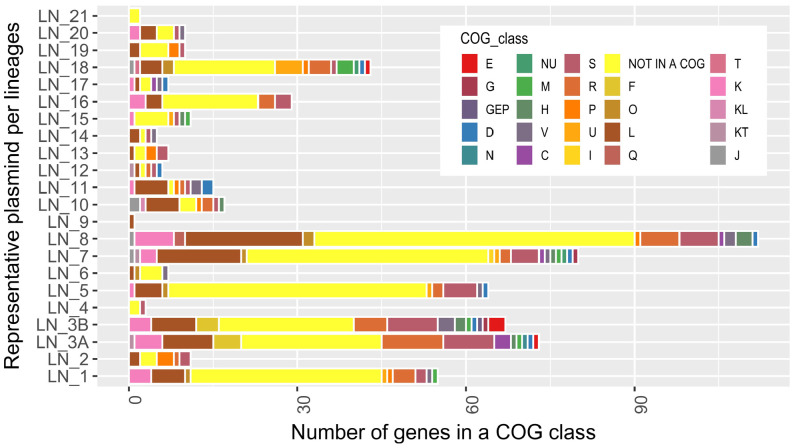
Number of genes assigned to a functional class (COG) present in the representative plasmid of each lineage. Classes: CO, energy production and conversion, posttranslational modification, protein turnover, chaperones. DJ, cell cycle control, cell division, chromosome partitioning, translation, ribosomal structure and biogenesis. Q, secondary metabolites biosynthesis, transport and catabolism I, Lipid transport and metabolism. GEP, carbohydrate transport and metabolism, amino acid transport and metabolism, inorganic ion transport and metabolism. KT, transcription, signal transduction mechanisms. NU, cell motility, intracellular trafficking, secretion, and vesicular transport. G, carbohydrate transport and metabolism. KL, transcription, replication, recombination and repair. J, translation, ribosomal structure and biogenesis. T, signal transduction mechanisms. E, amino acid transport and metabolism. D, cell cycle control, cell division, chromosome partitioning. H, coenzyme transport and metabolism. F, nucleotide transport and metabolism. C, energy production and conversion. M, cell wall/membrane/envelope biogenesis. U, intracellular trafficking, secretion, and vesicular transport. O, posttranslational modification, protein turnover, chaperones. P, inorganic ion transport and metabolism. V, defense mechanisms. K, transcription. S, function unknown. R, general function prediction only. L, Replication, recombination and repair. NOT IN A COG, COG not defined.

To obtain a general picture of the gene composition of our plasmid collection, we assigned a functional class (COG) to each of the protein products encoded by these plasmids. This analysis showed that these proteins fall within 23 functional classes; however, we were unable to assign a functional class (not in COG) to 74.15% of the proteins (Figure 2). In total, 3.53% of the encoded proteins have only a general function prediction (class R), and 3.5% are classified within class S (function unknown). Nevertheless, these 2497 uncharacterized or poorly characterized proteins were grouped in 242 orthologous groups [Remained Orthologous Groups (ROGs)] (Taboada et al., 2010). Therefore, it is not possible to predict whether some of these hypothetical proteins play a role in the nosocomial setting. However, given that the genes encoding these proteins are highly conserved within each plasmid lineage; the general structure of plasmids belonging to each one of the different lineages is stable during time and that the plasmids replicate in very different genetic backgrounds (even in different species), we suggest that these genes may play a role in reducing the fitness cost for the host to maintain the plasmids, thereby improving the favorability of the plasmids as parasite molecules.

We also found, as expected, a set of genes encoding proteins that are typically associated with plasmids: 7.28% of the proteins fall under class L (replication, recombination and repair), which includes replication initiation proteins, transposases, site-specific recombinases, and other proteins involved in recombination. Additionally, 1.31% of the proteins belong to class V (defense mechanisms), which includes proteins involved in plasmid stability (toxin-antitoxin modules) and restriction modification and proteins conferring antibiotic resistance. In the following sections, we will describe genes that play a crucial role in plasmid maintenance and that are usually associated with plasmid functions (Figure 2).

### Classification of New Replication Initiation Protein (Rep) Genes

An absolute requirement for the survival of a plasmid is the presence of a replication module. These modules consist of an origin of replication, one gene encoding a replication initiator (Rep gene) and the factors and DNA sites involved in regulation of the expression of this gene, which is located near the Rep gene (del Solar et al., 1998). Our bioinformatic analysis indicates that from the 173 plasmids in our collection, 143 had an intact Rep gene and 13 plasmids had Rep pseudogenes, because we found on them premature stop codons or frameshifts generated, probably, during the sequencing and/or assembly processes. Nevertheless, in 27 plasmids, we could not find a Rep protein by annotation or BLAST searches; thus, as already noted by other authors, an experimental approach is needed to identify such replication regions (Lean and Yeo, 2017).

**FIGURE 3:**
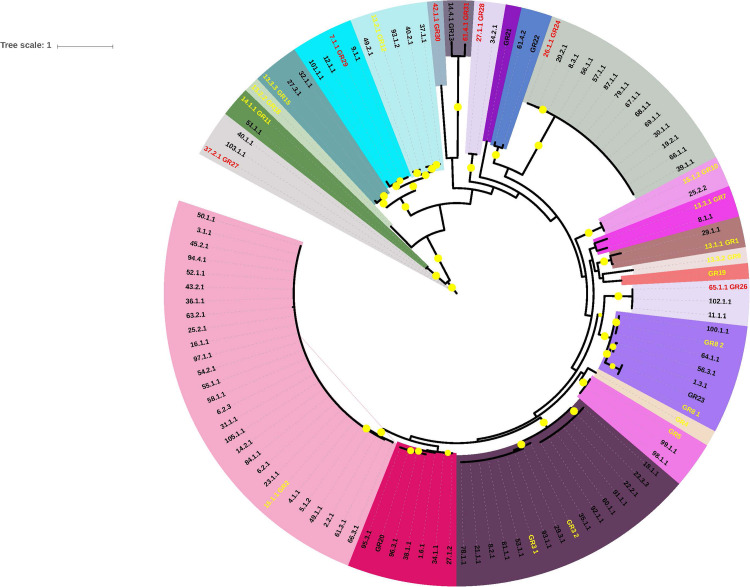
Phylogenetic tree of genes encoding replicase proteins belonging to the Rep_3 family. Gene codons were aligned guided by protein alignments. In this figure we are using the replicase_ID numbers listed in Supplementary Table S2. Each color embrace members of one clade. Names with yellow letters indicate the reference genes used by Bertini et al. (2010) to construct GR homology groups. Names with red letters show the reference genes used by us to construct the new GR homology groups. Bootstrap values higher than 70% are marked in the figure with yellow circles.

Rep genes of *A. baumannii* plasmids have been mainly identified by bioinformatic analyses, which have indicated that these proteins can be classified into five different categories: the most common Rep proteins belong to the Rep_3 superfamily (Pfam:01051) and are usually annotated as RepB. The next most frequent Rep proteins are those annotated as *replicases*, and these proteins have two distinctive domains: one is a replicase domain (pfam:03090), and the other is an alpha-helical domain that is also present at the C termini of primases (PriCT_1 superfamily). Other *A. baumannii* plasmids have Rep proteins belonging to the Rep_1 superfamily (Pfam01446). Several plasmids have a protein with a helix-turn-helix (HTH) domain annotated as a replication protein, and finally, one plasmid has an initiator protein classified as belonging to the RepC superfamily (Pfam:06504). Recently, the functionality of some of these replication regions was tested experimentally (Salto et al., 2018). Nevertheless, as mentioned above, many plasmids do not have an identifiable Rep protein. Figure 3 and Supplementary Figure S13 show the phylogenies of the replicases with the most members, separated by the domains identified by Pfam (Rep3 and replicase-PriCT). Some proteins were not included because either these proteins did not have any Pfam domain assigned in the database or there were not enough members to perform comparisons, as in the extreme case of the RepC domain, with only one protein assigned to this domain(El-Gebali et al., 2019).

In 2010, Bertini and coworkers designed a classification system for the *A. baumannii* plasmids based on the nucleotide identity of the Rep genes (Bertini et al., 2010). Rep genes that shared at least 74% nucleotide identity were pooled in the same group. With this scheme, the authors identified 19 homology groups (GR1 to GR19). Subsequently, Lean and Yeo, studying A. *baumannii* plasmids of less than 10 kb, proposed a new group based on Rep phylogenetic analyses: GR20, which is closely related to GR2; however, the members of this group form a clear separate clade (Lean and Yeo, 2017). Recently, Cameranesi and collaborators analyzed *A. baumannii* plasmids from Argentina and determined that some Rep genes of these plasmids required the formation of three additional groups: GR21, GR22 and GR23 (Cameranesi et al., 2018). However, the analysis of the genes annotated as Rep proteins from our plasmid collection showed that the current classification system was not sufficient to include all the Rep proteins. Therefore, by following the scheme proposed by Bertini and coworkers, ten additional groups were constructed (GR24-GR33). These replication gene groups can be visualized as a network in which one gene encoding a replication protein is part of a group if its DNA sequence shares at least 74% identity and 90% coverage with another member of the same group. For each new Rep group (GR), we chose the most interconnected member as the representative sequence of the group. However, we identified some unusual Rep proteins that showed nucleotide sequence identity higher than 74% with members of two different groups. This inconsistency was provoked because some authors named new replication homology groups, using a different set of rules of those originally proposed by Bertini and coworkers. Examples, the representative protein of GR23 is identical to that of GR8_1 proposed by Bertini and coworkers or the representative members of groups GR2 and GR20 have a DNA sequence identity higher than 74%. (Fondi et al., 2010; Cameranesi et al., 2018). In these cases, we assigned the unusual protein to the group with which this element shared the highest nucleotide identity (Supplementary Table S2). The assignations of all the replication proteins encoded in our plasmid set are listed in Supplementary Table S1. Seven of the new groups harbor DNA initiator proteins of the Rep_3 family (GR26, GR27, GR28, GR29, GR30, GR31, GR32); two of these groups are composed of proteins of the Replicase_PriCT family (GR25 and GR32), but the representative member of GR25 has an HTH_29 additional conserved domain (Pfam13551) and finally, one plasmid carries a Rep protein of the RepC family (GR33). We were incapable of identifying a gene encoding a Rep protein in three plasmid lineages (LN_4, LN_7 and LN_18) and in 14 orphan plasmids. On the other hand, four plasmid lineages, namely, LN_2, LN_11, LN_13 and LN_20, exhibited the same organization in their replication modules. This module consists of a bicistronic operon, in which the first gene encodes an initiator protein of the Rep_3 family (or RepB), and the second gene of the operon encodes a protein with an HTH motif that on some occasions has been wrongly annotated as a putative Rep protein, for example, in the homology group GR17. We traced the error source to an obvious mistake in GenBank: plasmid pAB1 (GenBank accession number CP000522.1) carries a gene annotated as encoding a DNA replication protein (protein_id ABO13850.1) which is precisely the representative member of GR17. This putative DNA replication protein carries an HTH_17 conserved domain (Pfam12728). However, a BLAST search indicates that the gene upstream to that encoding the HTH-carrying protein encodes a protein belonging to the Rep_3 superfamily (protein_id ABO13860.1) which is identical to other *A. baumannii* replication proteins. Unfortunately, this gene is annotated as encoding a hypothetical protein. To facilitate future work, Rep proteins sequences and the genes that codify them are listed in Supplementary Materials S1, S2. Be careful: these lists still include the representative member of GR17 described above.

### Iterons in the New GR Replication Homology Groups

It has been shown that *iterons*, which are small repetitive DNA sequences located near the Rep gene, usually in tandem, play a crucial role in the control of plasmid replication in many plasmids (Chattoraj, 2000; Wegrzyn et al., 2016). These sequences have been bioinformatically identified in some *A. baumannii* plasmids; therefore, we searched for the presence of these sequences in the representative Rep genes and their surrounding sequences in each of the new GR groups (GR24-GR33) (Lean and Yeo, 2017; Salto et al., 2018). We could identify such tandem repeats near the initial codon of the Rep protein in six of these groups (GR24, GR26-GR30). The putative iterons of each one of the new groups are shown in Supplementary Table S3. Interestingly, in these cases, we also identified a region rich in A+T near these tandem repeats, which is a typical characteristic of plasmid replication origins. Of course, these presumptions must be tested in the laboratory. In contrast, GR25, GR31, GR32, and GR33 do not have iterons, at least not near the Rep gene. The first two Rep genes belong to the Rep_PriCT family and GR33 is the only member of the RepC family in our collection. Plasmid pD36-4 is a bireplicon that encodes two Rep proteins of the Rep_3 superfamily: RepA1 (WP_000140303.1) (GR31) and RepA2 (protein_id WP_000786839.1) (Hamidian and Hall, 2018a). The RepA2 gene is preceded by three copies of a 19 bp iteron sequence, but surprisingly, RepA1 does not possess iterons at least 500 bp upstream of the initiation codon, 500 bp downstream of the stop codon or within the Rep coding region, suggesting that this protein is no longer responsible for pD36-4 replication(Hamidian and Hall, a).

### Plasmid Incompatibility and Initiator Proteins

Plasmid incompatibility has been defined as the inability of two replicons to coexist in the same cell line. This phenomenon occurs when some elements of the replication or partitioning machineries of a plasmid interfere with the maintenance functions of a second plasmid (Novick, 1987; Austin and Nordström, 1990). Thus, different plasmids that are stably maintained in the same bacterial cell belong, by definition, to different incompatibility groups. On the other hand, plasmids that are mutually incompatible are classified within the same incompatibility group and, very frequently, are phylogenetically closely related.

An inspection of the initiator genes in each of the plasmid lineages with only one Rep gene shows that all members of the same plasmid lineage share a replication initiator protein, classified within the same Rep homology group as defined by Bertini and coworkers (Bertini et al., 2010). For example, Rep proteins of lineage 1 belong to Rep group GR6; Rep proteins of lineage 2 belong to GR2; Rep proteins of lineage 3 belong to GR24, and those of LN_5 are classified within GR25 (Supplementary Table S1). In many cases, Rep proteins of the same GR group have amino acid sequences that are identical or almost identical: for example, all Rep proteins within LN_2 or LN_3A are identical, and those of LN_1 share 99.1% sequence identity among each other.

Plasmid lineages (LN_8, LN_10, LN_16) share Rep proteins of the same GR homology group (GR3); however, a protein alignment performed with Clustal Omega indicates that Rep proteins of LN_8 and LN_10 are almost identical (>99.6%). Differences between LN_8 and LN_10 and LN16 is 80.2%. In our collection, members of these plasmid lineages are never located in the same bacterial isolate; however, differences in the sequences of Rep proteins between these two groups could be significant enough to represent two incompatibility groups. Recently, Blackwell and Hall (2019) showed that plasmids pS32-1 and pS21-a are compatible and that these plasmids contain Rep proteins of the Rep_3 superfamily. Interestingly, these proteins share a protein sequence identity of 85.4% (Blackwell and Hall, 2019). Nevertheless, an experimental approach is needed to resolve these problems. Our analysis indicates that plasmids of the same isolate belong to different plasmid lineages, with two exceptions, namely, isolates CR17 and CS01, which are almost identical in sequence. Each isolate possesses three plasmids, and two of the plasmids in each strain belong to LN_1; however, we were unable to identify complete Rep genes in these four plasmids; we could identify only truncated Rep genes or pseudogenes. Therefore, we could not elucidate the mechanisms via which these plasmids replicate or coexist in the same isolates. Taken together, these observations suggest that members of a plasmid lineage belong to the same incompatibility group.

### Bi- and Trireplicons

It has been previously observed that some *A. baumannii* plasmids contain more than one gene encoding a Rep protein. In fact, there are some examples of such plasmids in our collection: 5 plasmids possess two Rep genes, and one plasmid, p3ABSDF, contains 3 Rep genes (Supplementary Table S4). Each one of the Rep genes residing in the same plasmid belongs to a different Rep group. Some of the isolates that have bi- or trireplicons also contain other companion plasmids; these companion plasmids always include replication modules belonging to different Rep groups, between each other and with those present in the multireplicon plasmid. The French isolate SDF is an extreme example present in our collection. This isolate has three plasmids: p1ABSDF, p2ABSDF and p3ABSDF. The first plasmid possesses a replication module classified within Rep group GR1. The second plasmid, p2ABSDF, has two replication modules, one belonging to GR12 and the other to GR18. The third plasmid has 3 replication modules that belong to different Rep groups: GR7, GR9 and GR15. All the GR homology groups present in each isolate differ, preventing potential functional interference between the groups. These observations reinforce our hypothesis that each plasmid lineage belongs to a different incompatibility group and also suggest that these plasmids are the products of ancient plasmid cointegrations.

We also identified a plasmid lineage, LN_3 with a Rep protein belonging to homology group GR24. Members of this lineage contain a large set of phage-related genes, including several that could be implicated in replication, such as a DNA primase, a DNA helicase, a DNA ligase, the catalytic domain of DNA polymerase III (subunit α), and exonucleases, as already observed by Huang and coworkers (Huang et al., 2014). Plasmids that are capable of using phage-related proteins in replication can be considered bireplicons.

### Partitioning Modules

Plasmids of high molecular weight and low copy number require an active segregation machinery to ensure that newly replicated plasmids are adequately segregated into the daughter cells. To date, three different active segregation machinery types have been identified, all of which consist of an NTPase, a centromere-like binding protein and at least one centromere-like sequence. These segregation machineries have been classified into three types according to their NTPase proteins: type I, which has a Walker-type ATPase (ParA); type II, which contains actin-like ATPases (ParM); and type III, which possesses a GTPase similar to tubulin (TubZ) (Baxter and Funnell, 2014). However, by far, the most common segregation machinery is that belonging to type I. This type consists of three different elements: ParA, a Walker-type ATPase; ParB, which is a centromere-like binding protein; and a DNA centromere-like site (*parS*). These systems are usually organized in an operon in which the first gene is *parA*, followed by *parB*, and the *parS* site is usually located near the *parA/parB* genes. Generally, plasmids that use this segregation system possess only one copy of the operon (Bignell and Thomas, 2001).

Of the *A. baumannii* plasmids studied here, lines LN_1, LN_5, LN_7 and LN_8 have *parA/parB* genes in the classic conformation, but members of LN_8 contain duplicates of these genes. In contrast, other lineages possess incomplete *parA/parB* systems: LN_3 members have one copy of *parA* and two copies of *parB*; LN_11 contains only one *parA* gene per plasmid and LN_18 members contain one *parB* copy. Interestingly, all members of LN_8 also encode a ParM-like protein, suggesting that these plasmids may possess a second segregation system belonging to type II. These observations revealed an extensive diversity of plasmid segregation systems in *A. baumannii* (Supplementary Table S1).

### Toxin-Antitoxin Modules

Plasmids have developed several genetic modules to ensure their persistence within a bacterial population, and some of these modules are classified as toxin-antitoxin (TA) modules. These modules consist of two genes: one encoding a toxin and the other its cognate antitoxin. Toxins are more stable than antitoxins; therefore, cells that lose a plasmid encoding one of these modules are eventually eliminated from the population (Hayes and Van Melderen, 2011; Unterholzner et al., 2013). The presence of these modules on plasmids not only ensures the persistence of the plasmids within a cell line but may also play a role in bacterial virulence (Lobato-Márquez et al., 2016). TA modules been previously described in *A. baumannii* plasmids; therefore, we searched for the presence of these modules in our 173 plasmids (Jurenaite et al., 2013; Sužiedėlienė et al., 2016; Armalytė et al., 2018). We determined that 108 of them have TA modules belonging to nine different classes. Eight of these modules were TA modules of type II: ZetaTA (43.5%), SplTA (30.5%) and HigB/A (11.1%), and other TA modules that were less well represented (13.9%, in total), including YafQ/RelB, RelB/E, HicAB, HipA/B, and Phd/YoeB. Four plasmids have the TA module AbiEii/AbiGii (type IV). Plasmids with TA modules exhibit the general tendency to have one per plasmid, with one exception: the orphan plasmid p3ABAYE has three different TA systems, namely, HigB/A, HipA/B, and RelB/E. Plasmids with the same TA module, in general, do not coexist. We have two isolates, namely, CR17 and CS01, that each possess one plasmid of the same lineage and with the same TA modules (Supplementary Figure S14 and Supplementary Table S5).

Plasmid-carried restriction-modification modules play a role in plasmid stabilization via postsegregational killing (Kulakauskas et al., 1995) therefore, we searched for these modules in the plasmid collection, and only 7.9% of the plasmids harbor these modules. We showed that only five members of LN_1 and two plasmids of LN_3B have these modules. Three orphan plasmids, namely, pOIFC032-101, p2ABSDF and p3ABSDF, also have restriction-modification modules. Some plasmids, such as those belonging to LN_8 and the orphan plasmid pHWBA8_1, encode only for the DNA methyltransferase. These results suggest that some members of LN_1 and LN_3B acquired restriction-modification modules after the origination and diversification of the lineages.

### Conjugation Modules

Conjugation is probably the most efficient process for dissemination of plasmids among strains of the same species or even to not closely related species. This process requires two gene sets: one involved in mating pair formation, which encompasses all genes required for the synthesis of a specialized type 4 secretion system that is essential for establishment of contacts between donor and receptor cells. The second gene set encodes products required for DNA processing and replication. Plasmids with these two functional gene sets are self-transmissible. However, other plasmids, containing only a transfer origin (*oriT*), a relaxase gene and some genes encoding nicking accessory proteins, require for mobilization of their proteins a specialized type 4 secretion system encoded by a second (helper) plasmid. These plasmids are known as mobilizable plasmids (Smillie et al., 2010; Cabezón et al., 2015). We performed a bioinformatic search for genes involved in conjugation in our plasmid collection, and the results are summarized in Supplementary Table S5. We discovered that only two plasmid lineages, namely, LN_1 and LN_5, have large sets of conjugation genes (>10 genes), but only members of LN_1 have been experimentally shown to be capable of conjugation (Di Venanzio et al., 2019). One of the 39 orphan plasmids, pKBN10P02143, has a large set of conjugation genes, suggesting that this plasmid is also conjugative. We also found some plasmids that have a small set of six conjugation genes but not a gene encoding a relaxase, such as members of lineages LN_7 and LN_8, suggesting that in the mobilization capacity was lost during evolution.

**FIGURE 4:**
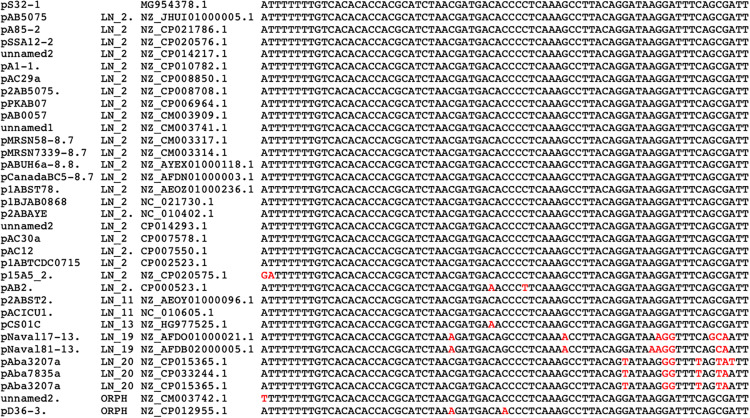
DNA sequence alignment of *oriT* regions (73 bp) located in *A. baumannii* plasmids. At the left, plasmid names followed by the plasmid lineages and by their accession numbers. Letters in red are the nucleotides that show differences with the *oriT* of plasmid pS32.1 (at the top).

Eight of the 21 plasmid lineages identified in this work have the potential to be mobilizable, considering that these lineages have relaxase genes and their cognate *oriT* sequences. Six of these plasmid lineages (LN_12, LN_14, LN_15, LN_17, LN_18 and LN_3B) have relaxase genes belonging to the MOB_*Q*_ family, and all of these genes are closely related to other relaxases described only for *A. baumannii* plasmids (Salto et al., 2018). Lineages LN_1 and LN_5 have relaxase genes of the MOB_*F*_ family, and members of LN_4 possess a relaxase gene of the MOB_*H*_ family. Thirteen orphan plasmids have MOB_*Q*_ relaxase genes and only one relaxase gene of the MOB_*P*_ family. Notably, 14 plasmid lineages do not have relaxase genes; however, some of these lineages are dispersed throughout the A baumannii phylogenetic tree constructed with ribosomal genes not containing recombination signals. However, it has been shown that some *Staphylococcus aureus* plasmids, even in the absence of a relaxase and relaxase accessory genes, have sequences that mimic *oriT* sequences and that can be used for mobilization when they coexist with a conjugative plasmid that encodes Mob proteins able to recognize these oriT sequences (O’Brien et al., 2015a, b). Recently, Blackwell and Hall (2019) showed that the conjugative plasmid (pAb-G7-2) was capable to mobilize plasmid pS32-1, which lacks Mob encoding genes, through a relaxase *in trans* mechanism (Blackwell and Hall, 2019). Making sequence comparisons, these authors suggest that plasmid pS32-1 has a 32 pb DNA sequence that closely matches in sequence and organization the *oriT* of plasmid R388, an IncW plasmid whose *oriT* has been experimentally dissected. Blackwell and Hall also showed that the putative *oriT* and their adjacent sequences are present in other *A*. *baumannii* plasmids (Blackwell and Hall, 2019). To expand these observations, we search for the presence of these sequences in our plasmid collection set and here we show that they are present in all members of LN_2, LN_11, LN_19, LN_20 and some other plasmid, including a couple of orphans, indicating that potentially this mechanism is the responsible to disperse this plasmid lineages through different *A. baumannii* clades. DNA alignment of the putative *oriT* sequences located in these plasmids is shown in Figure 4. Nevertheless, these observations in conjunction also suggest that other plasmid transmission mechanisms that are not dependent on type IV secretion systems, such as transduction, transformation or outer membrane vesicles may play an important role in the spread of plasmids between *A. baumannii* populations (Rumbo et al., 2011; Chatterjee et al., 2017).

All *A. baumannii* strains studied here contain in their chromosomes genes encoding a type VI secretion system (T6SS) that is used to eliminate nonkin bacteria (Weber et al., 2013). An essential requirement for conjugation and T6SS functioning requires a tight cell-to-cell contact, and for this reason, conjugation can only take place when the T6SS is repressed, otherwise, the receptors for conjugation will be killed. Weber et al. (2015) demonstrated that large *A. baumannii* conjugative plasmids, all belonging to LN_1, encode two proteins TetR1 and TetR2 that repress the expression of the T6SS system and in this way promoting the dissemination not only of LN_1 plasmids but also of those mobilizable plasmids that coexist with them (Weber et al., 2015; Di Venanzio et al., 2019). These observations explain why LN_1 plasmids are widely distributed along many *A. baumannii* strains.

### Insertion Sequences

IS elements and transposons are mobile genetic elements that can move from one location to another on the same replicon or between replicons of the same cell, but if linked to other mobile elements such as plasmids or phages, these elements can be horizontally transmitted to other genomes (Siguier et al., 2014). These elements play an essential role in genome plasticity and gene expression and play a crucial role in bacterial pathogens because antibiotic resistance genes are frequently linked to these elements (Partridge et al., 2018). However, 47.3% of the plasmids in our collection do not have IS elements. The remaining plasmids analyzed here have at least one IS element of the 41 different IS elements identified in the collection (Supplementary Table S6). The most common IS elements were IS*Aba1* (13.1%) and IS*Aba125* (12.6%). The plasmid lineages exhibit contrasting features in terms of the number and diversity of IS elements: some plasmid lineages do not contain IS elements, such as LN_2 and LN_4. However, all the members of some lineages have IS elements. Some of these plasmids share the same IS elements or set of IS elements located in the same region (LN_10), while members of other lineages include different IS elements (i.e., LN_7). In conjunction, some lineages have members that lack IS elements; others have members with one IS; and the remaining include several IS elements of different kinds scattered along their DNA sequences. One of these lineages is LN_1. This lineage includes 42 members. Eight of these members do not possess IS elements; 22 members have only one element (the most frequent element being IS*Aba125*); and the remaining members have 2-4 IS elements. This observation clearly shows that IS elements are secondary acquisitions in the genomes of the members of this lineage. The plasmid lineages with a high number of IS elements and which exhibit high diversity in IS families are LN_8 and LN_7.

### XerCD Recombinase and *Pdif* Sites

**FIGURE 5:**
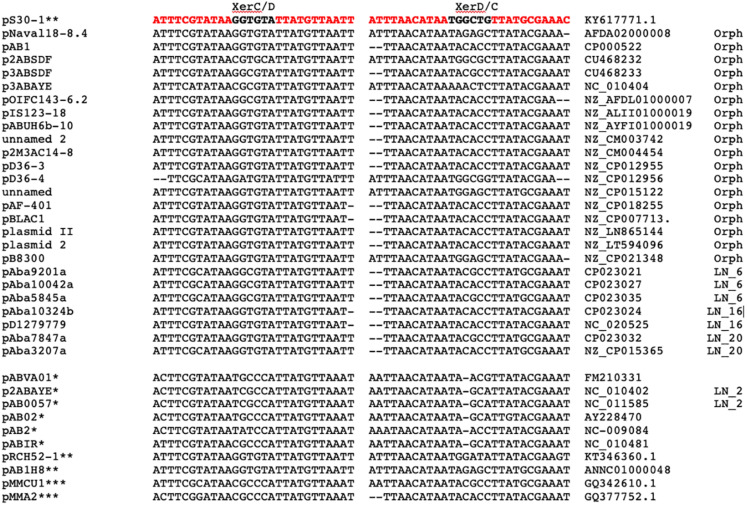
Alignment of left *pdif* (XerC/D) and right *pdif* (XerC/D) plasmids sites (in red) and their flanking sequences. At the top sequences used as query. At the left, plasmid names followed by XerC/D and XerC/D sequences. Next, column list GenBank accession numbers followed by the lineage number. At the bottom and marked with asterisks are plasmid and *pdif* sites defined by other authors: D’Andrea et al. (2009); Merino et al. (2010), and Blackwell and Hall (2017).

XerCD recombinases and their action sites (*dif* or XerC/D and XerD/C sites) have an important role resolving chromosome and plasmid dimers to monomers, but also in other site-specific reactions like the integration of the phage CTX at the *dif1* site of *Vibrio cholerae* chromosome I (Summers and Sherratt, 1988; Val et al., 2005). The presence of homologous *dif* sequences (*pdif*) has been found in many *A. baumannii* plasmids and they consist of stretches of 28 bp that contain the binding sites for the XerC and XerD recombinases (11 bp each) separated by a variable 6 bp linker. It has been proposed that these sites play a role in the mobilization of discrete DNA modules between *A. baumannii* replicons (D’Andrea et al., 2009; Blackwell and Hall, 2017). These modules have an important role in the dissemination of antibiotic resistance genes, since some of them embrace antibiotic-resistant genes like OXA-58 and OXA-24/40 (Poirel and Nordmann, 2006; Merino et al., 2010; Grosso et al., 2012), genes involved in tetracycline resistance (*tet39*), or the *msrE* and *mphE* macrolide resistance genes (Blackwell and Hall, 2017). We evaluated the presence of these sites in our plasmid set using as query the *pdif* sites of plasmid pS30-1 described by Blackwell and Hall (2017). Many plasmids of our collection possess at least one XerC/D site and others, but not necessarily the same plasmids, have one or several XerD/C sites, but only 15 plasmids have matches with both sequences. The list of the plasmids possessing these sites and the DNA sequence alignment of these sites are shown in Figure 5. In this work, we analyzed the *pdif* modules with antibiotic resistance genes. This analysis revealed some of the gene modules described by other authors in new plasmids. For example, the module of plasmid pS30-1 carrying *tetR* and *tet39* genes and involved in tetracycline resistance is also present in the orphan plasmid pNaval18-8.4 (Blackwell and Hall, 2017). However, in plasmids of the Mexican isolates, we found two new *pdif* modules. One of them of 967bp contains an OXA-72 gene and was identified in the members of LN_6. The second was present in plasmids pAba7847a and pAba3207a of LN_20 and consists in a 5260 bp module with four genes: OXA-58, two IS30 family transposases, and a hypothetical protein. However, more work must be done to identify other *pdif* modules carrying genes not related to antibiotic resistance.

### Which Plasmids Carry Antibiotic Resistance Genes?

As vehicles of horizontal gene transfer, plasmids play a crucial role in the dissemination of antibiotic resistance genes within pathogenic bacterial populations (San Millan, 2018; Carattoli, 2013). To evaluate the role of *A. baumannii* plasmids in the dispersion of antibiotic resistance genes, we searched for the presence of acquired resistance genes in our plasmid set using the ResFinder database (Zankari et al., 2012). In this manner, we identified not only plasmids that carry antibiotic resistance genes but also the plasmids lineages associated with these genes (Supplementary Table S7). Only 35.2% of our plasmid collection possesses antibiotic resistance genes, and of these plasmids, thirty-eight contain only one antibiotic resistance gene. Fifteen plasmids have two antibiotic resistance genes, and eight plasmids have three or more of these genes. The most frequent antibiotic resistance genes were those involved in resistance to aminoglycosides, which were present in 60.6% of the plasmids carrying antibiotic resistance, followed by plasmids with genes conferring resistance to beta-lactam antibiotics (49.1%). Sulfonamide resistance genes were also present in 26.2% of the plasmids with antibiotic resistance, and 14.7% have genes implicated in macrolide resistance.

Of the twenty-three plasmid lineages, only thirteen have members with antibiotic resistance genes. However, most commonly, only a few members of a plasmid lineage possess this type of gene, suggesting that these genes were secondary acquisitions after the origination of the lineage. With a few exceptions, antibiotic resistance genes are closely linked to one or two IS elements, in some cases to class 1 integrons, and in three plasmids, namely, pA85-3, pAB04-2 and pUSA15-1, all of which are members of LN_1, the antibiotic resistance genes are linked to an AbaR4 element (Hamidian et al., 2014; Hamidian and Hall, 2018b). A good example of this situation is lineage LN_1. This lineage has 42 members, but only 14 have antibiotic resistance genes, and of these plasmids, nine carry one antibiotic resistance gene; three plasmids have two resistance genes; and plasmid p1AB5075 carries eleven of these genes. One gene is an aminoglycoside resistance gene (*aph(3’)-Via*) surrounded by two IS*Aba25* elements, and the remaining antibiotic resistance genes are class 1 integrons. The other twelve plasmids have antibiotic resistance genes tightly linked to IS*Aba1* or IS*Aba25* elements. These observations suggest that the IS elements and antibiotic resistance genes were acquired after the origin of this plasmid lineage.

The most predominant mechanism for carbapenem resistance in *A. baumannii* is the activity of OXA-type beta-lactamases (serine carbapenemases), some of which are encoded in plasmids (Da Silva and Domingues, 2016). In the analyzed plasmids, we found seven lineages with members carrying *bla*OXA genes: seven members of LN_1 carry *bla*OXA-23 genes as well as two members of LN_5. The four members of LN_6, all obtained from Mexican isolates, have *bla*OXA-72 genes. All members of LN_11 and LN21 possess *bla*OXA-58 genes, and one member of LN_14 and another from LN_17 contain *bla*OXA-24 genes.

### Gene Flux Between Plasmid Lineages

**FIGURE 6:**
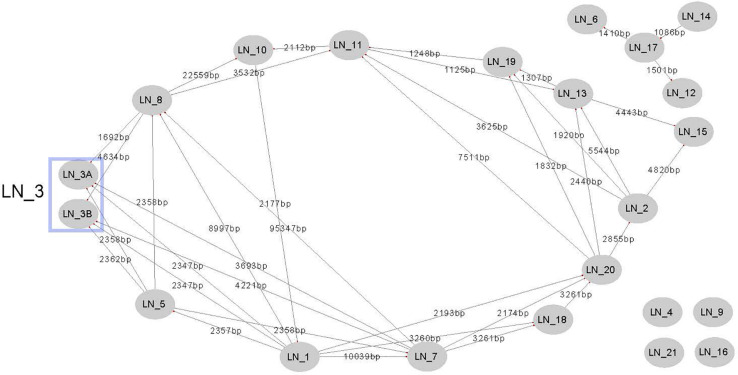
Gene flux between plasmid lineages. Edges connect plasmid lineages that share at least 1Kb of DNA with an identity of 90%. Numbers in edges represent the total amount of different DNA sequences that members of one plasmid lineage have in common with members of the other plasmid lineage.

**FIGURE 7:**
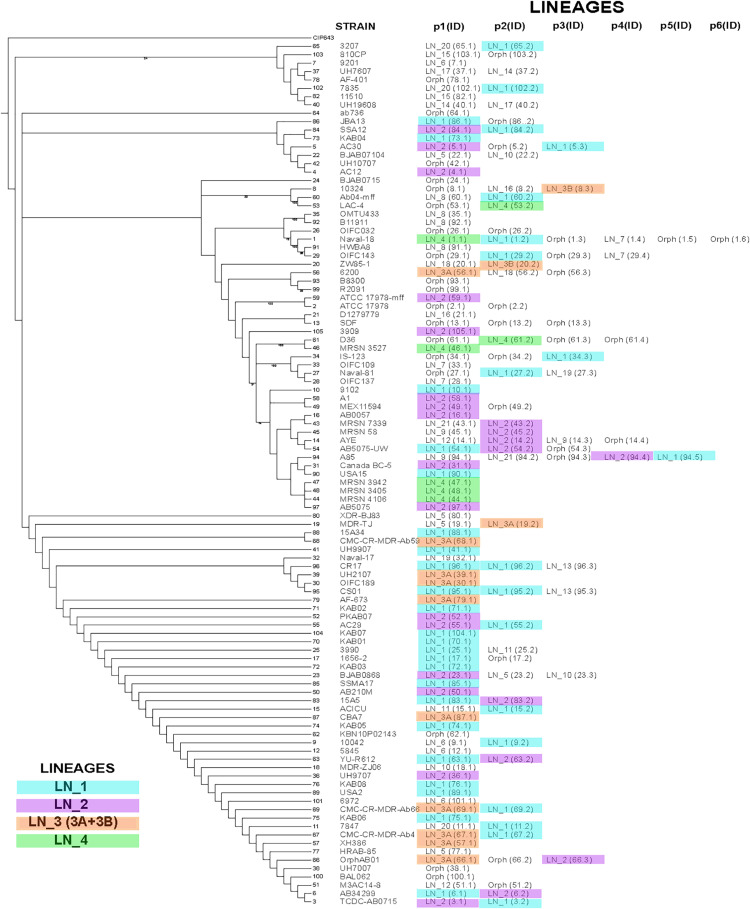
Phylogenetic Tree of strains carrying the plasmids analyzed here. The tree was constructed using unicopy ribosomal protein genes without recombination signals. Bootstrap values higher than 70% are indicated in the tree. Strains containing our plasmid collection harbors a different numbers of plasmids, between one and six (p1..p6). At the right of each strain name shows the plasmids that that particular strain contains, using the alias plasmid name listed in Supplementary Table S1 and the plasmid lineage that they belong. Plasmids marked in color belong to LN_1, LN_2, LN_3 or LN_4. Orph, indicates that the plasmid is an orphan.

To evaluate the gene flux between plasmid lineages or the amount of gene information that is shared between plasmid lineages, we performed BLASTn comparisons using the representative plasmid of one lineage as a query against all plasmids belonging to the other lineages. With this approach, we identified all DNA regions of 1 kb or higher with an identity of at least 90% and recorded the genes that remained in such regions. The results of this analysis are summarized in Supplementary Table S8. The amount of sequence information that two plasmid lineages can share varies dramatically (Figure 6). As described above, the lineage pair LN_7 and LN_8 share at least 90% sequence identity and coverage higher than 50% but lower than 85%. In contrast, lineages LN_4, LN_9, LN_16 and LN_21 do not share DNA sequences higher than 1 kb with any other plasmid lineage. Interestingly, plasmid members of these lineages are embedded in different genomic backgrounds, as illustrated in the phylogenetic tree shown in Figure 7. The remaining lineages share information with at least three and up to seven other plasmid lineages (Supplementary Table S8). Most of the DNA sequences that are shared between plasmid lineages, as expected, contain transposable elements, commonly but not exclusively IS*Aba1* and IS*Aba125*. Sets of antibiotic resistance genes are also frequently shared between plasmid lineages, and these genes are frequently linked to transposable elements such as IS elements and antibiotic resistance islands (AbaR4), suggesting that these elements frequently travel together (Supplementary Table S6).

### Beyond *A. baumannii* and *Acinetobacter*

It has been shown that plasmids play a crucial role in disseminating virulence and antibiotic resistance genes in pathogenic bacteria. However, not all plasmids have the same potential to act as vectors for these purposes. One property that imposes limits on this potential is the replication host range. Some plasmids are capable of replicating in one or a few related species (narrow host range), while others are capable of replicating in an ample range of species and even genera (wide host range) (Jain and Srivastava, 2013). To evaluate the potential plasmid host ranges of the different *A. baumannii* plasmid lineages, we follow two strategies: first, we explored the NCBI nr (nonredundant) database by BLASTp analysis. We searched for proteins identical in sequence to those annotated as Rep proteins in our plasmid collection but excluded those identified in *A. baumannii* or *Acinetobacter*. Second, we also performed a BLASTn analysis of the NCBI nr (nonredundant) database, using the DNA sequences of the representative plasmids of each lineage and all orphan plasmids as queries but, again, excluding matches within *A. baumannii* or within the *Acinetobacter* genus.

A summary of our findings is presented in Supplementary Table S9. The Rep protein that seems to have a broad host range is encoded in the orphan plasmid pAB3 and can be found in the genomes of twelve genera of Gammaproteobacteria, ten genera of Betaproteobacteria, and three genera of Alphaproteobacteria and even in the actinobacterial species *Mycobacteroides abscessus*. This protein belongs to the RepC family. Some replication proteins of the GR3 homology group are also found in a wide variety of bacteria. For example, Rep proteins of the plasmids pB11911 (LN_8) and pMDR-ZJ06 (LN_10) were also identified in twelve different genera, all within Gammaproteobacteria. Similarly, the GR3 Rep protein of the orphan plasmid pHWBA8_1 was also found in the genomes of ten different genera of Gammaproteobacteria. Some Rep proteins of homology group GR2 were identified in, in addition to *A. baumannii, Enterococcus faecium, Klebsiella pneumoniae* and *Providencia rettgeri.* Some other Rep proteins were identified outside of Gammaproteobacteria; for example, some Rep proteins of LN_12 (GR11) and LN_14 (GR27) were located in *Neisseria meningitidis* (Betaproteobacteria). Additionally, the Rep protein of the orphan plasmid pIS123-12 (GR20) was present in the betaproteobacterial species *Nakamurella silvestris*. The remaining Rep proteins of the other GR groups seem to have a limited host range, being found in only *Acinetobacter*.

Intriguingly, some plasmids in our collection are very similar to other plasmids that are not closely related to *Acinetobacter.* For example, pMDR-ZJ06, which is the representative plasmid of LN_10, shares ≥ 75% coverage and 99% DNA sequence identity with the plasmid pEA49-KPC (GenBank: KU318419.1) of *Enterobacter aerogenes*, the plasmid RCS40_p (GenBank: LT985241.1) of *E. coli*, the plasmid pPMK1-NDM (GenBank: NZ_CP008933.1) of *K. pneumoniae* and the plasmid unnamed1 of *Citrobacter* sp. Similarly, plasmid pKP-NCGM38-1 (GenBank: AB825955.1) of *K. pneumoniae* shares 86% coverage and 99% identity with the representative plasmid of LN_13, indicating that this plasmid belongs to LN_13. The representative plasmid of LN_18 is almost identical to the plasmid p3SP-NDM (GenBank: KP900015.1) of *E. aerogenes* strain p3SP and shares 75% coverage and 99% identity with the plasmid p06-1619-NDM (GenBank: KX832928.1) of *P. rettgeri.*

The smallest plasmid in our collection, the orphan plasmid pJBA13_2 (1,109 bp), is almost identical to other very small plasmids belonging to other bacterial classes. This plasmid shares 100% coverage and 100% identity with an unnamed plasmid (GenBank: NZ_CP021055.1) from *Methylobacterium zatmanii* strain PSBB041 and with the plasmid unnamed6 (GenBank: NZ_CP023042.1) from *Komagataeibacter saccharivorans* CV1, both belonging to Alphaproteobacteria. Additionally, with plasmid unnamed2 (GenBank: NZ_CP013938.1) from *Weissella cibaria* strain CMU (Firmicutes) and with unnamed plasmid2 (GenBank: NZ_CP021993.1) from *Cryobacterium* sp. LW097 (Actinobacteria). Finally, orphan plasmid pJBA13_2 is almost identical to plasmids from *Salmonella enterica* subsp. *enterica* serovar Kentucky str. SA20030505 (GenBank: NZ_CP022501.1), *Pantoea ananatis* strain YJ76 (GenBank: NZ_CP022430.1) and *E. coli* strain HB-Coli0 (GenBank: NZ_CP020935.1) (Gammaproteobacteria). These observations indicate that the *A. baumannii* plasmid replication systems vary widely in host range; some seem to replicate only in *Acinetobacter* species, while others are capable of replicating in bacteria of different families and even different bacterial classes.

### Pandemic and Epidemic Plasmids

We took two different approaches to evaluate whether our plasmid lineages are pandemic, that is, capable of existing in a wide range of chromosomal backgrounds, or epidemic, that is, only found in a few closely related chromosomes. For this purpose, we first determined the number of STs (Oxford and Pasteur MLST schemes) containing members of a specific plasmid lineage (listed in Supplementary Table S1). We found that a majority of our plasmid lineages occurred in more than one ST. Moreover, most of the plasmid lineages are present not only in isolates belonging to the International Clones but also out of these clonal complexes. For example, members of LN_1 are present in 20 different STs, and members of LN_2 are present in 9 STs. These lineages are clearly pandemic; however, members of some plasmid lineages seem to be epidemic, considering that these plasmids are restricted to a few STs; for instance, LN_3A, possessing 11 members, is represented in only 3 STs, mostly in ST208, and members of LN_4 are located in 3 STs. Lineages LN_9, LN_11, LN_14, LN_15, and LN_17 are present in one ST, but these lineages have only two or three members each, and in these circumstances, it is not possible to determine whether these lineages have a restricted chromosomal range.

Our second approach was to construct a phylogenetic tree using single-copy ribosomal genes without recombination signals of the strains including our plasmid collection and map the different plasmid lineages in this tree. In Figure 5 we show the locations in the tree of our entire plasmid set, indicating the corresponding plasmid lineages. In Figure 5, we show evidence that the members of the four largest lineages (LN_1 to LN_4) are scattered throughout the phylogenetic tree, indicating that these plasmids are capable of replicating in a wide range of chromosomal backgrounds that are not necessarily closely related. However, notably, despite the wide distribution of plasmids belonging to LN_2 and LN_3A, these plasmids do not possess genes annotated as part of the conjugation or mobilization machineries. Nevertheless, as mentioned above, all members of LN_2 have *oriT*-like sequences that probably can be used for mobilization when they co-reside with a compatible conjugative helper plasmid.

Our bioinformatics analyses suggest that *A. baumannii* plasmids have diverse host ranges: plasmid lineages containing a Rep protein of homology group GR3, have the potential to replicate in an extensive range of bacterial genera, including some important pathogens such as *K. pneumoniae, E. coli*, and *Salmonella enterica.* As described above, the representative plasmid of LN_10 is very similar in sequence and gene content to previously described plasmids of *E. aerogenes, E. coli* and *K. pneumoniae.* All these plasmids of presumably of very wide host ranges are located in not closely related clades in the phylogenetic tree, suggesting that these plasmids were introduced into the *A. baumannii* populations in different independent events. The remaining plasmids seem to replicate only within *Acinetobacter* (restricted host range*).*

A notable feature that we want to point out is the behavior of plasmids as antibiotic resistance gene carriers: members of lineages LN_4, LN_6, LN_7, LN_8, LN_10, LN_11, LN_3B, LN_18 and LN_20 all carry antibiotic resistance genes. As mentioned above, lineages LN_8 and LN_10 can probably also replicate in *K. pneumoniae*, a pathogen that has been identified as an important reservoir of antibiotic resistance genes (Wyres and Holt, 2018). In contrast, lineages LN_2, LN_3A LN_12, LN_13, LN_15, LN_16A, LN_19, and LN_21 do not carry genes of this type. We also found plasmids with intermediate behavior, in which some members of the lineage carry antibiotic resistance genes, while others do not (LN_1, LN_5, LN_14 and LN_17). At least in LN_1 and LN_5, antibiotic resistant genes are closely linked with IS elements.

### Evolution of *A. baumannii* Plasmids in the Nosocomial Environment

Considering all these observations as a whole, we want to propose the following hypothesis to explain the evolution of *A. baumannii* plasmids in the nosocomial environment: before the advent of antibiotics, *A. baumannii* plasmids were parasites of this organism. The gene of these plasmids were involved not only in maintenance functions but also in reducing the fitness cost of plasmid replication. The stability of the structure and gene content of these plasmids over long periods of time in several genetic backgrounds within each of plasmid lineage is probably a product of this condition. When *A. baumannii* arrived in the nosocomial environment, this species began to interact with other bacterial pathogens, such as *K. pneumoniae* or *E. coli*, which already contained plasmids with antibiotic resistance genes. At this point, *A. baumannii* acquired a subset of these plasmids with broad host ranges, probably containing Rep proteins of the homology group GR3. The coexistence of these broad-host-range plasmids with the *A. baumannii* genome allowed the dispersion of new transposable elements with or without antibiotic resistance genes. The acquisition of IS elements permitted some plasticity in *A. baumannii* plasmids. In other words, we propose that at the beginning, *A. baumannii* plasmids were specialized to replicate in this microorganism with a minimal fitness cost, but the acquisition of new broad-host-range plasmids that already contained antibiotic resistance genes native to other microbial pathogens allowed *A. baumannii* to survive easily in the nosocomial environment and become a pathogen of concern.

### A Note Regarding Plasmid Nomenclature

During this study, we found that the nomenclature of *Acinetobacter* plasmids does not follow any type of rule. Moreover, adding an additional layer of complexity, some plasmids do not have official names and are simple referred to in GenBank as unnamed plasmids or tagged as p1, p2, etc. This evident lack of convention imposes unnecessary challenges during a systematic study of plasmids. We need names that easily link a plasmid with its strain/isolate ID and with the species name. For these reasons, we strongly suggest naming *Acinetobacter* plasmids by following the nomenclature rules proposed for the *Agrobacterium* and *Rhizobium* cryptic plasmids: first, all plasmid names must begin with letter “p” followed by the first letter of the genus name and the first two letters of the species name. Then, the strain/isolate ID number is added, followed by a lower-case letter, using “a” for the smallest plasmid, “b” for the next plasmid and so on. For example, the name of the smallest plasmid of *A. haemolyticus* MC1956 would be pAhaMC1956a. The plasmid that is next in size in the same strain will be pAhaMC1956b, and so on.

The annotation of plasmid genes is also confusing and not uniform, and genes are often annotated by using the name of the best BLAST hit and not the true biological function of the gene in the plasmid. Recently, Christopher M. Thomas and coworkers published a paper addressing all these problems and suggested methods to resolve these issues. We encourage scientists interested in plasmid biology to follow those recommendations (Thomas et al., 2017).

## Conclusion

*Acinetobacter baumannii* plasmids belong to a limited number of plasmid lineages and their structure seem to be very stable, in contrast to the observations made in the so-called mosaic plasmids. Mosaic plasmids are composed of genetic elements from distinct sources and they are highly dynamic in acquisition and loss of genes (Pesesky et al., 2019).

Core genomes of *A. baumannii* plasmid lineages contain more genes to those required for plasmid maintenance functions and these genes seems to be not related to the nosocomial environment, open the possibility that they could have other functions and opening the possibility that they reduce fitness cost in the plasmid host. Evidence showed here, suggest that each plasmid lineage represents a plasmid incompatibility group and that the largest plasmid lineages are widely distributed along the phylogenetic tree even though, some of them lack identifiable mobilization systems. In most plasmid lineages transposable elements and antibiotic resistance genes are secondary acquisitions. Plasmids of broad host range have a crucial role in the acquisition of antibiotic resistance genes in *A. baumannii.*

## Materials and Methods

### Plasmid Collection

Our collection included all the complete plasmids (with the “assembled molecule” status) of *A. baumannii* available in the RefSeq and GenBank databases (NCBI) on August 14^*th*^, 2017. We parsed the GenBank and fasta files with the SeqIO Biopython module (Cock et al., 2009) in Python 2.7 for all subsequent analyses.

To increase the diversity of our plasmid collection, we obtained the complete genome sequences of 10 Mexican isolates using the PacBio RSII and Illumina NextSeq platforms. The genome sequences of three isolates, namely, 7804, 810CP and 3207, have previously been reported by some of the authors of this manuscript (Castro-Jaimes et al., 2016; Pérez-Oseguera et al., 2017).

For the other eight isolates, we constructed hybrid assemblies with reads from both platforms using SPAdes v3.9.0 or Unicycler v0.4.1 (Bankevich et al., 2012; Wick et al., 2017). We performed functional annotation with the NCBI Prokaryotic Genome Annotation Pipeline. The GenBank accession numbers of the genomes of the Mexican isolates are listed in Supplementary Table S10. Therefore, in total, we analyzed 173 complete plasmids, and the complete list of plasmids and strains is shown in Supplementary Table S1.

### Plasmid Lineage Delimitation

We performed paired BLASTn (Camacho et al., 2009) searches between all 173 complete plasmids in our collection. We built different plasmid networks, each based on a defined range of coverage (from 40 to 90%). For each plasmid pair, we placed a link between the plasmids if the smallest plasmid covered at least a defined percentage of the other plasmid, where coverage was determined by the sum of alignment lengths with greater than 90% identity Then, we extracted the islands or “connected components” with NetworkX (Hagberg et al., 2008) in Python 2.7. For each connected component, we extracted the most connected plasmid (hub) to use as a reference. When there was more than one hub, we sorted the hubs by size and selected the largest plasmid. The plasmid lineages and the associated references are listed in Supplementary Table S1.

### Extraction of Plasmid Replication Proteins

We used an annotation-based approach to extract the plasmid initiation replication proteins. By using the plasmid GenBank files, we performed a case-insensitive search for the following keywords in the products: “replication protein”, “plasmid replication initiator”, “plasmid replication”, “DNA replication”, “plasmid replicase”, “replication a”, “replication b”, “replication c”, “RepB”, “rolling circle”, “replication initiation”, “replicase”. Then, we extracted both the nucleotide and protein sequences and excluded partial genes and pseudogenes. Additionally, we extracted 500 nucleotides upstream and downstream of the Rep gene for further analyses. This entire process was performed with Python 2.7 and the Biopython SeqIO module (Cock et al., 2009).

### Reference Proteins for Homology Group Designation

We compiled all replication (Rep) proteins that were reported by Bertini et al. (2010); (Bertini et al., 2010) by gene name, plasmid name and plasmid accession number when available. For those cases in which the Rep proteins did not have a locus tag or gene name, we added an artificial locus tag built using the replicon ID and the replicase name. When the replicase name was not available, we assigned the word ‘rep’ followed by a number in the order of appearance in the GenBank file to distinguish between replicases. In some cases, when there were two replication proteins in the same plasmid, to correctly assign these proteins as references for certain homology groups, we performed a BLAST search of these proteins against the GenBank nr database to identify corresponding hits outside the *Acinetobacter* genus reported in Supplementary Table S1 in Bertini et al. (2010). Two proteins could not be identified: the Aci3 replicase from plasmid Ab599 (member of GR3), because the plasmid sequence was not deposited in databases, and the Aci2 replicase from the MAD plasmid, because the plasmid had a partial sequence that did not include the replicase. Therefore, we omitted these proteins from our analyses and examined other members of the same homology groups instead. As reported by Lean and Yeo (2017), the GR2 homology group should be split into two groups; therefore, we separated the proteins that represent GR2 from those of the newly formed GR20. Additionally, (Cameranesi et al., 2017) recently reported new homology groups; thus, we downloaded the plasmids that harbored the replicases that represent these groups and extracted those genes. Supplementary Table S2 lists all proteins used as references in this work, including the origins, accessions, numbers and headers used in the multi-FASTA files included in Supplementary Materials S1, S2.

### Homology Group Assignation for Rep Proteins

First, we performed paired BLASTn (Camacho et al., 2009) searches between all genes encoding replication initiation proteins present in our plasmid collection. We retained hits with more than 74% nucleotide identity and that covered at least 90% of the query. Then, if the query coding sequence (CDS) mapped to only one homology group, we designated the sequence as belonging to that group, whereas if there was more than one hit for different homology groups, we assigned the query to the GR with the highest percentage identity. We discarded the GR23 homology group because the associated reference (KY984047_repAci23) was 100% identical to one of the references of GR8 (GU979000.1_p11921_repA).

### Plasmid Rep Protein Phylogenetic Analysis and Designation of New Homology Groups

We built a network in which each gene encoding a Rep protein was connected to another if the two genes shared at least 74% nucleotide identity and 90% coverage. Then, for the islands or connected components that did not have a Rep protein in the reference table, we selected the hub as a reference and added it to Supplementary Table S1. Additionally, we built plasmid replication initiation protein phylogenies to validate current assignations and new homology groups. We searched for the associated Pfam domains in the Pfam database (El-Gebali et al., 2019), accessed on February 21^*st*^, 2018, to separate the proteins by conserved domains and perform alignments separately because these proteins are very different. We used Clustal Omega (Sievers et al., 2014) to align amino acids and RevTrans (Wernersson and Pedersen, 2003) to guide the nucleotide alignment by the translated CDS. Then, we ran jModelTest2 (Darriba et al., 2012) to search for an adequate evolutionary model and built the phylogenetic tree with PHYML (Guindon and Gascuel, 2003) with the selected model. By visual inspection, we validated the references of new homology groups, selected proteins that may be representative of new clades and designated these proteins as new homology groups, as detailed in Supplementary Table S2.

### Phylogenetic Analysis of Ribosomal Proteins and MLST

We used Roary (Page et al., 2015) to extract monocopy genes encoding ribosomal proteins belonging to the core genome and that had the exact same size in all the strains to avoid gaps in the alignment. We aligned the ribosomal proteins with Clustal Omega (Sievers et al., 2014) to guide the nucleotide alignment with RevTrans (Wernersson and Pedersen, 2003). We discarded sequences with recombination signals detected with RDP4 (Martin et al., 2015). We concatenated the remaining nucleotide alignments with FASconCAT-G (Kück and Longo, 2014) and used jModelTest2 (Darriba et al., 2012) to select the evolutionary model to build a phylogenetic tree with PHYML (Guindon and Gascuel, 2003). We used the ribosomal proteins of the *Acinetobacter haemolyticus* CIP 64.3 strain as an outgroup. The sequence type (ST) assignation of each *A. baumannii* isolate, under Oxford and Pasteur MLST schemes, were obtained from the PubMLST database^1^ (Bartual et al., 2005; Diancourt et al., 2010).

### Identification of Secretion Systems, Antibiotic Resistance Genes, and Insertion Sequences on Plasmids

We used MacSyFinder with the TXSSCAN profiles (Abby and Rocha, 2017) to identify secretion systems on the plasmid collection and ResFinder to identify the acquired antibiotic resistance genes present in our plasmid set (Zankari et al., 2012). We identified the insertion sequence (IS) elements present in the plasmids using the ISfinder database at^2^ (Siguier et al., 2006).

### Identification of *pdif* Sites (XerC/D and Xer D/C) on Plasmids

To identify the *pdif* sites in our plasmid set, we made a BLASTn analysis using as queries the *pdif* sites of plasmid pS30-1: XerC/D, ATTTCGTATAAGGTGTATTAT- GTTAATT and XerD/C, ATTTAACATAATGGCTGTTATGCGAAAC (Blackwell and Hall, 2017).

### COG Assignments

We determined homologous gene assignments for each plasmid based on hidden Markov model (HMM) searches using the *hmmsearch* program (Eddy, 2011). This HMM search process employs a previously constructed model set that represents each of the 4873 COGs and 8539 Remained Orthologous Groups (ROGs) (Tatusov et al., 2003; Taboada et al., 2010). Then, using Perl scripts, we classified each assigned COG by using the general classification scheme of Tatusov [66]. We calculated the frequency of each gene per class and plotted the results using ggplot2 R scripts^3^ (Wickham, 2009).

## Data Availability Statement

Genome sequences were deposited in NCBI/GenBank with the following accession numbers: Isolate 7847: NZ_CP023031.1, CP023032.1, CP023033.1. Isolate 7835: CP033243.1, CP03 3244.1, CP033245.1. Isolate 9102: CP023029.1, CP023030.1. Isolate 5845: NZ_CP023034.1, CP023035.1. Isolate 10042: NZ_CP023026.1, CP023027.1, CP023028.1. Isolate 10324: NZ_CP023022.1, CP023023.1, CP023024.1, CP023025.1. Isolate 9201: NZ_CP023020.1, CP023021.1.

## Author Contributions

MC conceived, designed, and coordinated the study. ÁP-O made plasmid profile analysis and genome analysis of Mexican isolates. AS-C and SC-J made genome assemblies, genome annotation, network analysis, and bioinformatics analysis, and made bioinformatic analysis. AS-C designed figures and most of the tables of the manuscript. R-MG-R made COG analysis and statistics. LA-P made the analysis of Rep proteins. LL made bioinformatic analysis and made many pf Perl scripts used in this work. PV contributed with the Mexican isolates, participated in the manuscript drafting and in the general discussion. SC-R and JS-S had a crucial role in the general discussion. All authors contributed to manuscript revision, read and approved the submitted version.

## Conflict of Interest

The authors declare that the research was conducted in the absence of any commercial or financial relationships that could be construed as a potential conflict of interest.

## Footnotes

1 https://pubmlst.org

2 http://www-is.biotoul.fr

3 http://www.R-project.org/

## Supplementary Material

FIGURE S1 Plasmid networks.

FIGURE S2 Circular maps of members of plasmid lineage LN_3.

FIGURE S3 Circular maps of members of plasmid lineages LN_4 and LN_5.

FIGURE S4 Circular maps of members of plasmid lineages LN_6 and LN_7.

FIGURE S5 Circular maps of members of plasmid lineage LN_8.

FIGURE S6 Circular maps of members of plasmid lineages LN_9, LN_10.

FIGURE S7 Maps of members of plasmid LN_11 and LN_12.

FIGURE S8 Linear maps of members of plasmid lineages LN_13, and LN_14.

FIGURE S9 Linear maps of members of plasmid lineages LN_15, and LN_16.

FIGURE S10 Maps of members of plasmid lineages LN_17 and LN_18.

FIGURE S11 Linear maps of members of plasmid lineages LN_19, and LN_20.

FIGURE S12 Linear maps of members of plasmid lineage LN_21.

FIGURE S13 Phylogenetic tree of Rep proteins belonging to the Pri_CT family.

FIGURE S14 Phylogenetic Tree of strains carrying the plasmids analyzed here and their associated Toxin-Antitoxin modules.

TABLE S1 List of plasmids belonging to lineages and their general characteristics.

TABLE S2 Replicases used to assign GR homology groups.

TABLE S3 Replicon-associated iterons identified in new GR members.

TABLE S4 Plasmids encoding two or more replication proteins.

TABLE S5 Conjugation genes and Toxin-Antitoxin system present in plasmids.

TABLE S6 IS elements present in plasmids.

TABLE S7 Antibiotic resistance genes located in plasmids.

TABLE S8 Gene flux between plasmid lineages.

TABLE S9 Replication proteins of A. baumannii plasmids which are identical in sequence to replication proteins in the genomes of other Acinetobacterspecies and in other genera.

TABLE S10 GenBank accession numbers of Mexican isolates and their Sequence type following Oxfordand Pasteur schemes.

MATERIAL S1 List in fasta format of genes encoding representative replication proteins of each one of the GR homology groups.

MATERIAL S2 List in fasta format of the representative replication proteins of each one of the GR homology groups.
