# Supplementary material for: Structure and Evolution of Acinetobacter baumannii Plasmids
Source: Front Microbiol. 2020 Jun 18;11:1283. doi: 10.3389/fmicb.2020.01283 (PMC7315799; doi:10.3389/fmicb.2020.01283)
Supplement: FIGURE S1 — Plasmid networks. [file Data_Sheet_1.PDF]

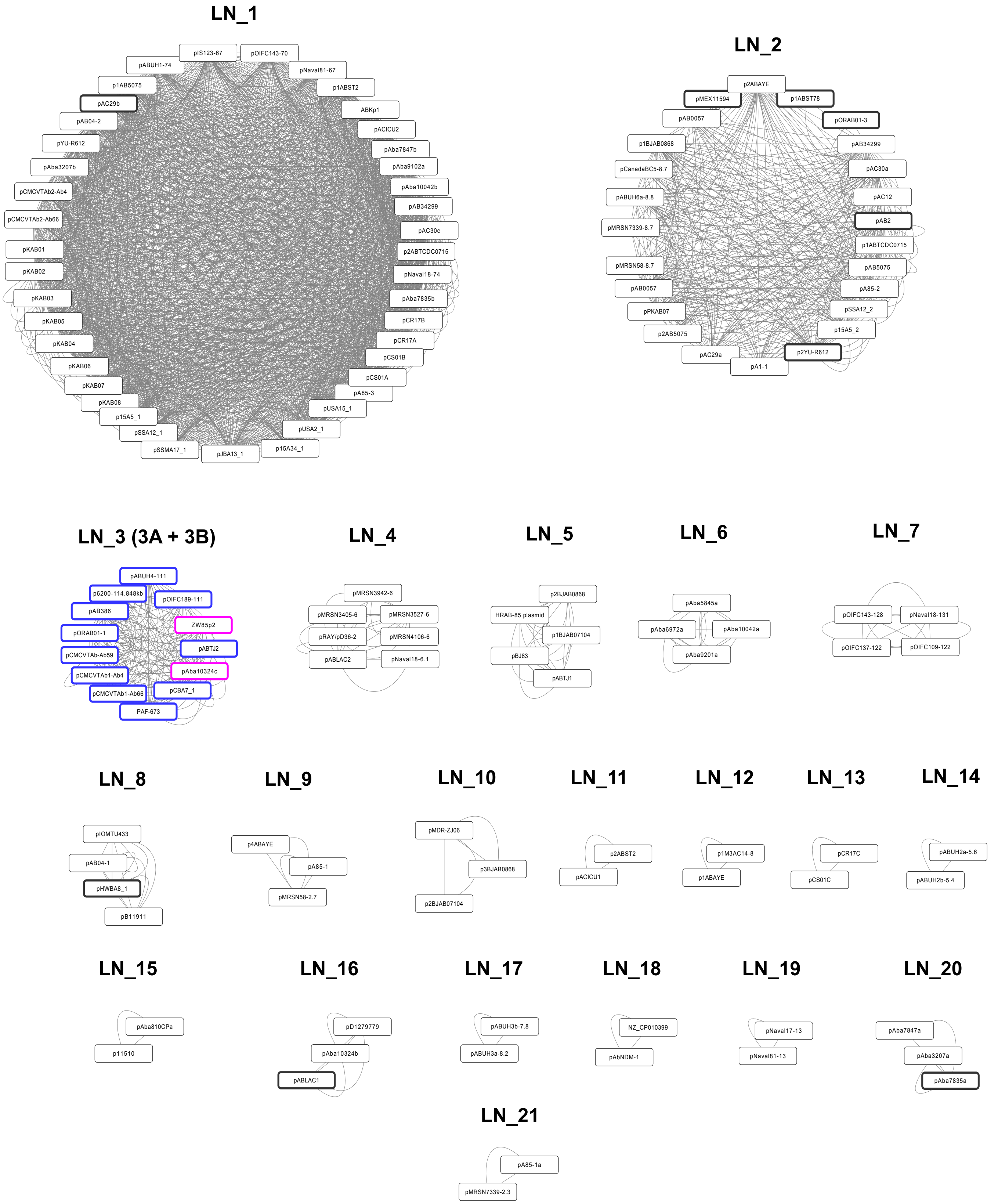

LN_1
pIS123-67
pOIFC143-70
pABUH1-74
pNaval81-67
p1AB5075
p1ABST2
pAC29b
ABKp1
pAB04-2
pACICU2
pYU-R612
pAba7847b
pAba3207b
pAba9102a
pCMCVTAb2-Ab4
pAba10042b
pAB34299
pCMCVTAb2-Ab66
pAC30c
pKAB01
p2ABTCDC0715
pKAB02
pNaval18-74
pKAB03
pAba7835b
pKAB05
pCR17B
pKAB04
pCR17A
pKAB06
pCS01B
pKAB07
pCS01A
pKAB08
pA85-3
pUSA15_1
p15A5_1
pUSA2_1
pSSA12_1
pSSMA17_1
p15A34_1
pJBA13_1
LN_2
p2ABAYE
pMEX11594
p1ABST78
pAB0057
pORAB01-3
p1BJAB0868
pAB34299
pCanadaBC5-8.7
pAC30a
pABUH6a-8.8
pAC12
pAB2
pMRSN7339-8.7
p1ABTCDC0715
pMRSN58-8.7
pAB5075
pAB0057
pA85-2
pPKAB07
pSSA12_2
p15A5_2
p2AB5075
p2YU-R612
pAC29a
pA1-1
LN_3 (3A + 3B)
pABUH4-111
p6200-114.848kb
pOIFC189-111
pAB386
ZW85p2
pORAB01-1
pABTJ2
pCMCVTAb-Ab59
pCMCVTAb1-Ab4
pAba10324c
pCMCVTAb1-Ab66
pCBA7_1
PAF-673
LN_4
pMRSN3942-6
pMRSN3405-6
pMRSN3527-6
pRAY/pD36-2
pMRSN4106-6
pABLAC2
pNaval18-6.1
LN_5
p2BJAB0868
HRAB-85 plasmid
p1BJAB07104
pBJ83
pABTJ1
LN_6
pAba5845a
pAba6972a
pAba10042a
pAba9201a
LN_7
pOIFC143-128
pNaval18-131
pOIFC137-122
pOIFC109-122
LN_8
pIOMTU433
pAB04-1
pHWBA8_1
pB11911
LN_9
p4ABAYE
pA85-1
pMRSN58-2.7
LN_10
pMDR-ZJ06
p3BJAB0868
p2BJAB07104
LN_11
p2ABST2
pACICU1
LN_12
p1M3AC14-8
p1ABAYE
LN_13
pCR17C
pCS01C
LN_14
pABUH2a-5.6
pABUH2b-5.4
LN_15
pAba810CPa
p11510
LN_16
pD1279779
pAba10324b
pABLAC1
LN_17
pABUH3b-7.8
pABUH3a-8.2
LN_18
NZ_CP010399
pAbNDM-1
LN_19
pNaval17-13
pNaval81-13
LN_20
pAba7847a
pAba3207a
pAba7835a
LN_21
pA85-1a
pMRSN7339-2.3

**Supplementary Figure 1.** Plasmid networks. Each one of the networks show here represent a plasmid lineage. Members marked with thick margins have a relationship of at least 50% coverage and 90% of DNA sequence identity with other members of the lineage. The rest of the plasmids in each lineage have at least 85% of coverage and 90% of DNA sequence identity. Edges link plasmid pairs fulfilling these rules. Plasmids of LN_3 marked with blue margins belong to LN_3A. Those marked with pink margins belong to LN_3B.

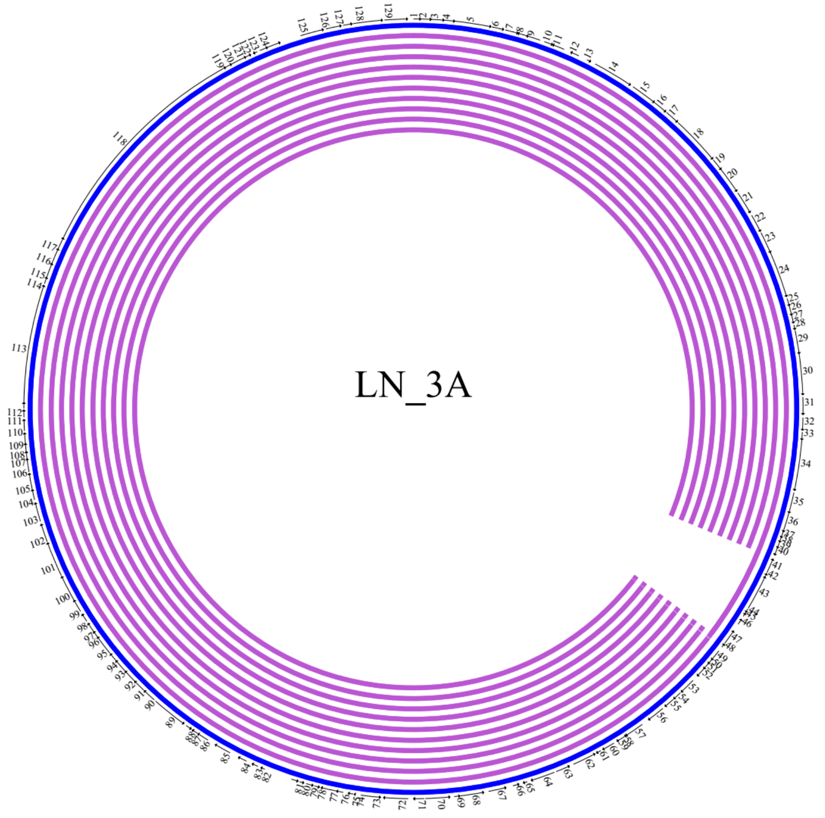
LN_3A

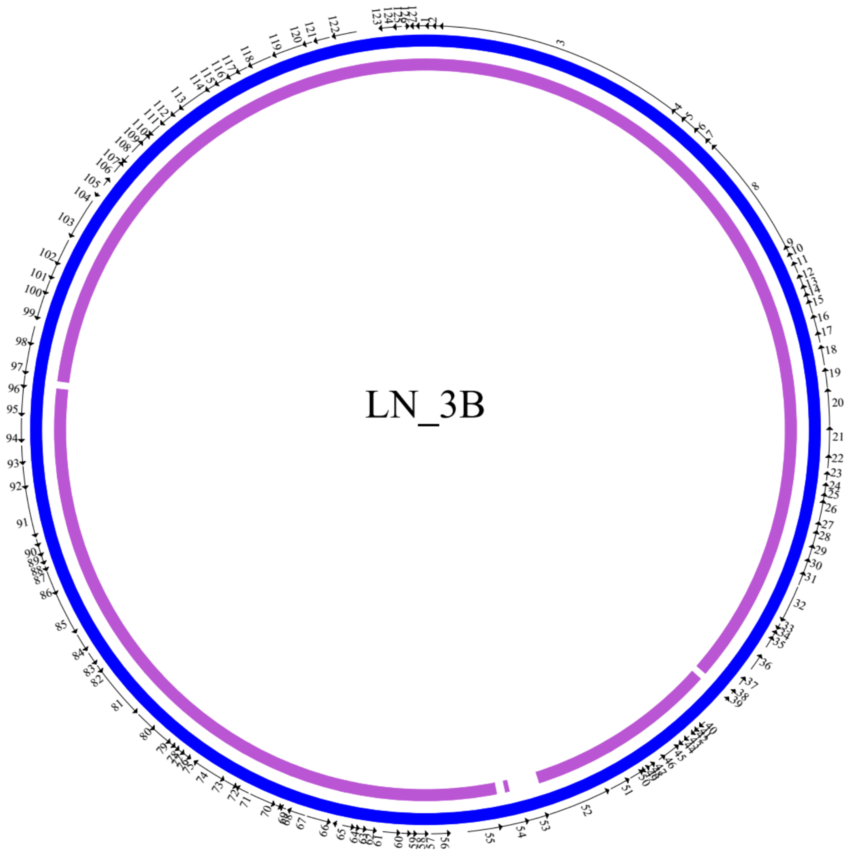
LN_3B

**Supplementary Figure 2.** Circular maps of members of plasmid lineages LN_3A and LN_3B. LN_3A. Blue ring (representative plasmid of the lineage) p6200-114.848kb. Core genes are shown in bold letters. Genes of this plasmid**: 1. Hypothetical protein; 2. Hypothetical protein; 3. Hypothetical protein; 4. Hypothetical protein; 5. DNA ligase; 6. Guanylate kinase; 7. Hypothetical protein; 8. Hypothetical protein; 9. Partition protein ParA; 10. Hypothetical protein; 11. Integrase; 12. Transcriptional regulator; 13.Hypothetical protein; 14. Hypothetical protein; 15. Porphyrin biosynthesis protein; 16. Hypothetical protein; 17. Hypothetical protein; 18. DNA polymerase III subunit Alpha; 19. ; Hypothetical protein; 20. Hypothetical protein; 21. Hypothetical protein; 22. Hypothetical protein; 23. 5'-3' exonuclease; 24. Recombinase RecA; 25. Hypothetical protein; 26. Hypothetical protein; 27. Hypothetical protein; 28. Hypothetical protein; 29. Serine/threonine protein phosphatase; 30. DNA recombination protein RecF; 31. Hypothetical protein; 32. Hypothetical protein; 33. Hypothetical protein; 34. Ribonucleoside-diphosphate reductase subunit Alpha; 35. Ribonucleotide-diphosphate reductase subunit beta; 36. Hypothetical protein; 37. Hypothetical protein; 38. Hypothetical protein; 39. Hypothetical protein; 40. Nucleotide pyrophosphohydrolase**; 41. IS6 family transposase; 42. Response regulator; 43. Lipid A phosphoethanolamine transferase; 44. Hypothetical protein; 45. Hypothetical protein; 46. Hypothetical protein; 47. Hypothetical protein; 48. IS5/IS1182 family transposase; **49. DNA polymerase III subunit épsilon; 50. Hypothetical protein; 51. Hypothetical protein; 52. Hypothetical protein; 53. Hypothetical protein; 54. Hypothetical protein; 55. Hypothetical protein; 56. Hypothetical protein; 57. IS5/IS1182 family transposase; 58. Dehydrogenase; 59. Hypothetical protein; 60. TetR family transcriptional regulator; 61. Hypothetical protein; 62. Alkene reductase; 63. Dimethylallyltransferase; 64. NADH-dependent alcohol dehydrogenase; 65. hypothetical protein; 66. Antibiotic biosynthesis monooxygenase; 67. S-formylglutathione hydrolase; 68. S-(hydroxymethyl)glutathione dehydrogenase; 69. Hypothetical; 70. IS3 family transposase; 71. Hypothetical protein; 72. Glutathione-dependent formaldehyde dehydrogenase; 73. IS5/IS1182 family transposase; 74. tRNA-Asn; 75. Hypothetical protein; 76. Hypothetical protein; 77. Hypothetical protein; 78. Hypothetical protein; 79. Hypothetical protein; 80. Hypothetical protein; 81. Hypothetical protein; 82. Hypothetical protein; 83. Hypothetical protein; 84. Hypothetical protein; 85. Hypothetical protein; 86. Peptidoglycan-binding protein LysM; 87. Hypothetical protein; 88. Hypothetical protein; 89. Helicase; 90. Partitition protein ParB; 91. Hypothetical protein; 92. Partitition protein ParB; 93. ABC transporter; 94. Thiol reductase thioredoxin; 95. Hypothetical protein; 96. Hypothetical protein; 97. Hypothetical protein; 98. Hypothetical protein; 99. Hypothetical protein; 100. Terminase; 101. Hypothetical protein; 102. Hypothetical protein; 103. Hypothetical protein; 104. Hypothetical protein; 105. Hypothetical protein; 106. Hypothetical protein; 107. Hypothetical protein; 108. Hypothetical protein; 109. Hypothetical protein; 110. Hypothetical protein; 111. Hypothetical protein; 112. Hypothetical protein; 113. Tail length tape measure protein; 114. Tail protein; 115. Phage minor tail protein L; 116. Tail protein; 117. Tail assembly protein; 118. Tail protein; 119. Hypothetical protein; 120. Hypothetical protein; 121. Hypothetical protein; 122. Hypothetical protein; 123. Hypothetical protein; 124. Lysozyme; 125. RepB family plasmid replication initiator; 126. Hypothetical protein; 127. Hypothetical protein; 128. DNA helicase; 129. DNA primas**e. Purple rings: from outside to inside: pABTJ2, pOIFC189-111, pABUH4-111, pAB386, pORAB01-1, pCMCVTAb1-Ab4, plasmid_2 CMC-CR-MDR-Ab59, plasmid CMC-CR-MDR-Ab66, PAF-673, pCBA7_1.

LN_3B. The representative plasmid of this lineage is ZW85p2 (blue ring): and its genes are: **1. Hypothetical protein; 2. Hypothetical protein; 3. Phage-related protein, tail omponent; 4. Tail assembly protein; 5. Phage tail protein; 6. Phage minor tail protein L; 7. Tail protein; 8. Prophage LambdaSo, tail length tape measure protein; 9. Hypothetical protein; 10. Hypothetical protein; 11. Hypothetical protein; 12. Hypothetical protein; 13. Hypothetical protein; 14. Hypothetical protein; 15. Hypothetical protein; 16. Hypothetical protein; 17. Hypothetical protein; 18. Hypothetical protein; 19. Hypothetical protein; 20. Hypothetical**

**protein; 21. Terminase; 22. Hypothetical protein; 23. Hypothetical protein; 24. Hypothetical protein; 25. Hypothetical protein; 26. Hypothetical protein; 27. Thiol reductase thioredoxin; 28. ABC transporter; 29. Partition protein ParB; 30. Hypothetical protein; 31. Partition protein ParB; 32. Hypothetical protein; 33. Hypothetical protein; 34. Hypothetical protein; 35. Hypothetical protein; 36. Hypothetical protein; 37. Hypothetical protein; 38. Hypothetical protein;** 39. Hypothetical protein; **40. Hypothetical protein; 41. Hypothetical protein; 42. Hypothetical protein; 43. Hypothetical protein; 44. Hypothetical protein; 45. Hypothetical protein; 46. Hypothetical protein; 47. Hypothetical protein; 48. tRNA-Asn; 49. Hypothetical protein; 50. Hypothetical protein; 51. IS5/IS1182 family transposase; 52. Hypothetical protein;** 53. Hypothetical protein; 54. Restriction modification system DNA subunit R; **55. Type I restriction-modification system subunit M; 56. Hypothetical protein; 57. Hypothetical protein; 58. Hypothetical protein; 59. Hypothetical protein; 60. Hypothetical protein; 61. Hypothetical protein; 62. Hypothetical protein; 63. Hypothetical protein; 64. DNA polymerase III subunit épsilon; 65. IS5/IS1182 family transposase; 66. IS4 family transposase; 67. Sulfonamide-resistant dihydropteroate synthase Sul2; 68. Phosphoglucosamine mutase; 69. Hypothetical protein; 70. Tetracycline efflux MFS transporter Tet(B); 71. Transposase; 72. Hypothetical protein; 73. IS91 family transposase; 74. IS6 family transposase; 75. nucleotide pyrophosphohydrolase; 76. Hypothetical protein; 77. Hypothetical protein; 78. Hypothetical protein; 79. Hypothetical protein; 80. Ribonucleotide-diphosphate reductase subunit beta; 81. Ribonucleoside-diphosphate reductase subunit Alpha; 82. Hypothetical protein; 83. Hypothetical protein; 84. Hypothetical protein; 85. Hypothetical protein; 86. Hypothetical protein; 87. Hypothetical protein; 88. Hypothetical protein; 89. Hypothetical protein; 90. Hypothetical protein; 91. RecA protein; 92. Hypothetical protein; 93. Hypothetical protein; 94. Hypothetical protein; 95. Hypothetical protein**; 96. Hypothetical protein; **97. DNA polymerase III subunit Alpha; 98. Hypothetical protein; 99. DNA polymerase III subunit Alpha; 100. Hypothetical protein; 101. Hypothetical protein; 102. porphyrin biosynthesis protein; 103. Hypothetical protein; 104. Hypothetical protein; 105. Transcriptional regulator; 106. Integrase; 107. Hypothetical protein; 108. Partition protein ParA; 109. Hypothetical protein; 110. Hypothetical protein; 111. Guanylate kinase; 112. Hypothetical protein; 113. Hypothetical protein; 114. Hypothetical protein; 115. Hypothetical protein; 116. Hypothetical protein; 117. Hypothetical protein; 118. Hypothetical protein; 119. DNA helicase; 120. Hypothetical protein; 121. Hypothetical protein; 122. RepB family plasmid replication initiator protein; 123. Hypothetical protein; 124. Hypothetical protein; 125. Hypothetical protein; 126. Hypothetical protein; 127. Hypothetical protein**. Purple ring: pAba10324c

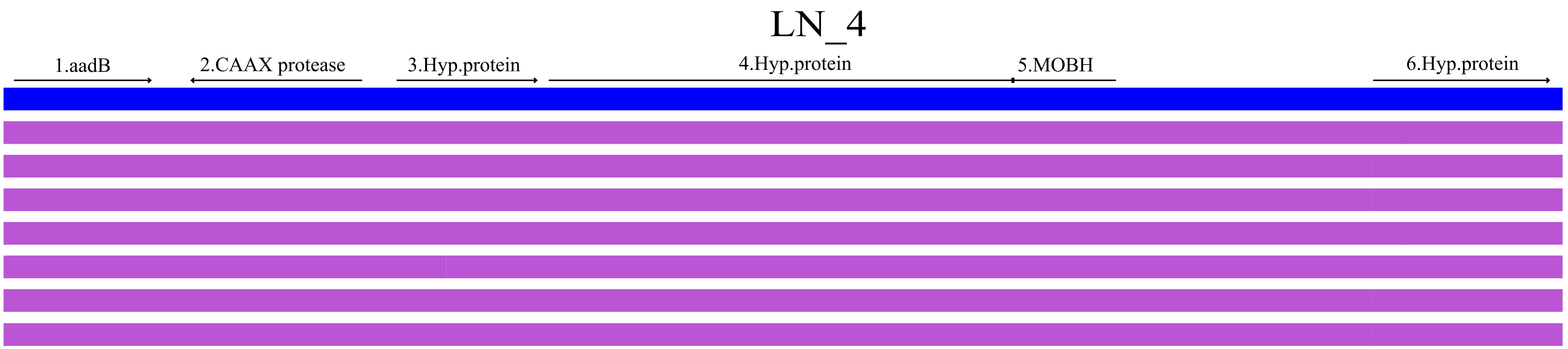
LN_4
1.aadB
2.CAAX protease
3.Hyp.protein
4.Hyp.protein
5.MOBH
6.Hyp.protein

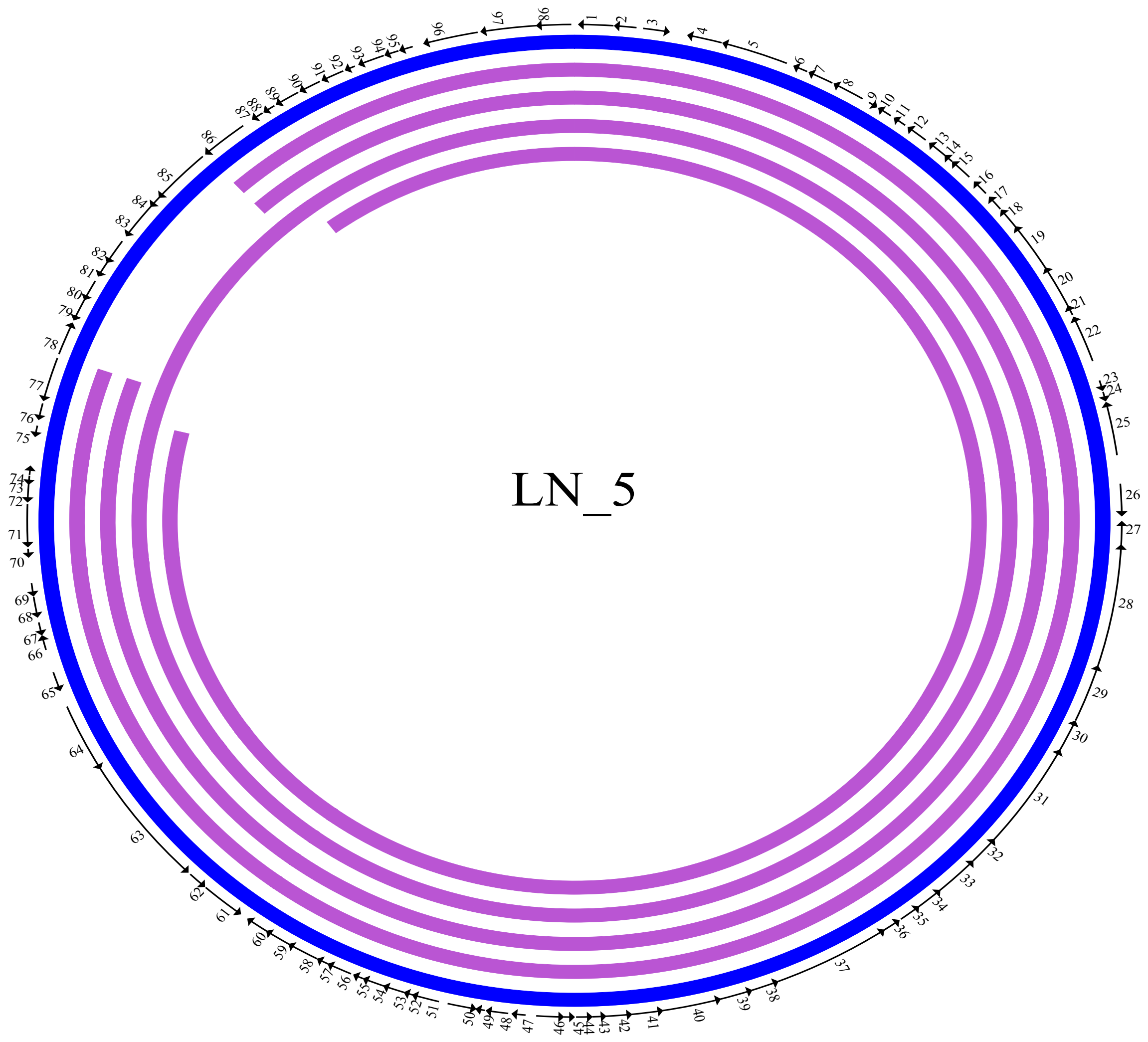
LN_5

**Supplementary Figure 3.** Linear map of members of plasmid lineage LN_4 and circular map of LN_5. LN_4. Blue bar (representative plasmid of the lineage) pABLAC2. Core genes core are indicated in bold letters. Genes of this plasmid: **1. Aminoglycoside nucleotidyltransferase aadB; 2. CAAX protease; 3. Hypothetical protein; 4. Hypothetical protein; 5. Relaxase MobH; 6. Hypothetical protein**. Purple bars: from top to bottom: pALAC4-2, pRAY/pD36-2, pMRSN3405-6, pMRSN3942-6, pMRSN3527-6, pMRSN4106-6, pNaval18-6.1.

LN_5. The representative plasmid of this lineage is pABTJ1 (blue ring): and its genes are **1. SAM-dependent DNA methyltransferase; 2. Hypothetical protein; 3. Hypothetical protein; 4. Hypothetical protein; 5. DNA cytosine methyltransferase; 6. Hypothetical protein; 7. Hypothetical protein; 8. Hypothetical protein; 9. Hypothetical protein; 10. DNA-binding protein; 11. Hypothetical protein; 12. Hypothetical protein; 13. Thermonuclease; 14. Hypothetical protein; 15. Hypothetical protein; 16. Hypothetical protein; 17. Hypothetical protein; 18. Hypothetical protein 19. Hypothetical protein; 20. Zeta toxin; 21. Zeta antitoxin; 22. Plasmid replicase; 23. Hypothetical protein; 24. Hypothetical protein; 25. DNA polymerase V subunit UmuC; 26. Hypothetical protein; 27. Transglycosylase; 28. Conjugal protein TraG; 29. Conjugal protein TraH; 30. Conjugal protein TraF; 31. Conjugal protein TraN; 32. Conjugal protein TrbC; 33. Conjugal protein TraU; 34. Conjugal protein TraW; 35. Peptidase; 36. Hypothetical protein; 37. Conjugal protein TraC; 38. Conjugal protein TraV; 39. Protein disulfide-isomerase; 40. Conjugal protein TraB; 41. Conjugal protein TraK; 42. Conjugal protein TraE; 43. Conjugal protein TraL; 44. Hypothetical protein; 45. Hypothetical protein; 46. Murein transglycosylase; 47. Hypothetical protein; 48. Hypothetical protein; 49. Hypothetical protein; 50. Resolvase; 51. Hypothetical protein; 52. Hypothetical protein; 53. Hypothetical protein; 54. Hypothetical protein; 55. Hypothetical protein; 56. Hypothetical protein; 57. Hypothetical protein; 58. DNA methylase; 59. Hypothetical protein; 60. Hypothetical protein; 61. Hypothetical protein; 62. Hypothetical protein; 63. Relaxase MOBF; 64. Type IV secretory pathway, VirD4; 65. Hypothetical protein; 66. Hypothetical protein; 67. Hypothetical protein; 68. Hypothetical protein; 69. Hypothetical protein; 70. Hypothetical protein; 71. Hypothetical protein; 72. Hypothetical protein; 73. Hypothetical protein; 74. Hypothetical protein; 75. Hypothetical protein**; 76. Hypothetical protein; 77. IS4 family transposase; 78. Carbapenemase blaOXA-23; 79. ATPase; 80. Hypothetical protein; 81. Hypothetical protein; 82. ATPase; 83. Hypothetical protein; 84. Hypothetical protein; 85. Hypothetical protein; 86. IS4 family transposase; **87. Hypothetical protein; 88. Hypothetical protein; 89. Hypothetical protein; 90. Hypothetical protein; 91. Hypothetical protein; 92. Hypothetical protein; 93. Hypothetical protein; 94. Hypothetical protein; 95. Hypothetical protein; 96. Hypothetical protein; 97. ParB family partition protein; 98. ParA family protein**. Purple rings: from outside to inside: p1BJAB07104, p2BJAB0868, plasmid HRAB-85, pBJ83.

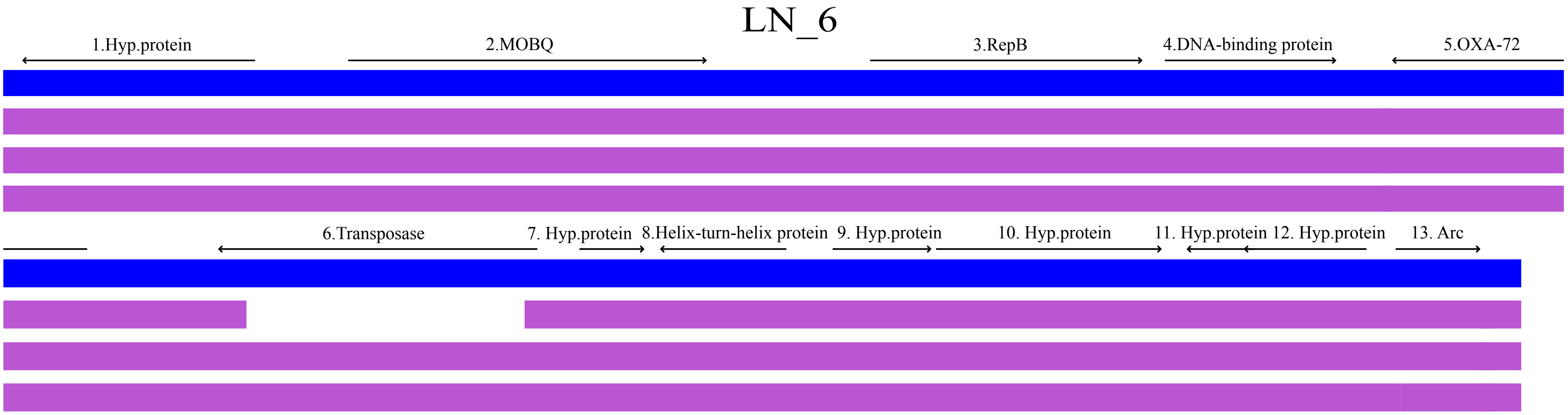
LN_6
1.Hyp.protein
2.MOBQ
3.RepB
4.DNA-binding protein
5.OXA-72
6.Transposase
7. Hyp.protein
8.Helix-turn-helix protein
9. Hyp.protein
10. Hyp.protein
11. Hyp.protein
12. Hyp.protein
13. Arc

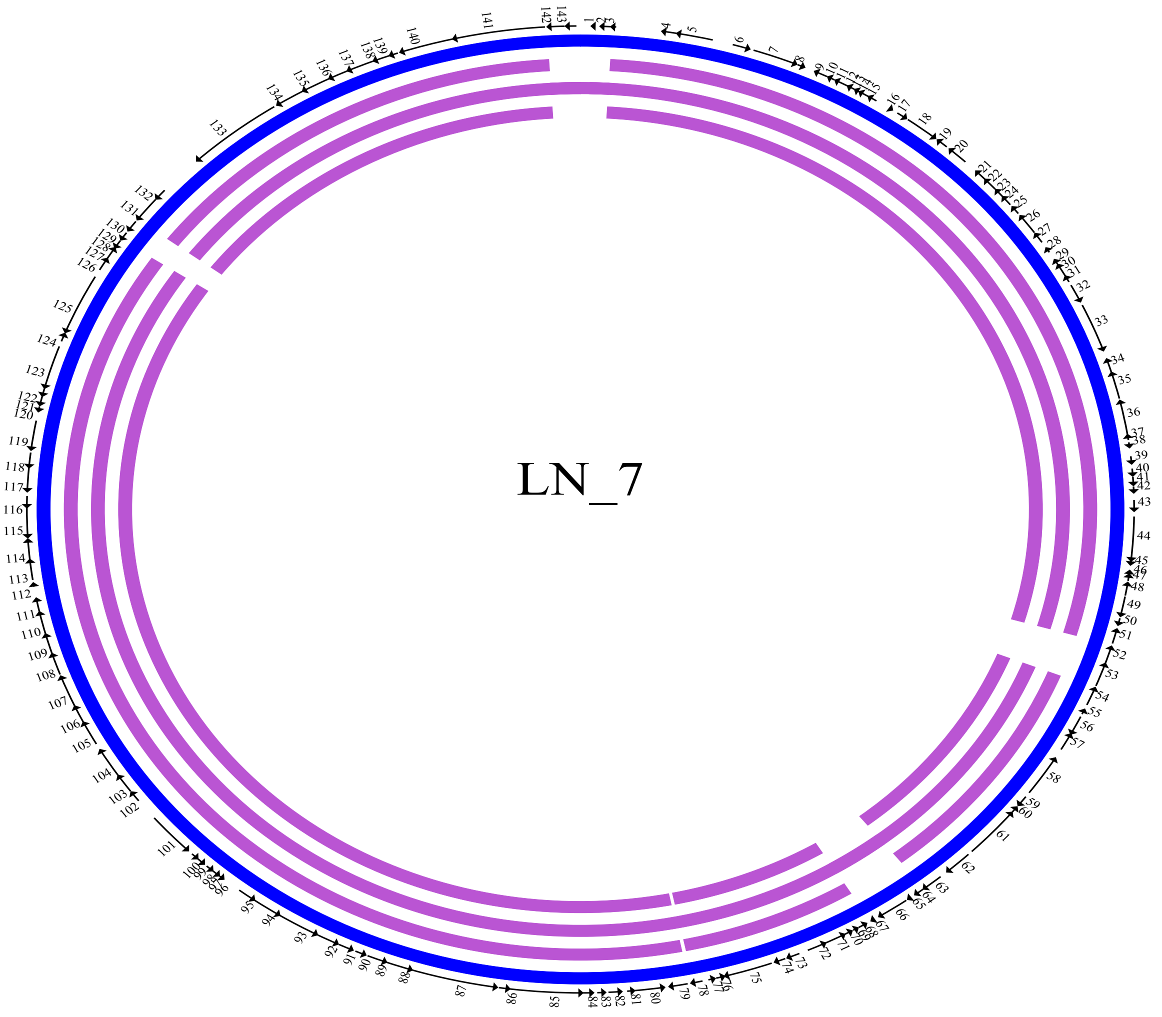
LN_7

**Supplementary Figure 4.** Linear map of members of plasmid lineage LN_6 and circular map of LN_7. LN_6. The representative plasmid of this lineage is pAba10042a (blue bar). Core genes are shown in bold letters. Genes of this plasmid: **1. Hypothetical protein; 2. Relaxase MOBQ; 3. RepB family plasmid replication initiator protein; 4. DNA-binding protein; 5. Carbapenemase class D beta-lactamase OXA-72**; 6. Transposase; **7. Hypothetical protein; 8. Helix-turn-helix domain-containing protein; 9. Hypothetical protein; 10. Hypothetical protein; 11. Hypothetical protein; 12. Hypothetical protein; 13. Arc family DNA-binding protein**. Purple bars: from top to bottom: pAba9201a, pAba5845a, pAba6972a.

LN_7. The representative plasmid of this lineage is pNaval18-131 (blue ring): 1. ISAba25; 2. Transposase; **3. Hypothetical protein; 4. Hypothetical protein; 5. Hypothetical protein; 6. Hypothetical protein; 7. Hypothetical protein 8. Hypothetical protein; 9. Hypothetical protein; 10. Hypothetical protein; 11. Hypothetical protein; 12. Hypothetical protein; 13. Hypothetical protein; 14. Putative lipoprotein; 15. Hypothetical protein; 16. Hypothetical protein; 17. Hypothetical protein; 18. Hypothetical protein; 19. Hypothetical protein; 20. Hypothetical protein; 21. Hypothetical protein; 22. Transcriptional regulator, TetR family; 23. Hypothetical protein; 24. Hypothetical protein; 25. Hypothetical protein; 26. Hypothetical protein; 27. Hypothetical protein; 28. Hypothetical protein; 29. Hypothetical protein; 30. Transcriptional regulator, TetR family; 31. Hypothetical protein; 32. Peptidase, M48 family; 33. GHKL domain protein; 34. Hypothetical protein; 35. Hypothetical protein; 36. Hypothetical protein; 37. Hypothetical protein; 38. Phage integrase, N-terminal SAM domain protein; 39. Site-specific tyrosine recombinase XerC; 40. Hypothetical protein; 41. Hypothetical protein; 42. Hypothetical protein; 43. TnsA endonuclease N-terminal domain protein; 44. Integrase core domain protein; 45. Hypothetical protein; 46. Hypothetical protein; 47. Transposase, IS4-like family protein; 48. Hypothetical protein; 49. Sulfonamide-resistant Sul2; 50. Phosphoglucomutase/phosphomannomutase, alpha/beta/alpha domain I protein; 51. Transposase domain prote**in; 52. IstB-like ATP-binding protein; 53. Integrase core domain protein; **54. Transposase zinc-binding domain protein; 55. Hypothetical protein; 56. Aminoglycoside resistence strB; 57. Aminoglycoside resistence strA; 58. Hypothetical protein; 59. Hypothetical protein; 60. Hypothetical protein; 61. Putative DNA topoisomerase I; 62. Hypothetical protein; 63. Hypothetical protein**; 64. Transposase; 65. IS66 family element, Orf2 domain protein; 66. IS66 family element, transposase; 67. IS66 C-terminal element; **68. Hypothetical protein; 69. Hypothetical protein; 70. PF07308 family protein; 71. Transglycosylase SLT domain protein; 72. Hypothetical protein; 73. Hypothetical protein; 74. Hypothetical protein; 75. Hypothetical protein; 76. Hypothetical protein; 77. Hypothetical protein; 78. Hypothetical protein; 79. Integrase core domain protein; 80. S-(hydroxymethyl)glutathione dehydrogenase; 81. Metal-sensitive transcriptional repressor; 82. Transposase, IS4 family; 83. Hypothetical protein; 84. Hypothetical protein; 85. Hypothetical protein; 86. Hypothetical protein; 87. Conjugal protein TraU; 88. Hypothetical protein; 89. Hypothetical protein; 90. Hypothetical protein; 91. Hypothetical protein; 92. Hypothetical protein; 93. TrbI-like protein; 94. Conjugal protein TraN; 95. Conjugal protein TraM; 96. Hypothetical protein; 97. Hypothetical protein; 98. Hypothetical protein; 99. Hypothetical protein; 100. Hypothetical protein; 101. Hypothetical protein; 102. Putative lipoprotein; 103. Conjugal protein TraI; 104. Type II/IV secretion system domain protein; 105. StbA protein; 106. Hypothetical protein; 107. Hypothetical protein; 108. Hypothetical protein; 109. Hypothetical protein; 110. Hypothetical protein; 111. Hypothetical protein; 112. Hypothetical protein; 113. Partition protein ParA; 114. Partition protein ParB; 115. ImpB/MucB/SamB family protein; 116. Peptidase S24-like protein; 117. Nucleotidyl transferase, PF08843 family; 118. PF13338 domain protein; 119. Hypothetical protein; 120. Hypothetical protein; 121. Hypothetical protein; 122. Hypothetical protein; 123. Peptidase, M23 family; 124. Hypothetical protein; 125. Conjugal protein TraY; 126. Hypothetical protein; 127. Hypothetical protein; 128. Hypothetical protein; 129. IS1008 domain protein**; 130. IS1008 domain protein; **131. Hypothetical protein; 132. Hypothetical protein; 133. Hypothetical protein;**

**134. Conjugal protein TraW; 135. Hypothetical protein; 136. Hypothetical protein; 137. Hypothetical protein; 138. Hypothetical protein; 139. Hypothetical protein; 140. Hypothetical protein; 141. helicase C-like protein**; 142. IS66 C-terminal element; 143. IS66 family element, transposase domain protein. Purple rings: from outside to inside: pOIFC137-122, pOIFC143-128, pOIFC109-122.

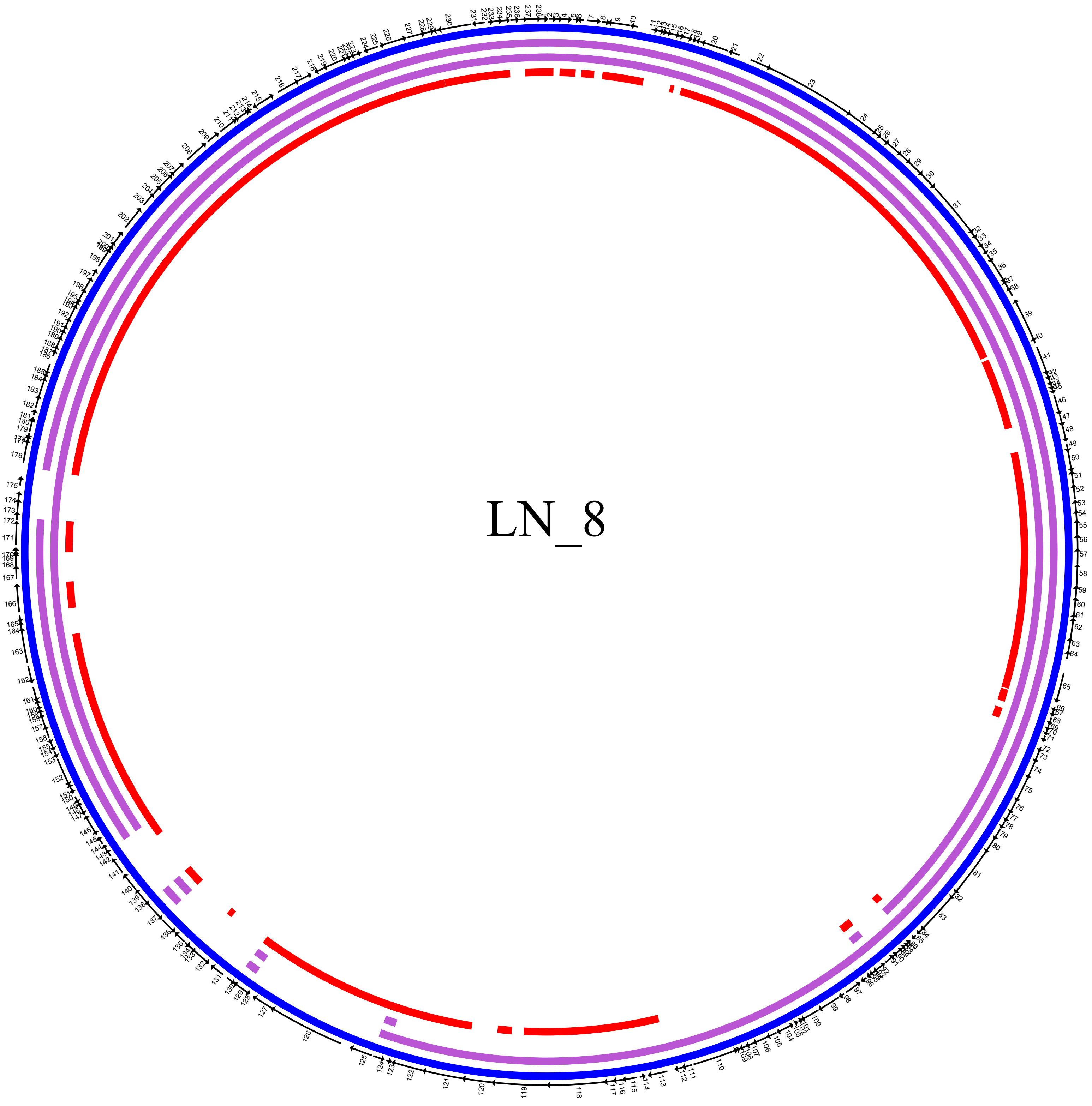
LN_8

**Supplementary Figure 5.** Circular maps of members of plasmid lineage LN_8. The representative plasmid is pB11911 (blue ring). Core genes are shown in bold letters. Genes of this plasmid: **1. Hypothetical protein**; 2. Hypothetical protein; **3. Hypothetical protein**; **4. TetR family transcriptional regulator**; 5. Hypothetical protein; 6. Hypothetical protein; **7. Hypothetical protein**; 8. Hypothetical protein; **9. Hypothetical protein; 10. Hypothetical protein**; 11. Hypothetical protein; 12. Hypothetical protein; 13. Hypothetical protein; 14. Hypothetical protein; 15. Hypothetical protein; **16. Hypothetical protein**; 17. Hypothetical protein; **18. Hypothetical protein; 19. H-NS histone; 20. Hypothetical protein; 21. Hypothetical protein; 22. Hypothetical protein; 23. Helicase; 24. Hypothetical protein; 25. Hypothetical protein; 26. Hypothetical protein; 27. Hypothetical protein; 28. Hypothetical protein; 29. Conjugal protein TraW; 30. Hypothetical protein; 31. Hypothetical protein; 32. Hypothetical protein; 33. Hypothetical protein; 34. Hypothetical protein; 35. Hypothetical protein; 36. Hypothetical protein; 37. Hypothetical protein; 38. Hypothetical protein; 39. Conjugal protein TraY; 40. Hypothetical protein; 41. Peptidase M23; 42. Hypothetical protein; 43. Hypothetical protein; 44. Hypothetical protein; 45. Hypothetical protein; 46. Hypothetical protein**; 47. Hypothetical protein; 48. Nucleotidyl transferase AbiEii/AbiGii toxin family protein; **49. DNA polymerase V; 50. DNA polymerase V subunit UmuC; 51. Partition protein ParB; 52. Partition protein ParA; 53. Hypothetical protein; 54. Hypothetical protein; 55. Hypothetical protein; 56. Hypothetical protein; 57. Hypothetical protein; 58. Hypothetical protein; 59. Hypothetical protein; 60. Hypothetical protein; 61. Hypothetical protein; 62. Hypothetical protein; 63. Conjugal protein TraI; 64. Hypothetical protein; 65. Hypothetical protein; 66. Hypothetical protein; 67. Hypothetical protein; 68. Hypothetical protein**; 69. Hypothetical protein; **70. Hypothetical protein**; 71. Hypothetical protein; 72. Hypothetical protein; 73. Conjugal protein TraM; 74. Conjugal protein TraN; 75. TrbI; 76. Hypothetical protein; 77. Hypothetical protein; 78. Hypothetical protein; 79. Hypothetical protein; 80. Hypothetical protein; 81. Conjugal protein TraU; 82. Hypothetical protein; 83. Hypothetical protein; 84. Hypothetical protein; **85. Hypothetical protein**; 86. Hypothetical protein; 87. Hypothetical protein; 88. Hypothetical protein; 89. Hypothetical protein; 90. Hypothetical protein; 91. Hypothetical protein**; 92. IS6 family transposase; 93. DNA invertase; 94. Hypothetical protein; 95. IstB-like ATP-binding domain protein**; 96. DNA-invertase from lambdoid prophage Rac domain; 97. AraC family transcriptional regulator; 98. Hypothetical protein; 99. Cytochrome P450; 100. Pyridine nucleotide-disulfide oxidoreductase; 101. IS5 family transposase; 102. Hypothetical protein; 103. Hypothetical protein; 104. Metal-dependent hydrolase; 105. TetR family transcriptional regulator; 106. Peptidase; 107. Hypothetical protein; 108. Hypothetical protein; 109. Hypothetical protein; 110. Tn3 family transposase ISAcsp1; 111. Hypothetical protein; 112. HNH endonuclease; 113. Hypothetical protein; 114. Hypothetical protein; 115. WYL domain-containing protein; 116. Hypothetical protein; 117. Hypothetical protein; 118. Hypothetical protein; 119. Class I SAM-dependent DNA methyltransferase; 120. DUF262 domain-containing protein; 121. TIGR02687 family protein; 122. TIGR02688 family protein; 123. Serine/threonine protein phosphatase; **124. IS6 family transposase**; 125. Sodium-independent anion transporter; 126. Hypothetical protein; 127. Hypothetical protein; 128. Helix-turn-helix domain of resolvase; 129. IS6 family transposase IS1007; **130. Oxidoreductase**; 131. LysR family transcriptional regulator; 132. IS3 family transposase; 133. Hypothetical protein; 134. BMP family ABC transporter substrate-binding protein; 135. IS5 family transposase ISAba37; 136. Hypothetical protein; 137. IS4 family transposase; 138. Penicillin synthase; 139. Hypothetical protein; 140. IS3 family transposase; 141. Aromatic ring-hydroxylating dioxygenase subunit; 142. IS5/IS1182 family transposase; 143. Hypothetical protein; **144. IS5/IS1182 family transposase; 145. Metal/formaldehyde-sensitive transcriptional repressor; 146. S-(hydroxymethyl)glutathione dehydrogenase; 147. IS6 family transposase IS1008; 148. DNA invertase; 149. Hypothetical protein; 150. Hypothetical protein; 151. Hypothetical protein; 152. Hypothetical protein; 153. Hypothetical protein; 154. Hypothetical protein; 155. Hypothetical protein; 156. Hypothetical protein; 157. Hypothetical protein; 158. Hypothetical protein; 159. Hypothetical protein; 160. Hypothetical protein; 161. Hypothetical protein; 162. Hypothetical protein**; 163. DNA topoisomerase I; 164. Hypothetical protein; 165. Hypothetical protein; **166. Hypothetical protein**; 167. Aminoglycoside O-phosphotransferase APH(3'')-Ib; 168. Aminoglycoside O-phosphotransferase APH(6)-Id; 169. Hypothetical protein; 170. Hypothetical protein; **171. IS91 family transposase;** 172. Hypothetical protein; 173. Thymidylate synthase; 174.

Dihydrofolate reductase; 175. Deoxynucleoside kinase; **176. IS91 family transposase; 177. Hypothetical protein; 178. Phosphoglucosamine mutase; 179. Sulfonamide-resistant dihydropteroate synthase Sul2; 180. Transcriptional regulator; 181. Hypothetical protein; 182. Partition protein ParA; 183. Partition protein ParB; 184. Hypothetical protein; 185. Type I restriction endonuclease subunit M; 186. Hypothetical protein; 187. Hypothetical protein; 188. Thymidylate kinase; 189. Hypothetical protein; 190. Hypothetical protein; 191. Hypothetical protein; 192. DNA methyltransferase; 193. hypothetical protein; 194. Hypothetical protein; 195. IS6 family transposase IS15DIV; 196. Class 1 integron integrase IntI1; 197. NAD(+)--rifampin ADP-ribosyltransferase Arr-2; 198. Chloramphenicol efflux MFS transporter CmlA5; 199. Hypothetical protein; 200. Quaternary ammonium efflux SMR transporter QacE delta 1; 201. Sulfonamide-resistant Sul1; 202. IS91 family transposase; 203. Class A extended-spectrum beta-lactamase PER-7; 204. Glutathione S-transferase; 205. Multidrug ABC transporter ATP-binding protein; 206. QacE family quaternary ammonium efflux SMR transporter; 207. Sulfonamide-resistant Sul1; 208. IS91 family transposase; 209. IS5 family transposase; 210. IS4 family transposase; 211. Hypothetical protein; 212. ArmA family 16S rRNA (guanine(1405)-N(7))-methyltransferase; 213. Hypothetical protein; 214. Hypothetical protein; 215. IS4 family transposase ISEc29; 216. ABC-F type ribosomal protection protein Msr(E); 217. Mph(E) family macrolide 2'-phosphotransferase; 218. Hypothetical protein; 219. RepB family plasmid replication initiator protein; 220. IS6 family transposase IS15DIV; 221. Hypothetical protein; 222. Hypothetical protein; 223. Hypothetical protein; 224. Tyrosine recombinase; 225. Hypothetical protein; 226. Hypothetical protein; 227. Hypothetical protein; 228. Hypothetical protein; 229. Hypothetical protein; 230. DNA mismatch repair protein; 231. Hypothetical protein; 232. Hypothetical protein; 233. TetR family transcriptional regulator; 234. Hypothetical protein; 235.** Hypothetical protein; 236. Hypothetical protein; **237. Hypothetical protein; 238. Hypothetical protein**. Purple rings: from outside to inside: pIOMTU433, pAB04-1. Additional orphan plasmid is in red ring: pHWBA8_1.

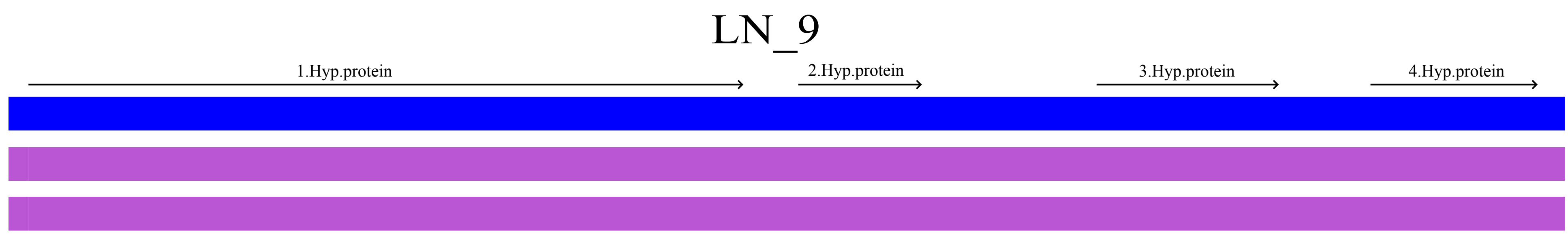
LN_9
1.Hyp.protein
2.Hyp.protein
3.Hyp.protein
4.Hyp.protein

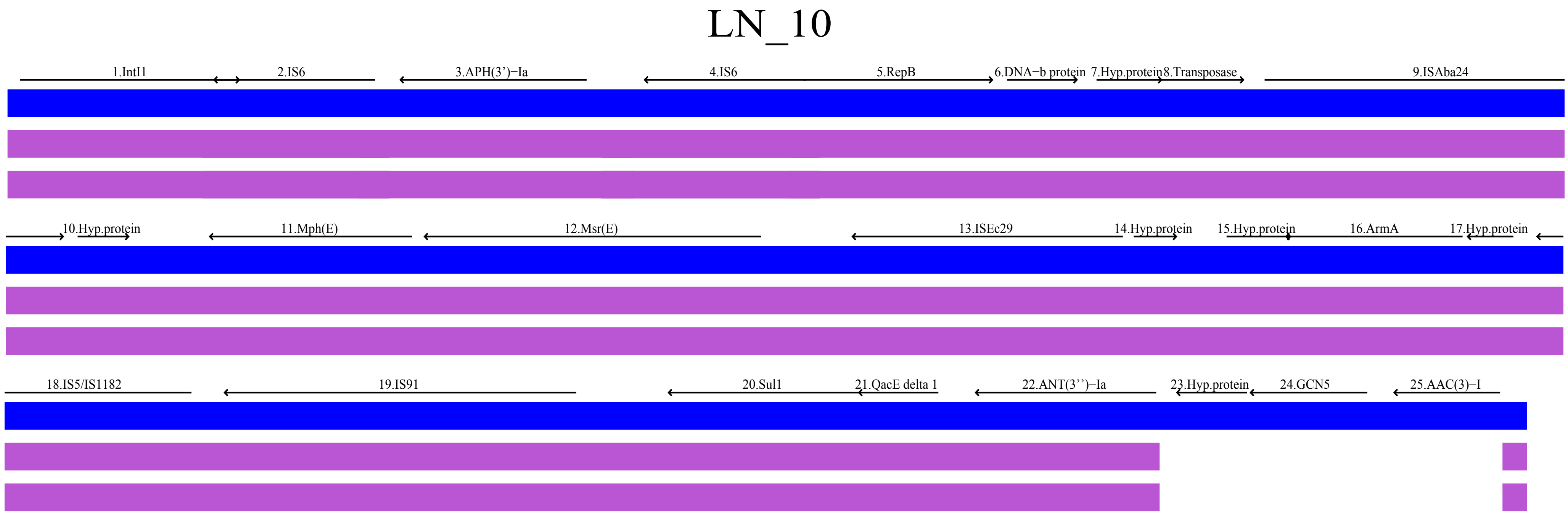
LN_10
1.IntI1
2.IS6
3.APH(3')−Ia
4.IS6
5.RepB
6.DNA−b protein
7.Hyp.protein
8.Transposase
9.ISAba24
10.Hyp.protein
11.Mph(E)
12.Msr(E)
13.ISEc29
14.Hyp.protein
15.Hyp.protein
16.ArmA
17.Hyp.protein
18.IS5/IS1182
19.IS91
20.Sul1
21.QacE delta 1
22.ANT(3'')−Ia
23.Hyp.protein
24.GCN5
25.AAC(3)−I

**Supplementary Figure 6.** Linear maps of members of plasmid lineages LN_9 and LN_10. LN_9. The representative plasmid is p4ABAYE (blue bar). Core are shown in bold letters. Genes of this plasmid: **1. Hypothetical protein; 2. Hypothetical protein; 3. Hypothetical protein; 4. Hypothetical protein.** Purple bars: from top to bottom: pMRSN58-2.7, pA85-1.

LN_10. pMDR-ZJ06 (representative plasmid) blue bar and its genes are**: 1. Class 1 integron integrase Intl1; 2. IS6 family transposase; 3. Aminoglycoside O-phosphotransferase APH(3')-Ia; 4. IS6 family transposase; 5. RepB family plasmid replication initiator protein; 6. DNA-binding protein; 7. Hypothetical protein; 8. Transposase; 9. IS66 family transposase ISAba24; 10. Hypothetical protein; 11. Mph(E) family macrolide 2'-phosphotransferase; 12. ABC-F type ribosomal protection protein Msr(E) ; 13. IS4 family transposase ISEc29; 14. Hypothetical protein; 15. Hypothetical protein; 16. ArmA family 16S rRNA (guanine(1405)-N(7))-methyltransferase; 17. Hypothetical protein; 18. IS5/IS1182 family transposase; 19. IS91 family transposase; 20. Sulfonamide-resistant dihydropteroate synthase Sul1; 21. Quaternary ammonium compound efflux SMR transporter QacE delta; 22. ANT(3")-Ia family aminoglycoside nucleotidyltransferase AadA1C**; 23. Hypothetical protein; 24. GCN5 family acetyltransferase; 25. AAC(3)-I family aminoglycoside 3-N-acetyltransferase. Purple bars: from top to bottom: p2BJAB07104, p3BJAB0868.

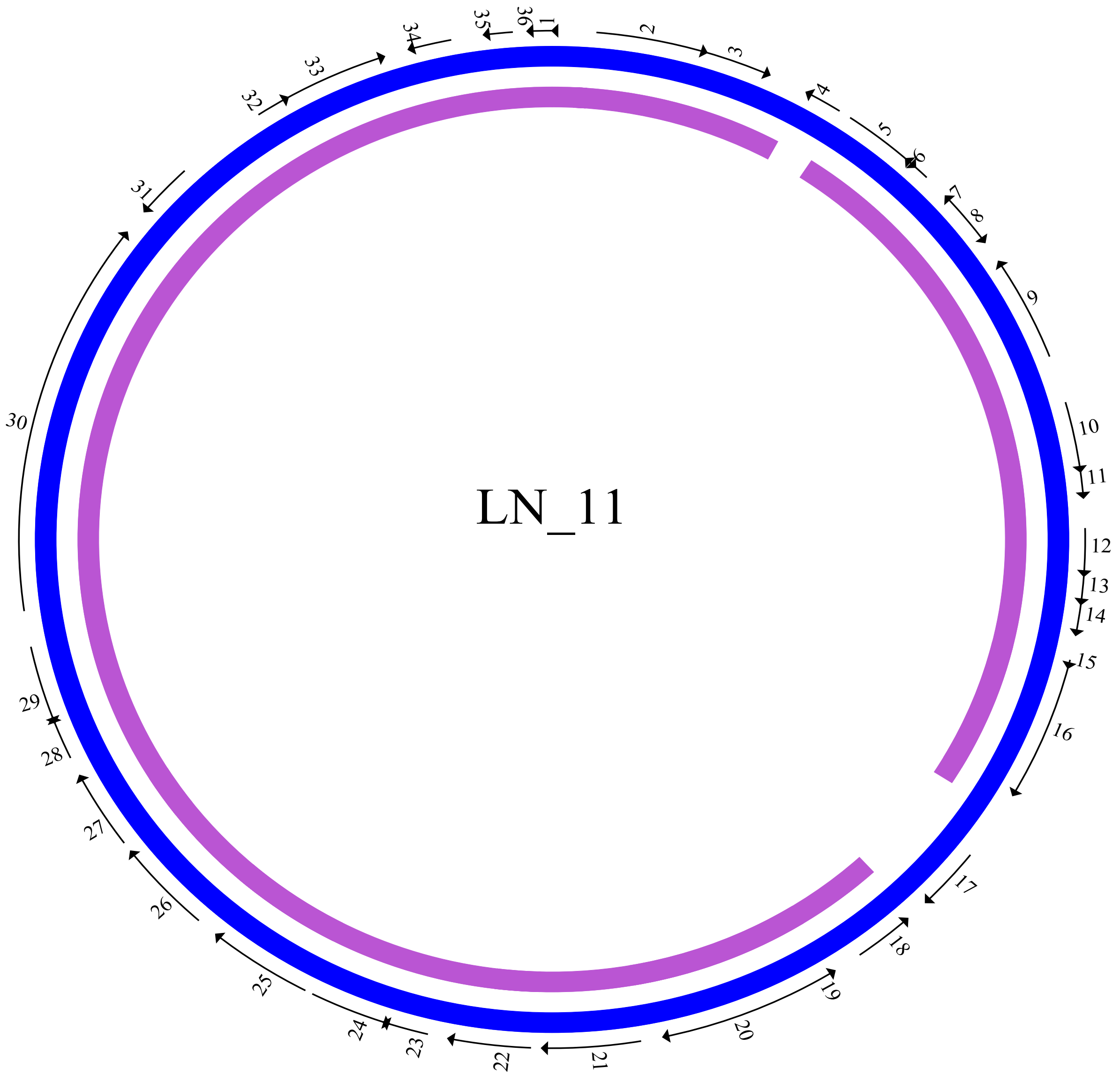

LN_11
1
2
3
4
5
6
7
8
9
10
11
12
13
14
15
16
17
18
19
20
21
22
23
24
25
26
27
28
29
30
31
32
33
34
35
36


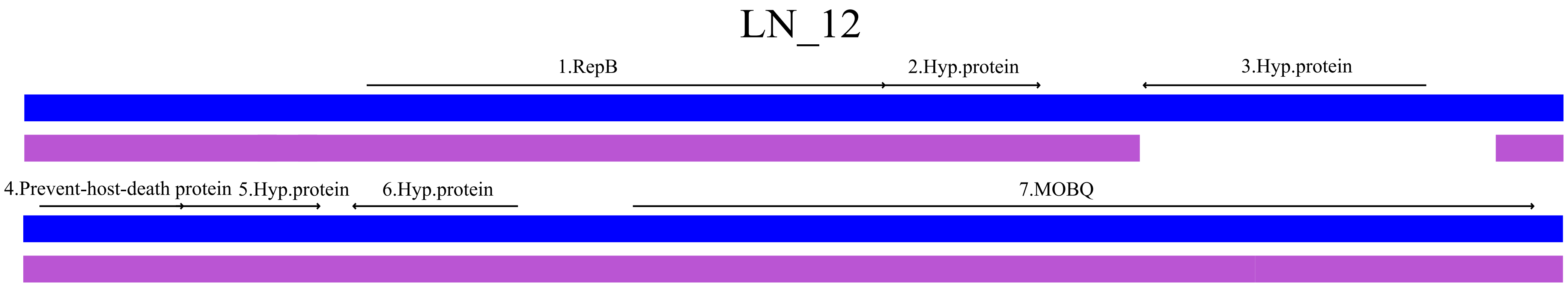

LN_12
1.RepB
2.Hyp.protein
3.Hyp.protein
4.Prevent-host-death protein
5.Hyp.protein
6.Hyp.protein
7.MOBQ

**Supplementary Figure 7.** Cicular map of members of plasmid lineage LN_11 and linear map of LN_12. LN_11. Representative plasmid pACICU1 (blue ring) and its genes are**: 1. Hypothetical protein; 2. RepB family plasmid replication initiator protein; 3. DNA-binding protein**; 4. Hypothetical protein**; 5. IS6 family transposase; 6. Hypothetical protein; 7. Hypothetical protein; 8. Hypothetical protein; 9. RepB family plasmid replication initiator protein; 10. Partition protein ParA; 11. Hypothetical protein; 12. Hypothetical protein; 13. Antitoxin RelB; 14. Toxin RelE; 15. Permease; 16. IS3 family transposase**; 17. IS15DIV; **18. Hypothetical protein; 19. Hypothetical protein; 20. IS3 family transposase; 21. Carbapenem-hydrolyzing class D beta-lactamase OXA-58; 22. IS1 family transposase; 23. AraC family transcriptional regulator; 24. IS6 family transposase; 25. IS3 family transposase; 26. Carbapenem-hydrolyzing class D beta-lactamase OXA-58; 27. IS1 family transposase; 28. AraC family transcriptional regulator; 29. IS6 family transposase; 30. Hypothetical protein; 31. Hypothetical protein; 32. Transcriptional regulator; 33. Reverse transcriptase; 34. Hypothetical protein; 35. Hypothetical protein; 36. Hypothetical protein**. Purple ring: p2ABST2.

LN_12. The representative plasmid is p1ABAYE (blue bar). Genes of this plasmid: **1. RepB family plasmid replication initiator protein; 2. Hypothetical protein**; 3. Hypothetical protein**; 4. Prevent-host-death family protein; 5. Hypothetical protein; 6. Hypothetical protein7. Relaxase MOBQ**. Purple bar: p1M3AC14-8.

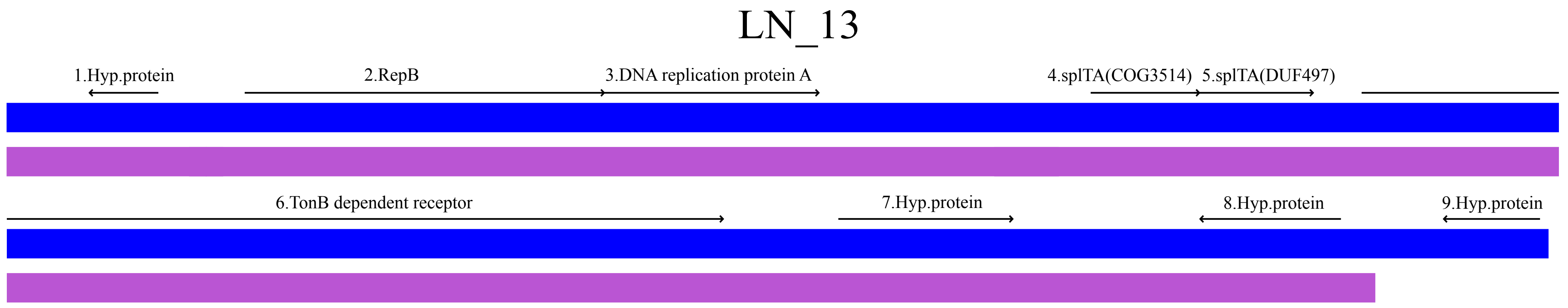
LN_13
1.Hyp.protein
2.RepB
3.DNA replication protein A
4.splTA(COG3514)
5.splTA(DUF497)
6.TonB dependent receptor
7.Hyp.protein
8.Hyp.protein
9.Hyp.protein

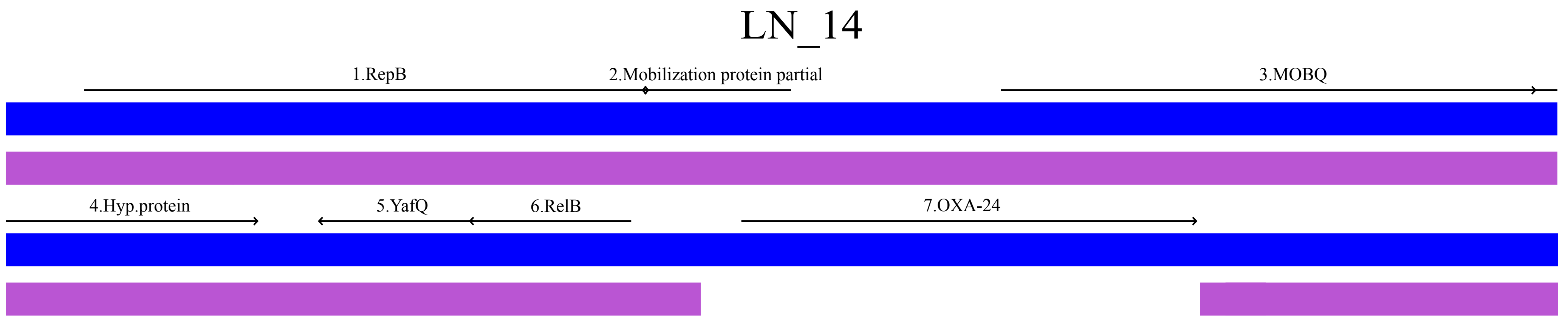
LN_14
1.RepB
2.Mobilization protein partial
3.MOBQ
4.Hyp.protein
5.YafQ
6.RelB
7.OXA-24

**Supplementary Figure 8.** Linear maps of members of plasmid lineages LN_13 and LN_14. LN_13. The representative plasmid is pCS01C. (blue bar) and its genes are: **1. Hypothetical protein; 2. Initiator RepB protein; 3. DNA-binding protein; 4. Toxin-Antitoxin system splTA (COG3514); 5. Toxin-Antitoxin system splTA (DUF497); 6. TonB dependent receptor; 7. Hypothetical protein; 8. Hypothetical protein; 9. Hypothetical protein.** Purple bar: pCR17C.

LN_14. The representative plasmid of this lineage is pABUH2a-5.6 (blue bar) and its genes are: **1. Replication protein RepB; 2. Mobilization protein partial; 3. Mobilization protein MOBQ4. Hypothetical protein; 5. Toxin YafQ; 6. Antitoxin RelB**; 7. Carbapenem-hydrolyzing class D beta-lactamase OXA-24. Purple bar: pABUH2b-5.4

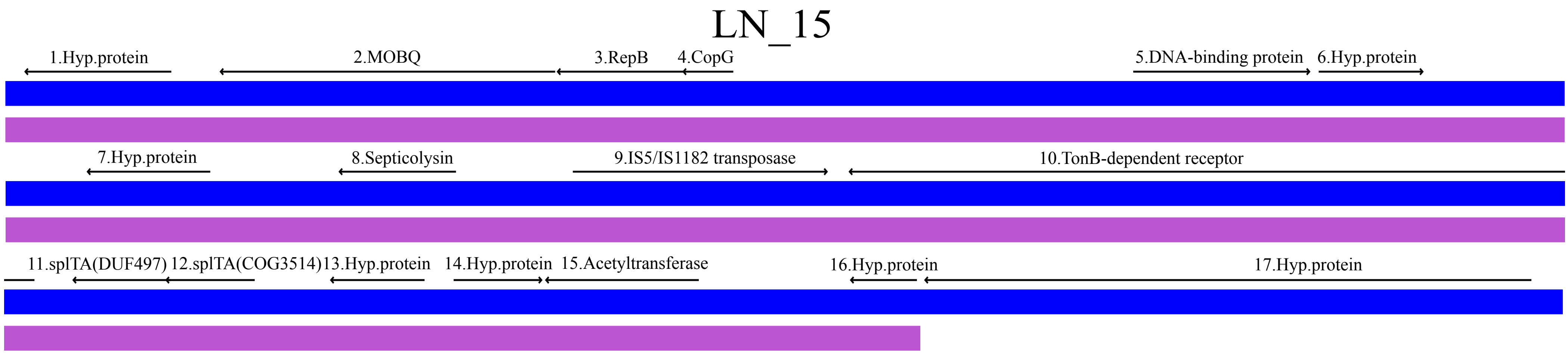
LN_15
1.Hyp.protein
2.MOBQ
3.RepB
4.CopG
5.DNA-binding protein
6.Hyp.protein
7.Hyp.protein
8.Septicolysin
9.IS5/IS1182 transposase
10.TonB-dependent receptor
11.splTA(DUF497)
12.splTA(COG3514)
13.Hyp.protein
14.Hyp.protein
15.Acetyltransferase
16.Hyp.protein
17.Hyp.protein

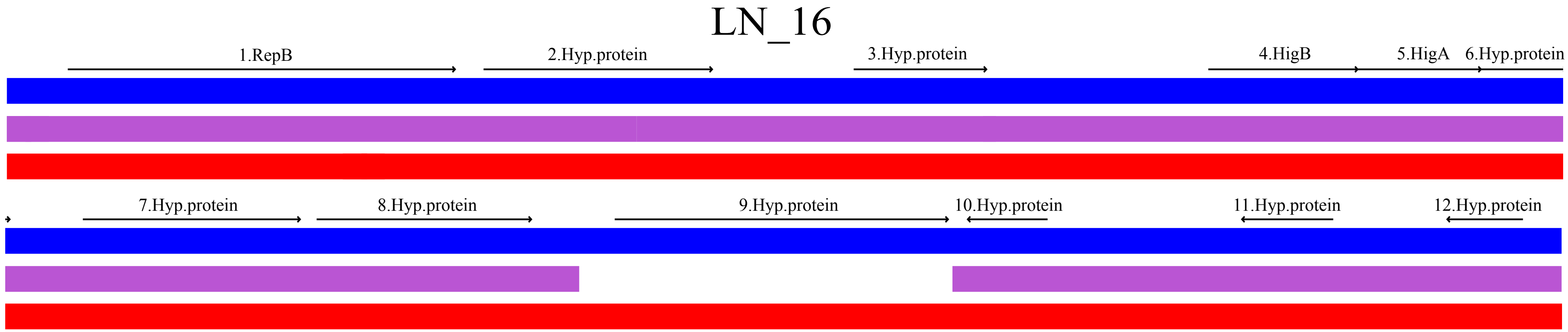
LN_16
1.RepB
2.Hyp.protein
3.Hyp.protein
4.HigB
5.HigA
6.Hyp.protein
7.Hyp.protein
8.Hyp.protein
9.Hyp.protein
10.Hyp.protein
11.Hyp.protein
12.Hyp.protein

**Supplementary Figure 9.** Linear maps of members of plasmid lineages LN_15 and LN_16. LN_15. Blue bar corresponds to the representative plasmid pAba810CPa and its genes are: **1. Hypothetical protein; 2. Relaxase MOBQ; 3. RepB family plasmid replication initiator protein4. CopG family transcriptional regulator; 5. DNA-binding protein; 6. Hypothetical protein; 7. Hypothetical protein; 8. Septicolysin; 9. IS5/IS1182 family transposase; 10. TonB-dependent receptor; 11. Toxin-Antitoxin system spITA (DUF497); 12. Toxin-Antitoxin system spITA (COG3514); 13. Hypothetical protein; 14. Hypothetical protein; 15. Acetyltransferase; 16. Hypothetical protein**; 17. Hypothetical protein. Purple bar: plasmid pAba11510a.

LN_16. The representative plasmid is pD1279779 (blue bar) The genes core are in bold letters. Genes of this plasmid:**1. RepB family plasmid replication initiator protein; 2. Hypothetical protein; 3. Hypothetical protein; 4. Toxin HigB; 5. Antitoxin HigA; 6. Hypothetical protein 7. Hypothetical protein; 8. Hypothetical protein**; 9. Hypothetical protein; **10. Hypothetical protein; 11. Hypothetical protein; 12. Hypothetical protein**. Purple bar: pAba10324b. Additional orphan plasmid is in red bar: pABLAC1

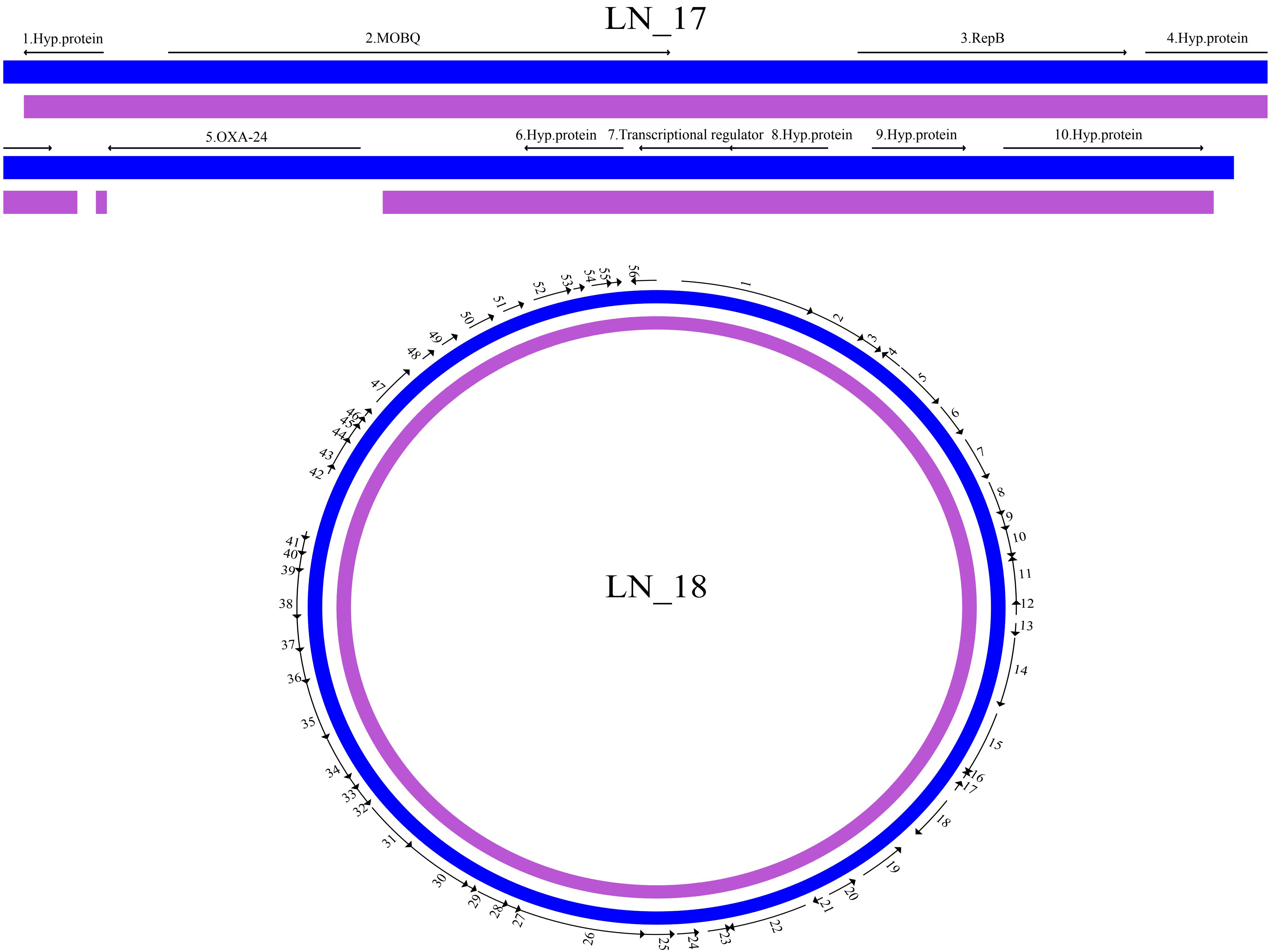
LN_17
1.Hyp.protein
2.MOBQ
3.RepB
4.Hyp.protein
5.OXA-24
6.Hyp.protein
7.Transcriptional regulator
8.Hyp.protein
9.Hyp.protein
10.Hyp.protein
LN_18

**Supplementary Figure 10.** Linear map of members of plasmid lineage LN_17 and circular map of LN_18. LN_17. The representative plasmid is pABUH3a-8.2 (blue bar) and its genes are: **1. Hypothetical protein; 2. Mobilization protein MOBQ; 3. RepB family plasmid replication initiator protein; 4. Hypothetical protein**; 5. Carbapenem-hydrolyzing class D beta-lactamase OXA-24; **6. Hypothetical protein; 7. Transcriptional regulator; 8. Hypothetical protein; 9. Hypothetical protein 10. Hypothetical protein**. Purple bar: pABUH3b-7.8.

LN_18. The representative plasmid is pAbNDM-1 (blue ring) and its genes are: **1. Relaxase MOBQ; 2. Hypothetical protein; 3. Hypothetical protein; 4. Hypothetical protein; 5. IS3 family transposase; 6. APH(3')-VI family aminoglycoside O-phosphotransferase; 7. IS30 family transposase; 8. Subclass B1 metallo-beta-lactamase NDM-1; 9. Bleomycin binding protein Ble-MBL; 10. Phosphoribosylanthranilate isomerase; 11. Twin-arginine translocation (TAT) pathway signal sequence domain protein; 12. Divalent-cation tolerance protein A; 13. Molecular chaperone GroES; 14. Molecular chaperone GroEL; 15. IS91 family transposase; 16. Hypothetical protein; 17. Hypothetical protein; 18. IS30 family transposase; 19. IS30 family transposase; 20. Hypothetical protein; 21. Hypothetical protein; 22. Hypothetical protein; 23. Partition protein ParB; 24. Hypothetical protein; 25. Hypothetical protein; 26. VirB4; 27. Hypothetical protein; 28. Transglycosylase; 29. Hypothetical protein; 30. VirD4; 31. Transglycosylase; 32. Hypothetical protein; 33. Hypothetical protein; 34. P-type DNA transfer ATPase VirB11; 35. Hypothetical protein; 36. P-typeonjugative transfer protein VirB9; 37. Hypothetical protein; 38. Hypothetical protein; 39. Hypothetical protein; 40. Hypothetical protein; 41. Hypothetical protein; 42. Hypothetical protein; 43. Hypothetical protein; 44. Hypothetical protein; 45. Hypothetical protein; 46. Hypothetical protein; 47. Hypothetical protein; 48. Hypothetical protein; 49. Hypothetical protein; 50. Hypothetical protein; 51. Hypothetical protein; 52. DUFF3560 Domain-containing protein; 53. Hypothetical protein; 54. Hypothetical protein; 55. Hypothetical protein; 56. Hypothetical protein**. Purple ring: p6200-47.274kb.

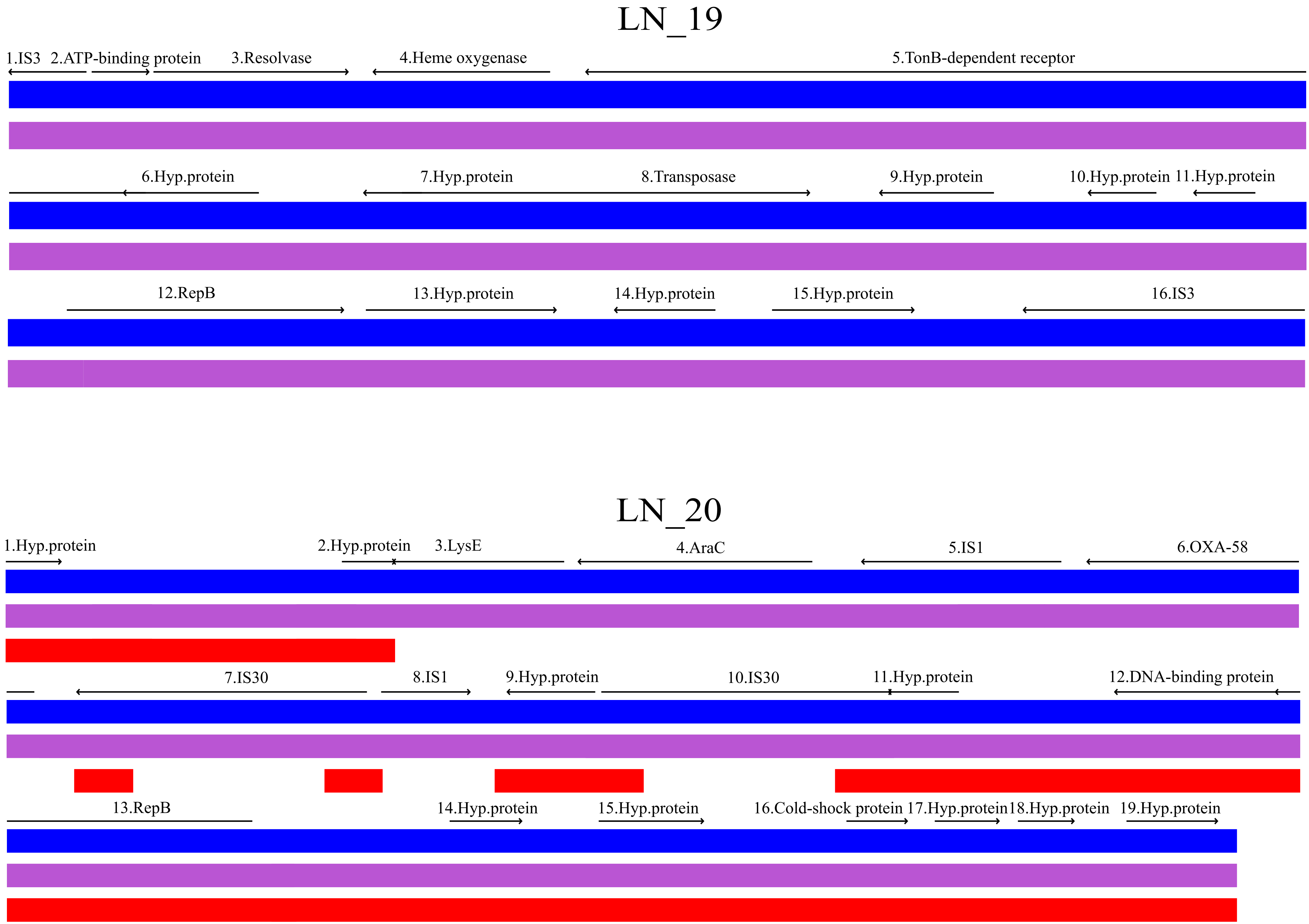
LN_19
1.IS3
2.ATP-binding protein
3.Resolvase
4.Heme oxygenase
5.TonB-dependent receptor
6.Hyp.protein
7.Hyp.protein
8.Transposase
9.Hyp.protein
10.Hyp.protein
11.Hyp.protein
12.RepB
13.Hyp.protein
14.Hyp.protein
15.Hyp.protein
16.IS3
LN_20
1.Hyp.protein
2.Hyp.protein
3.LysE
4.AraC
5.IS1
6.OXA-58
7.IS30
8.IS1
9.Hyp.protein
10.IS30
11.Hyp.protein
12.DNA-binding protein
13.RepB
14.Hyp.protein
15.Hyp.protein
16.Cold-shock protein
17.Hyp.protein
18.Hyp.protein
19.Hyp.protein

**Supplementary Figure 11.** Linear maps of members of plasmid lineages LN_19 and LN_20. LN_19. The representative plasmid is pNaval17-13 (blue bar). The genes core are in bold letters. Genes of this plasmid: **1. IS3 family transposase; 2. ATP-binding protein; 3. Resolvase; 4. Heme oxigenase; 5. TonB-dependent receptor; 6. Hypothetical protein; 7. Hypothetical protein; 8. Transposase; 9. Hypothetical protein; 10. Hypothetical protein; 11. Hypothetical protein; 12. RepB family plasmid replication initiator protein; 13. Hypothetical protein; 14. Hypothetical protein; 15. Hypothetical protein; 16. IS3 family transposase**. Purple bar: pNaval81-13.

LN_20. The representative plasmid is pAba7847a (blue bar) and its genes are: **1. Hypothetical protein; 2. Hypothetical protein**; 3. LysE family translocator; 4. AraC family transcriptional regulator; 5. IS1 family transposase; 6. Carbapenem-hydrolyzing class D beta-lactamase OXA-58; 7. IS30 family transposase; 8. IS1 family transposase; **9. Hypothetical protein**; 10. IS30 family transposase; **11. Hypothetical protein; 12. DNA-binding protein; 13. RepB family plasmid replication initiator protein; 14. Hypothetical protein; 15. Hypothetical protein; 16. Cold-shock protein; 17. Hypothetical protein; 18. Hypothetical protein; 19. Hypothetical protein.** Purple bar: pAba3207a. Additional orphan plasmid is in red bar: pAba7835a.

# LN_21

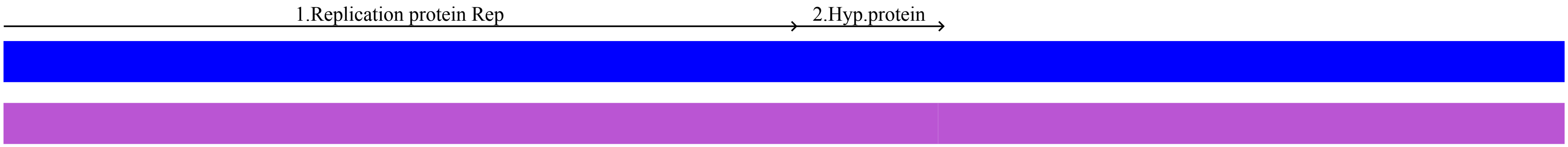

**Supplementary Figure 12.** Linear map of members of plasmid lineage LN_21. The representative plasmid is pA85-1a (blue bar) and its genes are: **1. Replication protein Rep; 2. Hypothetical protein**. Purple bar: pMRSN7339-2.3.

Tree scale: 0.1

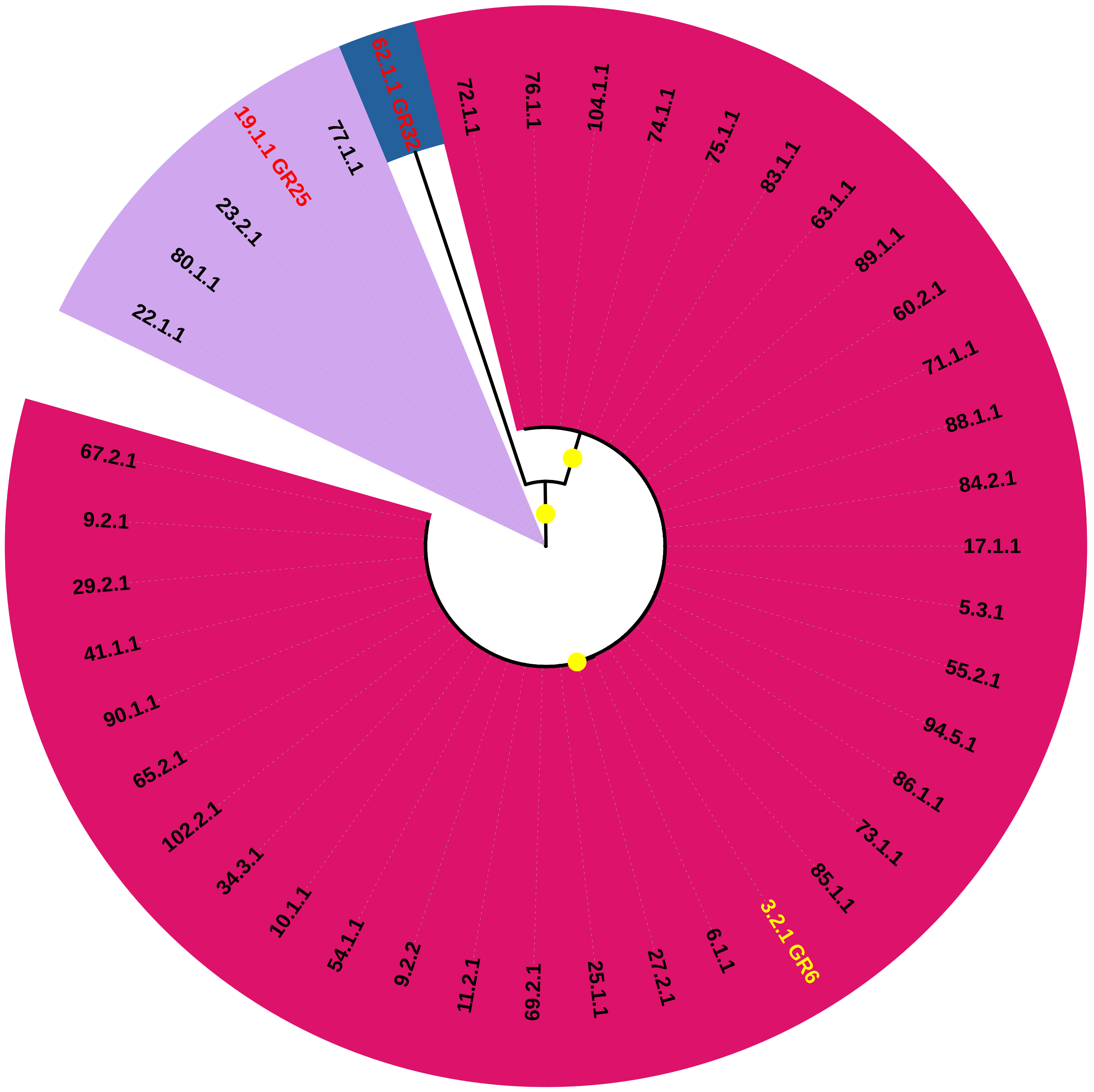

**Supplementary_Figure 13. Phylogenetic tree of genes encoding replicase proteins belonging to the *replicase*-PriCT family.** In this figure we are using the replicase_ID numbers listed in Supplementary Table_1. Each color embrace members of one clade. Names with yellow letters indicate the reference genes used by Bertini and coworkers (2010) to construct GR homology groups. Names with yellow letters show the reference genes used by us to construct the new GR homology groups. Bootstrap values higher than 70% are marked in the figure with yellow circles

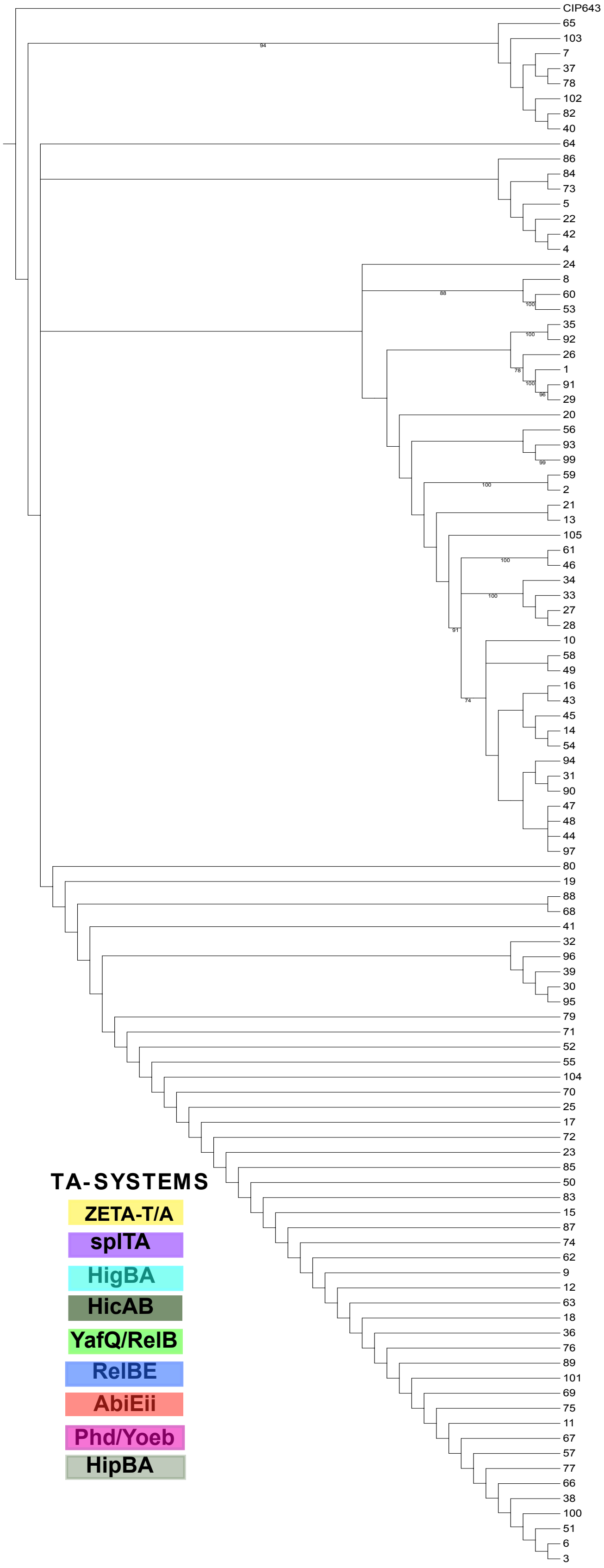

# LINEAGES

| | STRAIN | p1(ID) | p2(ID) | p3(ID) | p4(ID) | p5(ID) | p6(ID) |
|---|---|---|---|---|---|---|---|
| | CIP643 | | | | | | |
| 65 | 3207 | LN_20 (65.1) | LN_1 (65.2) | | | | |
| 103 | 810CP | LN_15 (103.1) | Orph (103.2) | | | | |
| 7 | 9201 | LN_6 (7.1) | | | | | |
| 37 | UH7607 | LN_17 (37.1) | LN_14 (37.2) | | | | |
| 78 | AF-401 | Orph (78.1) | | | | | |
| 102 | 7835 | LN_20 (102.1) | LN_1 (102.2) | | | | |
| 82 | 11510 | LN_15 (82.1) | | | | | |
| 40 | UH19608 | LN_14 (40.1) | LN_17 (40.2) | | | | |
| 64 | ab736 | Orph (64.1) | | | | | |
| 86 | JBA13 | LN_1 (86.1) | Orph (86..2) | | | | |
| 84 | SSA12 | LN_2 (84.1) | LN_1 (84.2) | | | | |
| 73 | KAB04 | LN_1 (73.1) | | | | | |
| 5 | AC30 | LN_2 (5.1) | Orph (5.2) | LN_1 (5.3) | | | |
| 22 | BJAB07104 | LN_5 (22.1) | LN_10 (22.2) | | | | |
| 42 | UH10707 | Orph (42.1) | | | | | |
| 4 | AC12 | LN_2 (4.1) | | | | | |
| 24 | BJAB0715 | Orph (24.1) | | | | | |
| 8 | 10324 | Orph (8.1) | LN_16 (8.2) | LN_3B (8.3) | | | |
| 60 | Ab04-mff | LN_8 (60.1) | LN_1 (60.2) | | | | |
| 53 | LAC-4 | Orph (53.1) | LN_4 (53.2) | | | | |
| 35 | OMTU433 | LN_8 (35.1) | | | | | |
| 92 | B11911 | LN_8 (92.1) | | | | | |
| 26 | OIFC032 | Orph (26.1) | Orph (26.2) | | | | |
| 1 | Naval-18 | LN_4 (1.1) | LN_1 (1.2) | Orph (1.3) | LN_7 (1.4) | Orph (1.5) | Orph (1.6) |
| 91 | HWBA8 | LN_8 (91.1) | | | | | |
| 29 | OIFC143 | Orph (29.1) | LN_1 (29.2) | Orph (29.3) | LN_7 (29.4) | | |
| 20 | ZW85-1 | LN_18 (20.1) | LN_3B (20.2) | | | | |
| 56 | 6200 | LN_3A (56.1) | LN_18 (56.2) | Orph (56.3) | | | |
| 93 | B8300 | Orph (93.1) | | | | | |
| 99 | R2091 | Orph (99.1) | | | | | |
| 59 | ATCC 17978-mff | LN_2 (59.1) | | | | | |
| 2 | ATCC 17978 | Orph (2.1) | Orph (2.2) | | | | |
| 21 | D1279779 | LN_16 (21.1) | | | | | |
| 13 | SDF | Orph (13.1) | Orph (13.2) | Orph (13.3) | | | |
| 105 | 3909 | LN_2 (105.1) | | | | | |
| 61 | D36 | Orph (61.1) | LN_4 (61.2) | Orph (61.3) | Orph (61.4) | | |
| 46 | MRSN 3527 | LN_4 (46.1) | | | | | |
| 34 | IS-123 | Orph (34.1) | Orph (34.2) | LN_1 (34.3) | | | |
| 33 | OIFC109 | LN_7 (33.1) | | | | | |
| 27 | Naval-81 | Orph (27.1) | LN_1 (27.2) | LN_19 (27.3) | | | |
| 28 | OIFC137 | LN_7 (28.1) | | | | | |
| 10 | 9102 | LN_1 (10.1) | | | | | |
| 58 | A1 | LN_2 (58.1) | | | | | |
| 49 | MEX11594 | LN_2 (49.1) | Orph (49.2) | | | | |
| 16 | AB0057 | LN_2 (16.1) | | | | | |
| 43 | MRSN 7339 | LN_21 (43.1) | LN_2 (43.2) | | | | |
| 45 | MRSN 58 | LN_9 (45.1) | LN_2 (45.2) | | | | |
| 14 | AYE | LN_12 (14.1) | LN_2 (14.2) | LN_9 (14.3) | Orph (14.4) | | |
| 54 | AB5075-UW | LN_1 (54.1) | LN_2 (54.2) | Orph (54.3) | | | |
| 94 | A85 | LN_9 (94.1) | LN_21 (94.2) | Orph (94.3) | LN_2 (94.4) | LN_1 (94.5) | |
| 31 | Canada BC-5 | LN_2 (31.1) | | | | | |
| 90 | USA15 | LN_1 (90.1) | | | | | |
| 47 | MRSN 3942 | LN_4 (47.1) | | | | | |
| 48 | MRSN 3405 | LN_4 (48.1) | | | | | |
| 44 | MRSN 4106 | LN_4 (44.1) | | | | | |
| 97 | AB5075 | LN_2 (97.1) | | | | | |
| 80 | XDR-BJ83 | LN_5 (80.1) | | | | | |
| 19 | MDR-TJ | LN_5 (19.1) | LN_3A (19.2) | | | | |
| 88 | 15A34 | LN_1 (88.1) | | | | | |
| 68 | CMC-CR-MDR-Ab59 | LN_3A (68.1) | | | | | |
| 41 | UH9907 | LN_1 (41.1) | | | | | |
| 32 | Naval-17 | LN_19 (32.1) | | | | | |
| 96 | CR17 | LN_1 (96.1) | LN_1 (96.2) | LN_13 (96.3) | | | |
| 39 | UH2107 | LN_3A (39.1) | | | | | |
| 30 | OIFC189 | LN_3A (30.1) | | | | | |
| 95 | CS01 | LN_1 (95.1) | LN_1 (95.2) | LN_13 (95.3) | | | |
| 79 | AF-673 | LN_3A (79.1) | | | | | |
| 71 | KAB02 | LN_1 (71.1) | | | | | |
| 52 | PKAB07 | LN_2 (52.1) | | | | | |
| 55 | AC29 | LN_2 (55.1) | LN_1 (55.2) | | | | |
| 104 | KAB07 | LN_1 (104.1) | | | | | |
| 70 | KAB01 | LN_1 (70.1) | | | | | |
| 25 | 3990 | LN_1 (25.1) | LN_11 (25.2) | | | | |
| 17 | 1656-2 | LN_1 (17.1) | Orph (17.2) | | | | |
| 72 | KAB03 | LN_1 (72.1) | | | | | |
| 23 | BJAB0868 | LN_2 (23.1) | LN_5 (23.2) | LN_10 (23.3) | | | |
| 85 | SSMA17 | LN_1 (85.1) | | | | | |
| 50 | AB210M | LN_2 (50.1) | | | | | |
| 83 | 15A5 | LN_1 (83.1) | LN_2 (83.2) | | | | |
| 15 | ACICU | LN_11 (15.1) | LN_1 (15.2) | | | | |
| 87 | CBA7 | LN_3A (87.1) | | | | | |
| 74 | KAB05 | LN_1 (74.1) | | | | | |
| 62 | KBN10P02143 | Orph (62.1) | | | | | |
| 9 | 10042 | LN_6 (9.1) | LN_1 (9.2) | | | | |
| 12 | 5845 | LN_6 (12.1) | | | | | |
| 63 | YU-R612 | LN_1 (63.1) | LN_2 (63.2) | | | | |
| 18 | MDR-ZJ06 | LN_10 (18.1) | | | | | |
| 36 | UH9707 | LN_2 (36.1) | | | | | |
| 76 | KAB08 | LN_1 (76.1) | | | | | |
| 89 | USA2 | LN_1 (89.1) | | | | | |
| 101 | 6972 | LN_6 (101.1) | | | | | |
| 69 | CMC-CR-MDR-Ab66 | LN_3A (69.1) | LN_1 (69.2) | | | | |
| 75 | KAB06 | LN_1 (75.1) | | | | | |
| 11 | 7847 | LN_20 (11.1) | LN_1 (11.2) | | | | |
| 67 | CMC-CR-MDR-Ab4 | LN_3A (67.1) | LN_1 (67.2) | | | | |
| 57 | XH386 | LN_3A (57.1) | | | | | |
| 77 | HRAB-85 | LN_5 (77.1) | | | | | |
| 66 | OrphAB01 | LN_3A (66.1) | Orph (66.2) | LN_2 (66.3) | | | |
| 38 | UH7007 | Orph (38.1) | | | | | |
| 100 | BAL062 | Orph (100.1) | | | | | |
| 51 | M3AC14-8 | LN_12 (51.1) | Orph (51.2) | | | | |
| 6 | AB34299 | LN_1 (6.1) | LN_2 (6.2) | | | | |
| 3 | TCDC-AB0715 | LN_2 (3.1) | LN_1 (3.2) | | | | |

**TA-SYSTEMS**

- ZETA-T/A
- splTA
- HigBA
- HicAB
- YafQ/RelB
- RelBE
- AbiEii
- Phd/Yoeb
- HipBA

**Supplementary Figure 14**. Toxin-antitoxin systems present in *A. baumannii* plasmid mapped in a phylogenetic tree of the *A. baumannii* isolates constructed using unicopy ribosomal protein genes without recombination signals.
