# Supplementary material for: Structure and Evolution of Acinetobacter baumannii Plasmids
Source: Front Microbiol. 2020 Jun 18;11:1283. doi: 10.3389/fmicb.2020.01283 (PMC7315799; doi:10.3389/fmicb.2020.01283)
Supplement: TABLE S1 — List of plasmids belonging to lineages and their general characteristics. [file Data_Sheet_2.PDF]

Supplementary_Table_1. List of plasmids belonging to lineages and their general characteristics. Plasmids with no assigned plasmid lineage were listed as orphans. The first plasmid of each one of the lineages is the representative plasmid of that lineage. **YEAR**, year of isolation of the strain. **ST,** Sequence Type under Bartual scheme. **GR**, Rep protein GR homology group. **PFAM**, protein family of the Rep protein. **ParAB_SYSTEM**, plasmid partitioning genes of the ParAB family. Plasmid alias is a number that represent the plasmid name and used in the phylogenetic tree of Figure 5. Lines marked in grey shows plasmids with more than one Rep gene. Lines with bold letters indicate those plasmids that were incorporated to the plasmid lineages using 90% of DNA sequence identity and 50% of coverage including the same replication region. GR homology groups marked with a -P as suffix indicates that this particular gene contains premature stops codons or frame-shift mutations.

LN_1

ACCESION_NUMBER	YEAR	PLASMID_NAME	PLASMID_ALIAS	PLASMID_SIZE	STRAIN	ST (OXFORD)	ST (PASTEUR)	GR	REPLICASE_ID	PFAM	ParAB_SYSTEM
CP014292	2015	plasmid unnamed1	6.1	84967	AB34299	436	2	GR6	6.1.1	Replicase_PriCT	ParAB
CP023033	2009	pAba7847b	11.2	80546	7847	208	____	GR6	11.2.1	Replicase_PriCT	ParAB
NZ_CP008707	2008	p1AB5075	54.1	83610	AB5075-UW	945	1	GR6	54.1.1	Replicase_PriCT	ParAB
CP023030	2010	pAba9102a	10.1	95206	9102	231	1	GR6	10.1.1	Replicase_PriCT	ParAB
NZ_CP017647	2015	KAB03 plasmid	72.1	73891	KAB03	451	2	GR6	72.1.1	Replicase_PriCT	ParAB
NZ_CP017645	2015	KAB02 plasmid	71.1	81227	KAB02	369	2	GR6	71.1.1	Replicase_PriCT	ParAB
NC_010606	2005	pACICU2	15.2	64366	ACICU	437	2	GR6-P	15.2.1	____	ParAB
NZ_CP017643	2015	KAB01 plasmid	70.1	70875	KAB01	451	2	GR6-P	70.1.1	____	ParAB
NZ_CP016302	____	pCMCVTAb2-Ab66	69.2	73188	CMC-CR-MDR-Ab66	208	2	GR6	69.2.1	Replicase_PriCT	ParAB
NZ_AYOH01000010	2007	pABUH1-74	41.1	74089	UH9907	208	2	GR6	41.1.1	Replicase_PriCT	ParAB
CP023028	2011	pAba10042b	9.2	110728	10042	473	2	GR6	9.2.1	Replicase_PriCT	ParAB
NZ_CP017655	2015	plasmid unnamed	104.1	72073	KAB07	191	2	GR6	104.1.1	Replicase_PriCT	ParAB
NZ_CP020593	2013	pUSA2_1	89.1	74241	USA2	357	2	GR6	89.1.1	Replicase_PriCT	ParAB
NZ_CP020594	2013	pUSA15_1	90.1	98301	USA15	491	1	GR6	90.1.1	Replicase_PriCT	ParAB
NZ_CP016297	____	pCMCVTAb2-Ab4	67.2	74090	CMC-CR-MDR-Ab4	208	2	GR6	67.2.1	Replicase_PriCT	ParAB
NZ_HG977523	____	pCS01A	95.1	63720	CS01	945	2	GR6-P	95.1.1	____	ParAB
CP007580	2011	pAC30c	5.3	71433	AC30	____	____	GR6	5.3.1	Replicase_PriCT	ParAB
NZ_CP033245	2007	pAba7835b	102.2	80720	7835	227	422	GR6	102.2.1	Replicase_PriCT	ParAB
NZ_ALII01000020	2009	pIS123-67	34.3	67025	IS-123	928	3	GR6	34.3.1	Replicase_PriCT	ParAB
AFDA02000007	2006	pNaval18-74	1.2	74420	Naval-18	993	25	GR6-P	1.2.1	____	ParAB
NZ_CP017649	2015	KAB04 plasmid	73.1	121485	KAB04	191	2	GR6	73.1.1	Replicase_PriCT	ParAB

Supplementary_Table_1

NZ_CP017651	2015	KAB05 plasmid	74.1	70884	KAB05	369	2	GR6	74.1.1	Replicase_PriCT	ParAB
NZ_CP017653	2015	KAB06 plasmid	75.1	70884	KAB06	369	2	GR6	75.1.1	Replicase_PriCT	ParAB
NZ_AEOY01000095	2006	p1ABST2	25.1	63320	3990	437	2	GR6	25.1.1	Replicase_PriCT	ParAB
NZ_CP017657	2015	KAB08 plasmid	76.1	101406	KAB08	208	2	GR6	76.1.1	Replicase_PriCT	ParAB
NZ_CP020573	2013	p15A5_1	83.1	74241	15A5	191	2	GR6	83.1.1	Replicase_PriCT	ParAB
NZ_AFDL01000006	2003	pOIFC143-70	29.2	69518	OIFC143	691	25	GR6	29.2.1	Replicase_PriCT	ParAB
NZ_CP020577	2013	pSSA12_1	84.2	73264	SSA12	191	2	GR6	84.2.1	Replicase_PriCT	ParAB
NZ_CP020582	2013	pJBA13_1	86.1	90972	JBA13	191	2	GR6	86.1.1	Replicase_PriCT	ParAB
NZ_CP020580	2013	pSSMA17_1	85.1	90973	SSMA17	191	2	GR6	85.1.1	Replicase_PriCT	ParAB
NZ_CP015366	2008	pAba3207b	65.2	80546	3207	1321	422	GR6	65.2.1	Replicase_PriCT	ParAB
NC_017163	____	ABKp1	17.1	74451	1656-2	423	2	GR6	17.1.1	Replicase_PriCT	ParAB
NZ_CP020589	2014	p15A34_1	88.1	72076	15A34	872	2	GR6	88.1.1	Replicase_PriCT	ParAB
NZ_CP021787	2003	pA85-3	94.5	86334	A85	____	____	GR6	94.5.1	Replicase_PriCT	ParAB
NZ_CP012008	2012	pAB04-2	60.2	87569	Ab04-mff	447	10	GR6	60.2.1	Replicase_PriCT	ParAB
NZ_HG977524	____	pCS01B	95.2	64165	CS01	945	2	GR6-P	95.2.1	____	ParAB
NZ_HG977527	____	pCR17A	96.1	63795	CR17	208	2	GR6-P	96.1.1	____	ParAB
NZ_HG977528	____	pCR17B	96.2	64479	CR17	208	2	GR6-P	96.2.1	____	ParAB
CP002524	2007	p2ABTCDC0715	3.2	70894	TCDC-AB0715	218	2	GR6	3.1.3	Replicase_PriCT	ParAB
NZ_AFDB02000004	2006	pNaval81-67	27.2	67012	Naval-81	928	3	GR6	27.2.1	Replicase_PriCT	ParAB
NZ_CP014216	2014	plasmid unnamed1	63.1	74241	YU-R612	191	2	GR6	63.1.1	Replicase_PriCT	ParAB
**NZ_CP008851**	2011	pAC29b	55.2	74749	AC29	195	2	GR6	55.2.1	Replicase_PriCT	____

LN_2

ACCESION_NUMBER	YEAR	PLASMID_NAME	PLASMID_ALIAS	PLASMID_SIZE	STRAIN	ST (OXFORD)	ST (PASTEUR)	GR	REPLICASE_ID	PFAM	ParAB_SYSTEM
NZ_CP006964	2011	pPKAB07	52.1	8805	PKAB07	195	2	GR2	52.1.1	Rep_3	____
CP014293	2015	plasmid unnamed2	6.2	15645	AB34299	436	2	GR2/GR2	6.2.1/6.2.3	Rep_3/ Rep_3	____
NZ_CP008850	2011	pAC29a	55.1	8737	AC29	195	2	GR2	55.1.1	Rep_3	____

Supplementary_Table_1

NC_011585	2004	pAB0057	16.1	8731	AB0057	207	1	GR2	16.1.1	Rep_3	____
NZ_CP010782	1982	pA1-1	58.1	8731	A1	231	1	GR2	58.1.1	Rep_3	____
NZ_AYEX01000118	2007	pABUH6a-8.8	36.1	8763	UH9707	208	2	GR2	36.1.1	Rep_3	____
NZ_CM003909	2012	pAB0057	50.1	8781	AB210M	____	____	GR2	50.1.1	Rep_3	____
NZ_CP020576	2013	pSSA12_2	84.1	8730	SSA12	191	2	GR2	84.1.1	Rep_3	____
NZ_CP008708	2008	p2AB5075	54.2	8731	AB5075-UW	945	1	GR2	54.2.1	Rep_3	____
NZ_AFDN01000003	2007	pCanadaBC5-8.7	31.1	8731	Canada BC-5	947	1	GR2	31.1.1	Rep_3	____
NC_021730	2008	p1BJAB0868	23.1	8721	BJAB0868	218	2	GR2	23.1.1	Rep_3	____
NZ_CM003314	2004	pMRSN7339-8.7	43.2	8731	MRSN 7339	____	1	GR2	43.2.1	Rep_3	____
CP007550	2011	pAC12	4.1	8731	AC12	195	2	GR2	4.1.1	Rep_3	____
CP007578	2011	pAC30a	5.1	8685	AC30	____	____	GR2	5.1.2	Rep_3	____
NZ_CP020575	2013	p15A5_2	83.2	8718	15A5	191	2	GR2-P	83.2.1	____	____
NZ_CP021786	2003	pA85-2	94.4	8731	A85	____	____	GR2	94.4.1	Rep_3	____
NC_010402	2001	p2ABAYE	14.2	9661	AYE	231	1	GR2	14.2.1	Rep_3	____
CP002523	2007	p1ABTCDC0715	3.1	8731	TCDC-AB0715	218	2	GR2	3.1.1	Rep_3	____
NZ_JHUI01000005	2008	pAB5075	97.1	8819	AB5075	945	1	GR2	97.1.1	Rep_3	____
NZ_CM003317	2010	pMRSN58-8.7	45.2	8731	MRSN 58	231	1	GR2	45.2.1	Rep_3	____
**CP000523**	____	pAB2	2.2	11302	ATCC 17978	112	437	GR2	2.2.1	Rep_3	____
**NZ_AEOZ01000236**	____	p1ABST78	105.1	26411	3909	944	78	GR2	105.1.1	Rep_3	____
**NZ_CP015486**	2012	pORAB01-3	66.3	15198	ORAB01	208	2	GR2	66.3.1	Rep_3	____
**NZ_CM003741**	2011	plasmid unnamed 1	49.1	5557	MEX11594	231	1	GR2	49.1.1	Rep_3	____
**NZ_CP014217**	2014	plasmid unnamed2	63.2	5465	YU-R612	191	2	GR2	63.2.1	Rep_3	____

Supplementary_Table_1

LN_3A

ACCESION_NUMBER	YEAR	PLASMID_NAME	PLASMID_ALIAS	PLASMID_SIZE	STRAIN	ST (OXFORD)	ST (PASTEUR)	GR	REPLICASE_ID	PFAM	ParAB_SYSTEM
**NZ_CP010398**	**2012**	**p6200-114.848kb**	**56.1**	**114848**	**6200**	**1161**	464	**GR24**	**56.1.1**	**Rep_3**	**(2) ParB, ParA**
NZ_CP020585	2013	pCBA7_1	87.1	111999	CBA7	208	2	GR24	87.1.1	Rep_3	(2) ParB, ParA
NZ_CP016299	____	pCMCVTAb-Ab59	68.1	110967	CMC-CR-MDR-Ab59	208	2	GR24	68.1.1	Rep_3	(2) ParB, ParA
NZ_CP018257	2008	PAF-673	79.1	110964	AF-673	208	2	GR24	79.1.1	Rep_3	(2) ParB, ParA
NZ_CP010780	2014	pAB386	57.1	112157	XH386	208	2	GR24	57.1.1	Rep_3	(2) ParB, ParA
NZ_CP016296	____	pCMCVTAb1-Ab4	67.1	110968	CMC-CR-MDR-Ab4	208	2	GR24	67.1.1	Rep_3	(2) ParB, ParA
NZ_CP016301	____	pCMCVTAb1-Ab66	69.1	110967	CMC-CR-MDR-Ab66	208	2	GR24	69.1.1	Rep_3	(2) ParB, ParA
NZ_AYFW01000101	2007	pABUH4-111	39.1	111007	UH2107	208	2	GR24	39.1.1	Rep_3	(2) ParB, ParA
NC_020524	____	pABTJ2	19.2	110967	MDR-TJ	369	2	GR24	19.2.1	Rep_3	(2) ParB, ParA
NZ_AFDM01000010	2003	pOIFC189-111	30.1	110967	OIFC189	208	2	GR24	30.1.1	Rep_3	(2) ParB, ParA
NZ_CP015484	2012	pORAB01-1	66.1	110965	ORAB01	208	2	GR24	66.1.1	Rep_3	(2) ParB, ParA

LN_3B

ACCESION_NUMBER	YEAR	PLASMID_NAME	PLASMID_ALIAS	PLASMID_SIZE	STRAIN	ST (OXFORD)	ST (PASTEUR)	GR	REPLICASE_ID	PFAM	ParAB_SYSTEM
NC_023031	____	*ZW85p2*	*20.2*	*113866*	*ZW85-1*	*378*	639	*GR24*	*20.2.1*	*Rep_3*	*(2) ParB, ParA*
CP023025	2012	pAba10324c	8.3	113420	10324	771	10	GR24	8.3.1	Rep_3	(2) ParB, ParA

LN_4

ACCESION_NUMBER	YEAR	PLASMID_NAME	PLASMID_ALIAS	PLASMID_SIZE	STRAIN	ST (OXFORD)	ST (PASTEUR)	GR	REPLICASE_ID	PFAM	ParAB_SYSTEM
NZ_CP007714	1997	pABLAC2	53.2	6076	LAC-4	447	10	____	____	____	____
NZ_CP012954	2008	pRAY/pD36-2	61.2	6078	D36	498	81	____	____	____	____
NZ_CM003320	2011	pMRSN3405-6	48.1	6078	MRSN 3405	933	94	____	____	____	____
NZ_CM003315	2011	pMRSN4106-6	44.1	6078	MRSN 4106	933	94	____	____	____	____

Supplementary_Table_1

AFDA02000006	2006	pNaval18-6.1	1.1	6078	Naval-18	993	25	____	____	____	____
NZ_CM003319	2011	pMRSN3942-6	47.1	6078	MRSN 3942	933	94	____	____	____	____
NZ_CM003318	2011	pMRSN3527-6	46.1	6068	MRSN 3527	498	81	____	____	____	____

LN_5

ACCESION_NUMBER	YEAR	PLASMID_NAME	PLASMID_ALIAS	PLASMID_SIZE	STRAIN	ST (OXFORD)	ST (PASTEUR)	GR	REPLICASE_ID	PFAM	ParAB_SYSTEM
NC_017848	____	pABTJ1	19.1	77528	MDR-TJ	369	2	GR25	19.1.1	Replicase_PriCT	ParAB
NZ_CP018144	2014	HRAB-85 plasmid	77.1	77513	HRAB-85	208	2	GR25	77.1.1	Replicase_PriCT	ParAB
NC_021731	2008	p2BJAB0868	23.2	70167	BJAB0868	218	2	GR25	23.2.1	Replicase_PriCT	ParAB
NZ_CP018422	2007	pBJ83	80.1	69069	XDR-BJ83	368	2	GR25	80.1.1	Replicase_PriCT	ParAB
NC_021727	2007	p1BJAB07104	22.1	70170	BJAB07104	368	2	GR25	22.1.1	Replicase_PriCT	ParAB

LN_6

ACCESION_NUMBER	YEAR	PLASMID_NAME	PLASMID_ALIAS	PLASMID_SIZE	STRAIN	ST (OXFORD)	ST (PASTEUR)	GR	REPLICASE_ID	PFAM	ParAB_SYSTEM
CP023027	2011	pAba10042a	9.1	10062	10042	473	2	GR29	9.1.1	Rep_3	____
CP023021	2013	pAba9201a	7.1	9024	9201	934	422	GR29	7.1.1	Rep_3	____
pAba6972a	2013	pAba6972a	101.1	9931	6972	369	2	GR29	101.1.1	Rep_3	____
CP023035	2009	pAba5845a	12.1	9935	5845	417	2	GR29	12.1.1	Rep_3	____

LN_7

ACCESION_NUMBER	YEAR	PLASMID_NAME	PLASMID_ALIAS	PLASMID_SIZE	STRAIN	ST (OXFORD)	ST (PASTEUR)	GR	REPLICASE_ID	PFAM	ParAB_SYSTEM
AFDA02000009	2006	pNaval18-131	1.4	130660	Naval-18	993	25	____	1.4.1	____	ParAB
NZ_AFDK01000004	2003	pOIFC137-122	28.1	122461	OIFC137	106	3	____	28.1.1	____	ParAB
NZ_AFDL01000008	2003	pOIFC143-128	29.4	127633	OIFC143	691	25	____	29.4.1	____	ParAB
NZ_ALAL01000013	2003	pOIFC109-122	33.1	122469	OIFC109	106	3	____	33.1.1	____	ParAB

Supplementary_Table_1

LN_8

ACCESION_NUMBER	YEAR	PLASMID_NAME	PLASMID_ALIAS	PLASMID_SIZE	STRAIN	ST (OXFORD)	ST (PASTEUR)	GR	REPLICASE_ID	PFAM	ParAB_SYSTEM
NZ_CP021344	2014	pB11911	92.1	216780	B11911	____	149	GR3	92.1.1	Rep_3	(2) ParAB
NZ_AP014650	2013	pIOMTU433	35.1	189354	OMTU433	919	622	GR3	35.1.1	Rep_3	(2) ParAB
NZ_CP012007	2012	pAB04-1	60.1	169023	Ab04-mff	447	10	GR3	60.1.1	Rep_3	(2) ParAB
**NZ_CP020596**	2013	pHWBA8_1	91.1	195838	HWBA8	229	25	GR3	91.1.1	Rep_3	(2) ParAB

LN_9

ACCESION_NUMBER	YEAR	PLASMID_NAME	PLASMID_ALIAS	PLASMID_SIZE	STRAIN	ST (OXFORD)	ST (PASTEUR)	GR	REPLICASE_ID	PFAM	ParAB_SYSTEM
NC_010403	2001	p4ABAYE	14.3	2726	AYE	231	1	GR14	14.3.1	Rep_3	____
NZ_CP021783	2003	pA85-1	94.1	2726	A85	____	____	GR14	94.1.1	Rep_3	____
NZ_CM003316	2010	pMRSN58-2.7	45.1	2725	MRSN 58	231	1	GR14-P	45.1.1	____	____

LN_10

ACCESION_NUMBER	YEAR	PLASMID_NAME	PLASMID_ALIAS	PLASMID_SIZE	STRAIN	ST (OXFORD)	ST (PASTEUR)	GR	REPLICASE_ID	PFAM	ParAB_SYSTEM
NC_017172	2006	pMDR-ZJ06	18.1	20301	MDR-ZJ06	643	2	GR3	18.1.1	Rep_3	____
NC_021728	2007	p2BJAB07104	22.2	20139	BJAB07104	368	2	GR3	22.2.1	Rep_3	____
NC_021732	2008	p3BJAB0868	23.3	20139	BJAB0868	218	2	GR3	23.3.3	Rep_3	____

LN_11

ACCESION_NUMBER	YEAR	PLASMID_NAME	PLASMID_ALIAS	PLASMID_SIZE	STRAIN	ST (OXFORD)	ST (PASTEUR)	GR	REPLICASE_ID	PFAM	ParAB_SYSTEM
NC_010605	2005	pACICU1	15.1	28279	ACICU	437	2	GR2/GR10	15.1.1/15.1.2	Rep_3/ Rep_3	ParA
NZ_AEOY01000096	2006	p2ABST2	25.2	21846	3990	437	2	GR2/GR10	25.2.1/25.2.2	Rep_3/ Rep_3	ParA

Supplementary_Table_1

LN_12

ACCESION_NUMBER	YEAR	PLASMID_NAME	PLASMID_ALIAS	PLASMID_SIZE	STRAIN	ST (OXFORD)	ST (PASTEUR)	GR	REPLICASE_ID	PFAM	ParAB_SYSTEM
NC_010401	2001	p1ABAYE	14.1	5644	AYE	231	1	GR11	14.1.1	Rep_3	____
NZ_CM004453	2014	p1M3AC14-8	51.1	5441	M3AC14-8	218	2	GR11	51.1.1	Rep_3	____

LN_13

ACCESION_NUMBER	YEAR	PLASMID_NAME	PLASMID_ALIAS	PLASMID_SIZE	STRAIN	ST (OXFORD)	ST (PASTEUR)	GR	REPLICASE_ID	PFAM	ParAB_SYSTEM
NZ_HG977525	____	pCS01C	95.3	8174	CS01	945	2	GR20	95.3.1	Rep_3	____
NZ_HG977529	____	pCR17C	96.3	8047	CR17	208	2	GR20	96.3.1	Rep_3	____

LN_14

ACCESION_NUMBER	YEAR	PLASMID_NAME	PLASMID_ALIAS	PLASMID_SIZE	STRAIN	ST (OXFORD)	ST (PASTEUR)	GR	REPLICASE_ID	PFAM	ParAB_SYSTEM
NZ_AYFZ01000080	2008	pABUH2a-5.6	40.1	5636	UH19608	124	79	GR27	40.1.1	Rep_3	____
NZ_AYFH01000057	2007	pABUH2b-5.4	37.2	5355	UH7607	124	79	GR27	37.2.1	Rep_3	____

LN_15

ACCESION_NUMBER	YEAR	PLASMID_NAME	PLASMID_ALIAS	PLASMID_SIZE	STRAIN	ST (OXFORD)	ST (PASTEUR)	GR	REPLICASE_ID	PFAM	ParAB_SYSTEM
CP026340	2015	pAba810CPa	103.1	15281	810CP	758	156	GR27	103.1.1	Rep_3	____
NZ_CP018862	2012	11510 plasmid	82.1	13482	11510	758	156	GR27-P	82.1.1	____	____

LN_16

ACCESION_NUMBER	YEAR	PLASMID_NAME	PLASMID_ALIAS	PLASMID_SIZE	STRAIN	ST (OXFORD)	ST (PASTEUR)	GR	REPLICASE_ID	PFAM	ParAB_SYSTEM
NC_020525	2009	pD1279779	21.1	7416	D1279779	942	267	GR3	21.1.1	Rep_3	____
CP023024	2012	pAba10324b	8.2	7143	10324	771	10	GR3	8.2.1	Rep_3	____

**NZ_CP007713**	1997	pABLAC1	53.1	8006	LAC-4	447	10	GR3	53.1.1	Rep_3	____

LN_17

ACCESION_NUMBER	YEAR	PLASMID_NAME	PLASMID_ALIAS	PLASMID_SIZE	STRAIN	ST (OXFORD)	ST (PASTEUR)	GR	REPLICASE_ID	PFAM	ParAB_SYSTEM
NZ_AYFH01000048	2007	pABUH3a-8.2	37.1	8190	UH7607	124	79	GR12	37.1.1	Rep_3	____
NZ_AYFZ01000083	2008	pABUH3b-7.8	40.2	7819	UH19608	124	79	GR12	40.2.1	Rep_3	____

LN_18

ACCESION_NUMBER	YEAR	PLASMID_NAME	PLASMID_ALIAS	PLASMID_SIZE	STRAIN	ST (OXFORD)	ST (PASTEUR)	GR	REPLICASE_ID	PFAM	ParAB_SYSTEM
NC_019985	____	pAbNDM-1	20.1	48368	ZW85-1	378	639	____	____	____	ParB
NZ_CP010399	2012	p6200-47.274kb	56.2	47274	6200	1161	464	____	____	____	ParB

LN_19

ACCESION_NUMBER	YEAR	PLASMID_NAME	PLASMID_ALIAS	PLASMID_SIZE	STRAIN	ST (OXFORD)	ST (PASTEUR)	GR	REPLICASE_ID	PFAM	ParAB_SYSTEM
NZ_AFDO01000021	2006	pNaval17-13	32.1	12636	Naval-17	848	2	GR15	32.1.1	Rep_3	____
NZ_AFDB02000005	2006	pNaval81-13	27.3	12634	Naval-81	928	3	GR15	27.3.1	Rep_3	____

LN_20

ACCESION_NUMBER	YEAR	PLASMID_NAME	PLASMID_ALIAS	PLASMID_SIZE	STRAIN	ST (OXFORD)	ST (PASTEUR)	GR	REPLICASE_ID	PFAM	ParAB_SYSTEM
CP023032	2009	pAba7847a	11.1	13478	7847	208	____	GR26	11.1.1	Rep_3	____
NZ_CP015365	2008	pAba3207a	65.1	13478	3207	1321	422	GR26	65.1.1	Rep_3	____
**NZ_CP033244**	2007	pAba7835a	102.1	7860	7835	227	422	GR26	102.1.1	Rep_3	____

Supplementary_Table_1

LN_21

ACCESION_NUMBER	YEAR	PLASMID_NAME	PLASMID_ALIAS	PLASMID_SIZE	STRAIN	ST (OXFORD)	ST (PASTEUR)	GR	REPLICASE_ID	PFAM	ParAB_SYSTEM
NZ_CP021784	2003	pA85-1a	94.2	2343	A85	____	____	GR16	94.2.1	Rep_3	____
NZ_CM003313	2004	pMRSN7339-2.3	43.1	2343	MRSN 7339	____	1	GR16-P	43.1.1	____	____

ORPHANS

ACCESION_NUMBER	YEAR	PLASMID_NAME	PLASMID_ALIAS	PLASMID_SIZE	STRAIN	ST (OXFORD)	ST (PASTEUR)	GR	REPLICASE_ID	PFAM	ParAB_SYSTEM
NZ_CP015122	2015	ab736 plasmid	64.1	9539	ab736	931	52	GR8	64.1.1	Rep_3	____
NZ_CM003742	2011	plasmid unnamed 2	49.2	4437	MEX11594	231	1	GR12	49.2.1	Rep_3	____
NZ_CM004454	2014	p2M3AC14-8	51.2	18043	M3AC14-8	218	2	____	____	____	____
CP023023	2012	pAba10324a	8.1	5300	10324	771	10	GR7	8.1.1	Rep_3	____
NZ_LT594096	2014	plasmid:2	100.1	8015	BAL062	136	____	GR8	100.1.1	Rep_3	____
CP000522.1	____	pAB1	2.1	13408	ATCC 17978	112	437	GR17	2.1.1	Rep_3	____
NZ_CP012953	2008	pD36-1	61.1	4754	D36	498	81	____	____	____	____
NZ_CP012955	2008	pD36-3	61.3	9276	D36	498	81	GR2	61.3.1	Rep_3	____
NZ_CP012956	2008	pD36-4	61.4	47457	D36	498	81	GR32	61.4.1	Rep_3	ParAB
NZ_CP018255	2009	pAF-401	78.1	17583	AF-401	____	79	GR3	78.1.1	Rep_3	____
NZ_CP021785	2003	pA85-1b	94.3	4484	A85	____	____	____	____	____	____
AFDA02000008	____	pNaval18-8.4	1.3	8422	Naval-18	993	25	GR8	1.3.1	Rep_3	____
AFDA02000010	____	pNaval18-5.7	1.5	5676	Naval-18	993	25	____	____	____	____
AFDA02000011	____	pNaval18-7.0	1.6	7032	Naval-18	993	25	GR20	1.6.1	Rep_3	____
CP007579	2011	pAC30b	5.2	16236	AC30	____	____	____	____	____	____
NZ_AFCZ02000003	____	pOIFC032-101	26.1	101298	OIFC032	472	32	GR24	26.1.1	Rep_3	(2) ParB, ParA
NZ_ALII01000018	2009	pIS123-12	34.1	11600	IS-123	928	3	GR20	34.1.1	Rep_3	____
NZ_ALII01000019	2009	pIS123-18	34.2	17984	IS-123	928	3	GR28	34.2.1	Rep_3	____
NZ_CP008709	2008	p3AB5075	54.3	1967	AB5075-UW	945	1	____	____	____	____
NZ_AYOI01000002	2007	pABUH5-114	42.1	114115	UH10707	350	2	GR31	42.1.1	Rep_3	ParAB

Supplementary_Table_1

NC_021734	____	pBJAB0715	24.1	52268	BJAB0715	642	23	____	____	____	____
CU468231	2014	p1ABSDF	13.1	6106	SDF	____	17	GR1	13.1.1	Rep_3	____
CU468232	2014	p2ABSDF	13.2	25014	SDF	____	17	GR12/GR18	13.2.1/13.2.2	Rep_3/ Rep_3	____
CU468233	2014	p3ABSDF	13.3	24922	SDF	____	17	GR7/GR9/GR15	13.3.1/13.3.2/13.3.3	Rep_3/ Rep_3	____
NZ_CP013925	2012	pKBN10P02143	62.1	52517	KBN10P02143	191	2	GR33	62.1.1	Replicase_PriCT	____
NZ_CP015485	2012	pORAB01-2	66.2	24022	ORAB01	208	2	____	____	____	____
NC_017164	____	ABKp2	17.2	8041	1656-2	423	2	____	____	____	____
NZ_AYFI01000019	2007	pABUH6b-10	38.1	10030	UH7007	281	2	____	____	____	____
NZ_AFDB02000003	____	pNaval81-26	27.1	26089	Naval-81	928	3	GR28/GR20	27.1.1/27.1.2	Rep_3/ Rep_3	____
NZ_CP012005	2014	pAB3	59.1	148955	ATCC 17978-mff	112	437	____	____	RepC	ParAB
NC_010404	2014	p3ABAYE	14.4	94413	AYE	231	1	GR13	14.4.1	Rep_3	ParAB
NZ_AFDL01000005	2003	pOIFC143-2.3	29.1	2277	OIFC143	691	25	GR30	29.1.1	Rep_3	____
NZ_AFDL01000007	2003	pOIFC143-6.2	29.3	6241	OIFC143	691	25	GR3	29.3.1	Rep_3	____
NZ_CP020583	2013	pJBA13_2	86.2	1109	JBA13	191	2	____	____	____	____
NZ_CP021348	2015	pB8300	93.1	25150	B8300	____	____	GR3/GR12	93.1.1/93.1.2	Rep_3/ Rep_3	____
CP026339	2015	pAba810CPb	103.2	16095	810CP	758	156	____	____	____	____
NZ_LN865144	1950	plasmid: II	98.1	7742	R2091	819	126	GR5	98.1.1	Rep_3	____
NZ_AFCZ02000004	____	pOIFC032-8.6	26.2	8604	OIFC032	472	32	____	____	____	____
NZ_CP010400	2012	p6200-9.327kb	56.3	9327	6200	1161	464	GR8	56.3.1	Rep_3	____

Supplementary_Table_2

Supplementary_Table_2. Replicases used to assign GR homology groups. GR groups marked with an asterisk (*) are those originally proposed by Bertini and co-workers (2010) and the Replicases that they used to construct these groups. GR groups marked with two asterisks (**) are those described in this work. **REPLICASE_ID,** are the identification numbers used in the phylogenetic trees of Figure_4 and in Supplementary figures X and Z. **REPLICASE_NAME** column list the names of the Replicase proteins used by Bertini and co-workers (2010) and other authors to construct GR homology groups. **PROTEIN_ID** column indicates the accession numbers of the proteins in GenBank. **GB_ACC_NUMBER** column shows the GenBank accession numbers of the plasmids carrying the Replicases used to construct the GR homology groups. Nucleotide and protein sequences of these replicases are compiled as multifasta files in Supplementary Materials 1 and 2. Headers in the multifasta file are listed in the last column of this table.

GR	REPLICASE_NAME	REPLICASE_ID	PLASMID_NAME	PROTEIN_ID	PFAM	GB_ACC_NUMBER	HEADER IN MULTIFASA (SUPPLEMENTARY MATERIALS)
*GR1	p1ABSDF001 (p1S1)	13.1.1	SDF-p1ABSDF	CAP02936.1	Rep_3	CU468231.1	GR1_CU468231.1_ABSDF_p10001_NCBI CAP02936.1 putative replication protein
*GR2	Aci1	15.1.1	ACICU-pACICU1	WP_001205343.1	Rep_3	NC_010605.1	GR2_NC_010605.1_ACICU_RS18410_NCBI WP_001205343.1 RepB family plasmid replication initiator protein
*GR3	Aci3	__	Ab599	**NOT_FOUND**	__	__	__
*GR3	Aci3	__	Ab203/P203	ADM89092.1	Rep_3	GU978997.1	GR3_1_GU978997.1_p203_repA_NCBI ADM89092.1 plasmid replication protein Aci 3
*GR3	Aci7	__	Ab736	ADM89091.1	Rep_3	GU978996.1	GR3_2_GU978996.1_Abap736_repA_NCBI ADM89091.1 plasmid replication protein Aci 7
*GR4	Aci4	__	Ab844	ADM89093.1	Rep_3	GU978998.1	GR4_GU978998.1_p844_repA_NCBI ADM89093.1 plasmid replication protein Aci 4
*GR5	Aci5	__	Ab537	ADM89094.1	Rep_3	GU978999.1	GR5_GU978999.1_p537_repA_NCBI ADM89094.1 plasmid replication protein Aci 5
*GR6	Aci6	__	ACICU-pACICU2	WP_000217892.1	Replicase_PriCT	NC_010606.1	GR6_NC_010606.1_ACICU_RS18590_NCBI WP_000217892.1 Frameshifted Plasmid replicase
*GR6	__	3.2.1	p2ABTCDC0715	ADX94329.1	Replicase_PriCT	CP002524.1	GR6_CP002524.1_ABTW07_2p036_NCBI ADX94329.1 plasmid replicase protein
*GR7	p3ABSDF002 (p3S2)	13.3.1	SDF-p3ABSDF	CAP02976.1	Rep_3	CU468233.1	GR7_CU468233.1_ABSDF_p30002_NCBI CAP02976.1 DNA replication protein
*GR8	Aci8	__	Ab11921	ADM89095.1	Rep_3	GU979000.1	GR8_1_GU979000.1_p11921_repA_NCBI ADM89095.1 plasmid replication protein Aci 8
*GR8	repM (Aci9)	__	pMAC02	AAT09649.1	Rep_3	AY541809.1	GR8_2_AY541809.1_AY541809_repM_NCBI AAT09649.1 DNA replication protein
*GR9	p3ABSDF0009 (p3S9)	13.3.2	SDF-p3ABSDF	CAP02983.1	Rep_3	CU468233.1	GR9_CU468233.1_ABSDF_p30009_NCBI CAP02983.1 DNA replication protein
*GR10	AciX	15.1.2	ACICU-pACICU1	WP_000845976.1	Rep_3	NC_010605.1	GR10_NC_010605.1_ACICU_RS18440_NCBI WP_000845976.1 RepB family plasmid replication initiator protein
*GR11	p1ABAYE0001 (p1AYE)	14.1.1	AYE-p1ABAYE	WP_001031297.1	Rep_3	NC_010401.1	GR11_NC_010401.1_ABAYE_RS00005_NCBI WP_001031297.1 RepB family plasmid replication initiator protein
*GR12	p2ABSDF0001 (p2S1)	13.2.1	SDF-p2ABSDF	CAP02944.1	Rep_3	CU468232.1	GR12_CU468232.1_ABSDF_p20001_NCBI CAP02944.1 putative replication protein
*GR13	p3ABAYE0002 (p3AYE)	14.4.1	AYE-p3ABAYE	WP_000064928.1	Rep_3	NC_010404.1	GR13_NC_010404.1_ABAYE_RS00110_NCBI WP_000064928.1 RepB family plasmid replication initiator protein
*GR14	p4ABAYE0001 (p4AYE)	14.3.1	AYE-p4ABAYE	WP_001180321.1	Rep_1	NC_010403.1	GR14_NC_010403.1_ABAYE_RS00085_NCBI WP_001180321.1 hypothetical protein
*GR15	p3ABSDF0018 (p3S18)	13.3.3	SDF-p3ABSDF	CAP02992.1	Rep_3	CU468233.1	GR15_CU468233.1_ABSDF_p30018_NCBI CAP02992.1 replication protein
*GR16	repApAB49 (pAB49)	__	pAB49	AAA99423.1	NA	L77992.1	GR16_L77992.1_pAB49_repA_NCBI AAA99423.1 replication protein

Supplementary_Table_2

*GR17	A1s_3471 (A1S3471)	2.1.1	ATCC 17978-pAB1	YP_001083085.1	HTH_17	CP000522.1	GR17_CP000522.1_A1S_3461_NCBI ABO13850.1 DNA replication protein
*GR18	p2ABSDF00025 (p2S25)	13.2.2	SDF-p2ABSDF	CAP02966.1	Rep_3	CU468232.1	GR18_CU468232.1_ABSDF_p20025_NCBI CAP02966.1 putative replication protein
*GR19	rep135040	__	Ab135040	ACX70400.1	Rep_3	GQ861437.1	GR19_GQ861437.1_GQ861437_rep1_NCBI ACX70400.1 putative replicase
GR20	Aci2	__	MAD	**NOT_FOUND**	__	AY665723.1	__
GR20	Aci2	__	VA-566/00-pABVA01	YP_002967453.1	Rep_3	NC_012813	GR20_NC_012813_pABAVE01_01_NCBI YP_002967453.1 DNA replication protein B
GR21	repAci21	__	pAb242_12_1	AUO31881.1	Rep_3	KY984046.1	GR21_KY984046.1_repAci21_NCBI AUO31881.1 Initiator Replication protein
GR22	RepAci22	__	pAb242_25_21	**__**	Rep_3	KY984047	GR22_KY984047_repAci22_NCBI initiator RepB protein
GR23	RepAci23	__	pAb242_25_2	**__**	Rep_3	KY984047	GR23_KY984047_repAci23_NCBI plasmid replication protein Aci23
**GR24	__	26.1.1	pOIFC032-101	WP_000818857.1	Rep_3	NZ_AFCZ02000003.1	GR24_NZ_AFCZ02000003.1_ACIN5032_RS22220_NCBI WP_000818857.1 RepB family plasmid replication initiator protein
**GR25	__	19.1.1	pABTJ1	WP_000633173.1	Replicase_PriCT_HTH_29	NC_017848.1	GR25_NC_017848.1_ABTJ_RS19085_NCBI WP_000633173.1 plasmid replicase
**GR26	__	65.1.1	pAba3207a	WP_063558588.1	Rep_3	NZ_CP015365.1	GR26_NZ_CP015365.1_Aba3207_RS19230_NCBI WP_063558588.1 RepB family plasmid replication initiator protein
**GR27	__	37.2.1	pABUH2b-5.4	WP_004282236.1	Rep_3	NZ_AYFH01000057.1	GR27_NZ_AYFH01000057.1_P676_RS10150_NCBI WP_004282236.1 RepB family plasmid replication initiator protein
**GR28	__	27.1.1	pNaval81-26	WP_000185726.1	Rep_3	NZ_AFDB02000003.1	GR28_NZ_AFDB02000003.1_ACINNAV81_RS23405_NCBI WP_000185726.1 replication protein RepB
**GR29	__	7.1.1		PRJNA311558:CJ991_20650	Rep_3	CP023021	GR29_CP023021_CJ991_20650_NCBI PRJNA311558:CJ991_20650 RepB family plasmid replication initiator protein
**GR30	__	42.1.1	pABUH5-114	WP_000095317.1	Rep_3	NZ_AYOI01000002.1	GR30_NZ_AYOI01000002.1_V427_RS00680_NCBI WP_000095317.1 RepB family plasmid replication initiator protein
**GR31	__	61.4.1	pD36-4	WP_000140303.1	Rep_3	NZ_CP012956.1	GR31_NZ_CP012956.1_AN415_RS19570_NCBI WP_000140303.1 RepB family plasmid replication initiator protein
**GR32	__	62.1.1	pKBN10P02143	WP_059273206.1	Replicase_PriCT	NZ_CP013925.1	GR32_NZ_CP013925.1_KBNAB1_RS20020_NCBI WP_059273206.1 plasmid replicase
**GR33	__	__	pAB3	WP_000743064.1	Replication protein C	NZ_CP012005.1	GR33_NZ_CP012005.1_ACX60_RS18650_NCBI WP_000743064.1 replication protein C

Supplementary_Table_3

Supplementary Table 3. Replicon-associated iterons identified in new GR members.

GR	PLASMID	ALIAS	P_ACC_ NUM.	PROTEIN_ID	REP_FAM.	ITERON_SEQUENCES	N.	L.	AT	D
**GR24**	pOIFC032-101	26.1	NZ_AFCZ02000003.1	WP_000818857.1	Rep_3	AAAGGTACAAAAACTCCGTTAAATC(G/-)CCT(T/A)T	2	30	+	401-
**GR25**	pABTJ1	19.1	NC_017848.1	WP_000633173.1	Rep_PriCT	ND	-	-	-	-
**GR26**	pAba3207a	65.1	NZ_CP015365.1	WP_063558588.1	Rep_3	TATATAGCAACGTTTCCTCGGT	4	22	+	82
**GR27**	pABUH2b-5.4	37.2	NZ_AYFH01000057.1	WP_004282236.1	Rep_3	AAAAAGTACTTTTACCGGAC(A/-)TT	2	22	+	121
**GR28**	pNaval81-26	27.1	NZ_AFDB02000003.1	WP_000185726.1	Rep_3	TATAAAGATAGGTTTAAGGGGT	4	22	+	77
**GR29**	pAba9201a	7.1	CP023021	WP_012780181.1	Rep_3	(T/-)AACTATGACGGATTGACTAC<br>TAACTATGACGGATTGACTACTAACTATGACGGATTGACTAC	4<br>2	21<br>42	+	54
**GR30**	pABUH5-114	42.1	NZ_AYOI01000002.1	WP_000095317.1	Rep_3	GTTTTCGGTAGTTA(A/C)(A/T)A(A/T)TTCC<br>TTTCGGTAGTTAAAAATTCCAA	2<br>2	22<br>22	+	272<br>217
**GR31**	pD36-4	61.4	NZ_CP012956.1	WP_000140303.1	Rep_3	ND	-	-	-	-
**GR32**	pKBN10P02143	62.1	NZ_CP013925.1	WP_059273206.1	Rep_PriCT	ND	-	-	-	-
**GR33**	pAB3		NZ_CP012005.1	WP_000743064.1	RepC	ND	-	-	-	-

GR, Replicase homology group; **P_ACC_NUM**, NCBI_plasmid accession number; **REP_FAM.**, replicase family; **PROTEIN_ID,** accession number of the Rep protein; **N.**, number of repeats; **L.,** length of repeats; **AT**, + an A+T region rich was found close to the tandem repeats; **D**, distance in bp between the initiation codon and the 3’ end of the first iteron.

Supplementary Table 4

STRAIN	PLASMID	ACC._NUMBER	LN	REP_PROTEIN_ID	PFAM	GR
SDF	p2ABSDF	CU468232.1	orphan	CAP02944.1	Rep_3	GR12
				CAP02966.1	Rep_3	GR18
	p3ABSDF	CU468233.1	orphan	CAP02976.1	Rep_3	GR7
				CAP02983.1	Rep_3	GR9
				CAP02992.1	Rep_3	GR15
D36	pD36-4	NZ_CP012956.1	orphan	WP_000140303.1	Rep_3	GR32
				WP_000786839.1	Rep_3	GR22
	pB8300	NZ_CP021348.1	orphan	WP_000845851.1	Rep_3	GR3
				WP_005804946.1	Rep_3	GR12
ACICU	pACICU1	NC_010605.1	LN_11	WP_001205343.1	Rep_3	GR2
				WP_000845976.1	Rep_3	GR10
3990	p2ABST2	NZ_AEOY01000096.1	LN_11	WP_001205343.1	Rep_3	GR2
				WP_000845976.1	Rep_3	GR10

Supplementary Table 4. Plasmids encoding two or more replication proteins. **ACC_NUMBER**, GenBank accession number. **LN**, plasmid lineage. **REP_PROTEIN_ID**, ID of the Rep protein. **GR**, Rep protein homology group.

Supplementary_Table_5. Conjugation genes and Toxin-Antitoxin systems present in plasmids. **MOB_FAM**, Relaxase family. **CONJUGATION**, putative genes involved in conjugation. **TA-SYSTEM**, Toxin-Antitoxin system. **R-M_SYSTEM**, indicate genes encoding Restriction-Modification proteins. Genes product names with a –P indicates that the products are truncated or their putative genes have frameshift mutations or premature stops.

LN_1

ACCESION_NUMBER	PLASMID_NAME	MOB_FAM	CONJUGATION	TA_SYSTEM	R-M_SYSTEM
CP014292	plasmid unnamed1	MOBF	TraG, TraH, TraF, TrbC, TraW, TraV, TraB, TraK, TraE, TraL, TraC, TraU, TraN	Zeta-Toxin/Antitoxin	____
CP023033	pAba7847b	MOBF	TraG, TraH, TraF, TrbC, TraW, TraV, TraB, TraK, TraE, TraL, TraC, TraU, TraN	Zeta-Toxin/Antitoxin	Subunit-R, Subunit-S
NZ_CP008707	p1AB5075	MOBF	TraG, TraH, TraF, TrbC, TraW, TraV, TraB, TraK, TraE, TraL, TraC, TraU, TraN	Zeta-Toxin/Antitoxin	____
CP023030	pAba9102a	MOBF	TraG, TraH, TraF, TrbC, TraW, TraV, TraB, TraK, TraE, TraL, TraC, TraU, TraN	Zeta-Toxin/Antitoxin	Subunit-R, Subunit -S
NZ_CP017647	plasmid	MOBF	TraG, TraH, TraF, TrbC, TraW, TraV, TraB, TraK, TraE, TraL, TraC, TraU, TraN	Zeta-Toxin/Antitoxin	____
NZ_CP017645	plasmid	MOBF	TraG, TraH, TraF, TrbC, TraW, TraV, TraB, TraK, TraE, TraL, TraC, TraU, TraN	Zeta-Toxin/Antitoxin	____
NC_010606	pACICU2	MOBF	TraG, TraH, TraF, TrbC, TraW, TraV, TraB, TraK, TraE, TraL, TraC, TraU, TraN	Zeta-Toxin/Antitoxin	____
NZ_CP017643	plasmid	MOBF	TraG, TraH, TraF, TrbC, TraW, TraV, TraB, TraK, TraE, TraL, TraC, TraU, TraN	Zeta-Toxin/Antitoxin	____
NZ_CP016302	plasmid	MOBF	TraG, TraH, TraF, TrbC, TraW, TraV, TraB, TraK, TraE, TraL, TraC, TraU, TraN	Zeta-Toxin/Antitoxin	____
NZ_AYOH01000010	pABUH1-74	MOBF	TraG, TraH, TraF, TrbC, TraW, TraV, TraB, TraK, TraE, TraL, TraC, TraU, TraN	Zeta-Toxin/Antitoxin	____
CP023028	pAba10042b	MOBF	TraG, TraH, TraF, TrbC, TraW, TraV, TraB, TraK, TraE, TraL, TraC, TraU, TraN	Zeta-Toxin/Antitoxin	Subunit-R, Subunit -S
NZ_CP017655	plasmid unnamed	MOBF	TraG, TraH, TraF, TrbC, TraW, TraV, TraB, TraK, TraE, TraL, TraC, TraU, TraN	Zeta-Toxin/Antitoxin	____
NZ_CP020593	pUSA2_1	MOBF	TraG, TraH, TraF, TrbC, TraW, TraV, TraB, TraK, TraE, TraL, TraC, TraU, TraN	Zeta-Toxin/Antitoxin	____
NZ_CP020594	pUSA15_1	MOBF	TraG, TraH, TraF, TrbC, TraW, TraV, TraB, TraK, TraE, TraL, TraC, TraU, TraN	Zeta-Toxin/Antitoxin	____
NZ_CP016297	pCMCVTAb2-Ab4	MOBF	TraG, TraH, TraF, TrbC, TraW, TraV, TraB, TraK, TraE, TraL, TraC, TraU, TraN	Zeta-Toxin/Antitoxin	____
NZ_HG977523	pCS01A	MOBF	TraG, TraH, TraF, TrbC, TraW, TraV, TraB, TraK, TraE, TraL, TraC, TraU, TraN	Zeta-Toxin/Antitoxin	____
CP007580	pAC30c	MOBF	TraG, TraH, TraF, TrbC, TraW, TraV, TraB, TraK, TraE, TraL, TraC, TraU, TraN	Zeta-Toxin/Antitoxin	____
NZ_CP033245	pAba7835b	MOBF	TraG, TraH, TraF, TrbC, TraW, TraV, TraB, TraK, TraE, TraL, TraC, TraU, TraN	Zeta-Toxin/Antitoxin	Subunit-R, Subunit-S
NZ_ALII01000020	pIS123-67	MOBF	TraG, TraH, TraF, TrbC, TraW, TraV, TraB, TraK, TraE, TraL, TraC, TraU, TraN	Zeta-Toxin/Antitoxin	____
AFDA02000007	pNaval18-74	MOBF	TraG, TraH, TraF, TrbC, TraW, TraV, TraB, TraK, TraE, TraL, TraC, TraU, TraN	Zeta-Toxin/Antitoxin	____
NZ_CP017649	plasmid	MOBF	TraG, TraH, TraF, TrbC, TraW, TraV, TraB, TraK, TraE, TraL, TraC, TraU, TraN	Zeta-Toxin/Antitoxin	____
NZ_CP017651	plasmid	MOBF	TraG, TraH, TraF, TrbC, TraW, TraV, TraB, TraK, TraE, TraL, TraC, TraU, TraN	Zeta-Toxin/Antitoxin	____
NZ_CP017653	plasmid	MOBF	TraG, TraH, TraF, TrbC, TraW, TraV, TraB, TraK, TraE, TraL, TraC, TraU, TraN	Zeta-Toxin/Antitoxin	____
NZ_AEOY01000095	p1ABST2	MOBF	TraG, TraH, TraF, TrbC, TraW, TraV, TraB, TraK, TraE, TraL, TraC, TraU, TraN	Zeta-Toxin/Antitoxin	____
NZ_CP017657	plasmid	MOBF	TraG, TraH, TraF, TrbC, TraW, TraV, TraB, TraK, TraE, TraL, TraC, TraU, TraN	Zeta-Toxin/Antitoxin	____
NZ_CP020573	p15A5_1	MOBF	TraG, TraH, TraF, TrbC, TraW, TraV, TraB, TraK, TraE, TraL, TraC, TraU, TraN	Zeta-Toxin/Antitoxin	____
NZ_AFDL01000006	pOIFC143-70	MOBF	TraG, TraH, TraF, TrbC, TraW, TraV, TraB, TraK, TraE, TraL, TraC, TraU, TraN	Zeta-Toxin/Antitoxin	____

NZ_CP020577	pSSA12_1	MOBF	TraG, TraH, TraF, TrbC, TraW, TraV, TraB, TraK, TraE, TraL, TraC, TraU, TraN	Zeta-Toxin/Antitoxin	____
NZ_CP020582	pJBA13_1	MOBF	TraG, TraH, TraF, TrbC, TraW, TraV, TraB, TraK, TraE, TraL, TraC, TraU, TraN	Zeta-Toxin/Antitoxin	____
NZ_CP020580	pSSMA17_1	MOBF	TraG, TraH, TraF, TrbC, TraW, TraV, TraB, TraK, TraE, TraL, TraC, TraU, TraN	Zeta-Toxin/Antitoxin	____
NZ_CP015366	pAba3207b	MOBF	TraG, TraH, TraF, TrbC, TraW, TraV, TraB, TraK, TraE, TraL, TraC, TraU, TraN	Zeta-Toxin/Antitoxin	Subunit-R, Subunit-S
NC_017163	ABKp1	MOBF	TraG, TraH, TraF, TrbC, TraW, TraV, TraB, TraK, TraE, TraL, TraC, TraU, TraN	Zeta-Toxin/Antitoxin	____
NZ_CP020589	p15A34_1	MOBF	TraG, TraH, TraF, TrbC, TraW, TraV, TraB, TraK, TraE, TraL, TraC, TraU, TraN	Zeta-Toxin/Antitoxin	____
NZ_CP021787	pA85-3	MOBF	TraG, TraH, TraF, TrbC, TraW, TraV, TraB, TraK, TraE, TraL, TraC, TraU, TraN	Zeta-Toxin/Antitoxin	____
NZ_CP012008	pAB04-2	MOBF	TraG, TraH, TraF, TrbC, TraW, TraV, TraB, TraK, TraE, TraL, TraC, TraU, TraN	Zeta-Toxin/Antitoxin	____
NZ_HG977524	pCS01B	MOBF	TraG, TraH, TraF, TrbC, TraW, TraV, TraB, TraK, TraE, TraL, TraC, TraU, TraN	Zeta-Toxin/Antitoxin	____
NZ_HG977527	pCR17A	MOBF	TraG, TraH, TraF, TrbC, TraW, TraV, TraB, TraK, TraE, TraL, TraC, TraU, TraN	Zeta-Toxin/Antitoxin	____
NZ_HG977528	pCR17B	MOBF	TraG, TraH, TraF, TrbC, TraW, TraV, TraB, TraK, TraE, TraL, TraC, TraU, TraN	Zeta-Toxin/Antitoxin	____
CP002524	p2ABTCDC0715	MOBF	TraG, TraH, TraF, TrbC, TraW, TraV, TraB, TraK, TraE, TraL, TraC, TraU, TraN	Zeta-Toxin/Antitoxin	____
NZ_AFDB02000004	pNaval81-67	MOBF	TraG, TraH, TraF, TrbC, TraW, TraV, TraB, TraK, TraE, TraL, TraC, TraU, TraN	Zeta-Toxin/Antitoxin	____
NZ_CP014216	plasmid unnamed1	MOBF	TraG, TraH, TraF, TrbC, TraW, TraV, TraB, TraK, TraE, TraL, TraC, TraU, TraN	Zeta-Toxin/Antitoxin	____
**NZ_CP008851**	pAC29b	____	TraG, TraH, TraF, TrbC, TraU, TraW, TraV, TraB, TraK, TraE, TraL	Zeta-Toxin/Antitoxin	____

LN_2

ACCESION_NUMBER	PLASMID_NAME	MOB_FAM	CONJUGATION	TA_SYSTEM	R-M_SYSTEM
NZ_CP006964	pPKAB07	____	____	SplTA (DUF497/COG3514)	____
CP014293	plasmid unnamed2	____	____	SplTA (DUF497/COG3514)	____
NZ_CP008850	pAC29a	____	____	SplTA (DUF497/COG3514)	____
NC_011585	pAB0057	____	____	SplTA (DUF497/COG3514)	____
NZ_CP010782	pA1-1	____	____	SplTA (DUF497/COG3514)	____
NZ_AYEX01000118	pABUH6a-8.8	____	____	SplTA (DUF497/COG3514)	____
NZ_CM003909	pAB0057	____	____	SplTA (DUF497/COG3514)	____
NZ_CP020576	pSSA12_2	____	____	SplTA (DUF497/COG3514)	____
NZ_CP008708	p2AB5075	____	____	SplTA (DUF497/COG3514)	____
NZ_AFDN01000003	pCanadaBC5-8.7	____	____	SplTA (DUF497/COG3514)	____
NC_021730	p1BJAB0868	____	____	SplTA (DUF497/COG3514)	____
NZ_CM003314	pMRSN7339-8.7	____	____	SplTA (DUF497/COG3514)	____
CP007550	pAC12	____	____	SplTA (DUF497/COG3514)	____
CP007578	pAC30a	____	____	SplTA (DUF497/COG3514)	____

NZ_CP020575	p15A5_2	____	____	SplTA (DUF497/COG3514)	____
NZ_CP021786	pA85-2	____	____	SplTA (DUF497/COG3514)	____
NC_010402	p2ABAYE	____	____	SplTA (DUF497/COG3514)	____
CP002523	p1ABTCDC0715	____	____	SplTA (DUF497/COG3514)	____
NZ_JHUI01000005	pAB5075	____	____	SplTA (DUF497/COG3514)	____
NZ_CM003317	pMRSN58-8.7	____	____	SplTA (DUF497/COG3514)	____
**CP000523.1**	pAB2	____	____	SplTA (DUF497/COG3514)	____
**NZ_AEOZ01000236**	p1ABST78	MOBQ	____	SplTA (DUF497/COG3514)	____
**NZ_CP015486**	pORAB01-3	____	____	SplTA (DUF497/COG3514)	____
**NZ_CM003741**	plasmid unnamed 1	____	____	____	____
**NZ_CP014217**	plasmid unnamed2	____	____	SplTA (DUF497/COG3514)	____

LN_3A

ACCESION_NUMBER	PLASMID_NAME	MOB_FAM	CONJUGATION	TA_SYSTEM	R-M_SYSTEM
**NZ_CP010398**	**p6200-114.848kb**	____	____	____	____
NZ_CP020585	pCBA7_1	____	____	____	____
NZ_CP016299	plasmid	____	____	____	____
NZ_CP018257	PAF-673	____	____	____	____
NZ_CP010780	pAB386	____	____	____	____
NZ_CP016296	pCMCVTAb1-Ab4	____	____	____	____
NZ_CP016301	plasmid	____	____	____	____
NZ_AYFW01000101	pABUH4-111	____	____	____	____
NC_020524	pABTJ2	____	____	____	____
NZ_AFDM01000010	pOIFC189-111	____	____	____	____
NZ_CP015484	pORAB01-1	____	____	____	____

LN_3B

ACCESION_NUMBER	PLASMID_NAME	MOB_FAM	CONJUGATION	TA_SYSTEM	R-M_SYSTEM
NC_023031	*ZW85p2*	____	____	____	*Subunit-M, Subunit-S*
CP023025	pAba10324c	____	____	____	Subunit-M, Subunit-S

Supplementary_Table_5

LN_4

ACCESION_NUMBER	PLASMID_NAME	MOB_FAM	CONJUGATION	TA_SYSTEM	R-M_SYSTEM
NZ_CP007714	pABLAC2	MOBH	____	____	____
NZ_CP012954	pRAY/pD36-2	MOBH	____	____	____
NZ_CM003320	pMRSN3405-6	MOBH	____	____	____
NZ_CM003315	pMRSN4106-6	MOBH	____	____	____
AFDA02000006	pNaval18-6.1	MOBH	____	____	____
NZ_CM003319	pMRSN3942-6	MOBH	____	____	____
NZ_CM003318	pMRSN3527-6	MOBH	____	____	____

LN_5

ACCESION_NUMBER	PLASMID_NAME	MOB_FAM	CONJUGATION	TA_SYSTEM	R-M_SYSTEM
NC_017848	pABTJ1	MOBF	TraG, TraH, TraF, TraN, TraU, TraW, TraV, TraB, TraK, TraE, TraL, TrbC, TraC	Zeta-Toxin/Antitoxin	____
NZ_CP018144	plasmid	MOBF	TraG, TraH, TraF, TraN, TraU, TraW, TraV-p, TraB, TraK-p, TraE, TraL-p, TrbC, TraC	Zeta-Toxin/Antitoxin	____
NC_021731	p2BJAB0868	MOBF	TraG, TraH, TraF, TraN, TraU, TraW, TraV, TraB, TraK, TraE, TraL, TrbC, TraC	Zeta-Toxin/Antitoxin	____
NZ_CP018422	pBJ83	MOBF	TraG, TraH, TraF, TraN, TraU, TraW, TraV-p, TraB-p, TraK-p, TraE-p, TraL, TrbC, TraC	Zeta-Toxin/Antitoxin	____
NC_021727	p1BJAB07104	MOBF	TraG, TraH, TraF, TraN, TraU, TraW, TraV, TraB, TraK, TraE, TraL, TrbC, TraC	Zeta-Toxin/Antitoxin	____

LN_6

ACCESION_NUMBER	PLASMID_NAME	MOB_FAM	CONJUGATION	TA_SYSTEM	R-M_SYSTEM
CP023027	pAba10042a	MOBQ	____	____	____
CP023021	pAba9201a	MOBQ	____	____	____
pAba6972a	pAba6972a	MOBQ	____	____	____
CP023035	pAba5845a	MOBQ	____	____	____

LN_7

ACCESION_NUMBER	PLASMID_NAME	MOB_FAM	CONJUGATION	TA_SYSTEM	R-M_SYSTEM
AFDA02000009	pNaval18-131	____	TraU, TraW, TraY, TraI, TraM, TraN	AbiEii	____

NZ_AFDK01000004	pOIFC137-122	____	TraU, TraW, TraY, TraI, TraM, TraN	AbiEii	____
NZ_AFDL01000008	pOIFC143-128	____	TraU, TraW, TraY, TraI, TraM, TraN	AbiEii	____
NZ_ALAL01000013	pOIFC109-122	____	TraU, TraW, TraY, TraI, TraM, TraN	AbiEii	____

LN_8

ACCESION_NUMBER	PLASMID_NAME	MOB_FAM	CONJUGATION	TA_SYSTEM	R-M_SYSTEM
NZ_CP021344	pB11911	____	TraU, TraW, TraY, TraI, TraM, TraN	AbiEii	Subunit-M
NZ_AP014650	pIOMTU433	____	TraU, TraW, TraY, TraI, TraM, TraN	AbiEii	Subunit-M
NZ_CP012007	pAB04-1	____	TraU, TraW, TraY, TraI, TraM, TraN	AbiEii	Subunit-M
**NZ_CP020596**	pHWBA8_1	____	TraW, TraY,TraI	____	Subunit-M

LN_9

ACCESION_NUMBER	PLASMID_NAME	MOB_FAM	CONJUGATION	TA_SYSTEM	R-M_SYSTEM
NC_010403	p4ABAYE	____	____	____	____
NZ_CP021783	pA85-1	____	____	____	____
NZ_CM003316	pMRSN58-2.7	____	____	____	____

LN_10

ACCESION_NUMBER	PLASMID_NAME	MOB_FAM	CONJUGATION	TA_SYSTEM	R-M_SYSTEM
NC_017172	pMDR-ZJ06	____	____	____	____
NC_021728	p2BJAB07104	____	____	____	____
NC_021732	p3BJAB0868	____	____	____	____

LN_11

ACCESION_NUMBER	PLASMID_NAME	MOB_FAM	CONJUGATION	TA_SYSTEM	R-M_SYSTEM
NC_010605	pACICU1	____	____	RelB/RelE	____
NZ_AEOY01000096	p2ABST2	____	____	RelB/RelE	____

Supplementary_Table_5

LN_12

ACCESION_NUMBER	PLASMID_NAME	MOB_FAM	CONJUGATION	TA_SYSTEM	R-M_SYSTEM
NC_010401	p1ABAYE	MOBQ	____	Phd/YoeB	____
NZ_CM004453	p1M3AC14-8	MOBQ	____	Phd/YoeB	____

LN_13

ACCESION_NUMBER	PLASMID_NAME	MOB_FAM	CONJUGATION	TA_SYSTEM	R-M_SYSTEM
NZ_HG977525	pCS01C	____	____	SplTA (DUF497/COG3514)	____
NZ_HG977529	pCR17C	____	____	SplTA (DUF497/COG3514)	____

LN_14

ACCESION_NUMBER	PLASMID_NAME	MOB_FAM	CONJUGATION	TA_SYSTEM	R-M_SYSTEM
NZ_AYFZ01000080	pABUH2a-5.6	MOBQ	____	YafQ/RelB	____
NZ_AYFH01000057	pABUH2b-5.4	MOBQ	____	YafQ/RelB	____

LN_15

ACCESION_NUMBER	PLASMID_NAME	MOB_FAM	CONJUGATION	TA_SYSTEM	R-M_SYSTEM
CP026340	pAba810CPa	MOBQ	____	SplTA (DUF497/COG3514)	____
NZ_CP018862	plasmid	MOBQ	____	SplTA (DUF497/COG3514)	____

LN_16

ACCESION_NUMBER	PLASMID_NAME	MOB_FAM	CONJUGATION	TA_SYSTEM	R-M_SYSTEM
NC_020525	*pD1279779*	____	____	*HigB/HigA*	____
CP023024	pAba10324b	____	____	HigB/HigA	____
**NZ_CP007713**	pABLAC1	____	____	HigB/HigA	____

Supplementary_Table_5

LN_17

ACCESION_NUMBER	PLASMID_NAME	MOB_FAM	CONJUGATION	TA_SYSTEM	R-M_SYSTEM
NZ_AYFH01000048	pABUH3a-8.2	MOBQ	____	____	____
NZ_AYFZ01000083	pABUH3b-7.8	MOBQ	____	____	____

LN_18

ACCESION_NUMBER	PLASMID_NAME	MOB_FAM	CONJUGATION	TA_SYSTEM	R-M_SYSTEM
NC_019985	pAbNDM-1	MOBQ	____	____	____
NZ_CP010399	p6200-47.274kb	MOBQ	____	____	____

LN_19

ACCESION_NUMBER	PLASMID_NAME	MOB_FAM	CONJUGATION	TA_SYSTEM	R-M_SYSTEM
NZ_AFDO01000021	pNaval17-13	____	____	____	____
NZ_AFDB02000005	pNaval81-13	____	____	____	____

LN_20

ACCESION_NUMBER	PLASMID_NAME	MOB_FAM	CONJUGATION	TA_SYSTEM	R-M_SYSTEM
CP023032	pAba7847a	____	____	____	____
NZ_CP015365	pAba3207a	____	____	____	____
**NZ_CP033244**	pAba7835a	____	____	____	____

LN_21

ACCESION_NUMBER	PLASMID_NAME	MOB_FAM	CONJUGATION	TA_SYSTEM	R-M_SYSTEM
NZ_CP021784	pA85-1a	____	____	____	____
NZ_CM003313	pMRSN7339-2.3	____	____	____	____

Supplementary_Table_5

ORPHANS

ACCESION_NUMBER	PLASMID_NAME	MOB_FAM	CONJUGATION	TA_SYSTEM	R-M_SYSTEM
NZ_CP015122	plasmid	MOBQ	____	HigBA	____
NZ_CM003742	plasmid unnamed 2	____	____	____	____
NZ_CM004454	p2M3AC14-8	MOBQ	____	HigBA	____
CP023023	pAba10324a	MOBQ	____	____	____
NZ_LT594096	plasmid:2	MOBQ	____	HigBA	____
CP000522.1	pAB1	____	____	HigBA	____
NZ_CP012953	pD36-1	MOBP	____	____	____
NZ_CP012955	pD36-3	____	____	____	____
NZ_CP012956	pD36-4	____	____	SplTA (DUF497/COG3514)	____
NZ_CP018255	pAF-401	____	____	HigBA	____
NZ_CP021785	pA85-1b	MOBQ	____	____	____
AFDA02000008	pNaval18-8.4	MOBQ	____	____	____
AFDA02000010	pNaval18-5.7	MOBQ	____	____	____
AFDA02000011	pNaval18-7.0	MOBQ	____	____	____
CP007579	pAC30b	____	____	____	____
NZ_AFCZ02000003	pOIFC032-101	____	____	____	Subunit-M, subunit-S
NZ_AFCZ02000004	pOIFC032-8.6		____	HicAB	____
NZ_ALII01000018	pIS123-12	____	____	____	____
NZ_ALII01000019	pIS123-18	____	____	____	____
NZ_CP008709	p3AB5075	____	____	____	____
NZ_CP010400	p6200-9.327kb	____	____	HigBA	____
NZ_AYOI01000002	pABUH5-114	____	____	____	____
NC_021734	pBJAB0715	____	____	HigBA	____
CU468231	p1ABSDF	____	____	____	____
CU468232	p2ABSDF	MOBQ	____	YafQ/RelB	HpaIIM, HpaIIR
CU468233	p3ABSDF	MOBQ	____	____	Subunit-R, Subunit-M
NZ_CP013925	pKBN10P02143	MOBF	TraG, TraH, TraF, TrbC, TraU, TraW, TraV, TraB, TraK, TraE, TraL,TraN	____	____
NZ_CP015485	pORAB01-2	____	____	____	____
NC_017164	ABKp2	____	____	SplTA (DUF497/COG3514)	____
NZ_AYFI01000019	pABUH6b-10	MOBQ	____	SplTA (DUF497/COG3514)	____

NZ_AFDB02000003	pNaval81-26	____	____	SplTA (DUF497/COG3514)	____
NZ_CP012005	pAB3	____	TraW, TraY, TraI, TraM, TraN	____	____
NC_010404	p3ABAYE	____	____	HigBA, HipBA, RelBE	____
NZ_AFDL01000005	pOIFC143-2.3	____	____	____	____
NZ_AFDL01000007	pOIFC143-6.2	____	____	____	____
NZ_CP020583	pJBA13_2	____	____	____	____
NZ_CP021348	pB8300	MOBQ	____	SplTA (DUF497/COG3514)	____
CP026339	pAba810CPb	____	____	____	____
NZ_LN865144	plasmid: II	MOBQ	____	____	____

Supplementary_Table_6

Supplementary_Table_6. IS elements present in plasmids. Number of IS elements in the plasmid is written between brackets before de IS name, if the number of such element is higher than one.

LN_1

ACCESION_NUMBER	PLASMID_NAME	IS
CP014292	plasmid unnamed1	(2) ISAba1, (2) ISAba125, (2) ISAba33
CP023033	pAba7847b	(2) ISAba125
NZ_CP008707	p1AB5075	(2) ISAba125
CP023030	pAba9102a	(3) ISAba125
NZ_CP017647	plasmid	(3) ISAba10
NZ_CP017645	plasmid	____
NC_010606	pACICU2	ISAba125
NZ_CP017643	plasmid	____
NZ_CP016302	plasmid	(2) SAba1, (2) ISAba125
NZ_AYOH01000010	pABUH1-74	(2) ISAba1, (2) ISAba125
CP023028	pAba10042b	(2) ISAba125
NZ_CP017655	plasmid unnamed	____
NZ_CP020593	pUSA2_1	ISAba10
NZ_CP020594	pUSA15_1	(2) ISAba1, (2) ISAba125, (2) ISAba13
NZ_CP016297	pCMCVTAb2-Ab4	(2) ISAba1, (2) ISAba125
NZ_HG977523	pCS01A	ISAba125
CP007580	pAC30c	ISAba25
NZ_CP033245	pAba7835b	(2) ISAba125
NZ_ALII01000020	pIS123-67	____
AFDA02000007	pNaval18-74	(2) ISAba25, ISAba125
NZ_CP017649	plasmid	(4) ISAba10
NZ_CP017651	plasmid	____
NZ_CP017653	plasmid	____
NZ_AEOY01000095	p1ABST2	ISAba125

Supplementary_Table_6

NZ_CP017657	plasmid	(3) ISAba10
NZ_CP020573	p15A5_1	ISAba10
NZ_AFDL01000006	pOIFC143-70	ISAba25
NZ_CP020577	pSSA12_1	ISAba1
NZ_CP020582	pJBA13_1	(2) ISAba10
NZ_CP020580	pSSMA17_1	(2) ISAba10
NZ_CP015366	pAba3207b	(2) ISAba125
NC_017163	ABKp1	(2) ISAba1
NZ_CP020589	p15A34_1	____
NZ_CP021787	pA85-3	(2) ISAba1
NZ_CP012008	pAB04-2	(2) ISAba1, ISAba125
NZ_HG977524	pCS01B	ISAba125
NZ_HG977527	pCR17A	ISAba125
NZ_HG977528	pCR17B	ISAba125
CP002524	p2ABTCDC0715	ISAba125
NZ_AFDB02000004	pNaval81-67	____
NZ_CP014216	plasmid unnamed1	ISAba10
**NZ_CP008851**	pAC29b	ISAba25, ISAba1, ISAba33

LN_2

ACCESION_NUMBER	PLASMID_NAME	IS
NZ_CP006964	pPKAB07	____
CP014293	plasmid unnamed2	____
NZ_CP008850	pAC29a	____
NC_011585	pAB0057	____
NZ_CP010782	pA1-1	____
NZ_AYEX01000118	pABUH6a-8.8	____

Supplementary_Table_6

NZ_CM003909	pAB0057	____
NZ_CP020576	pSSA12_2	____
NZ_CP008708	p2AB5075	____
NZ_AFDN01000003	pCanadaBC5-8.7	____
NC_021730	p1BJAB0868	____
NZ_CM003314	pMRSN7339-8.7	____
CP007550	pAC12	____
CP007578	pAC30a	____
NZ_CP020575	p15A5_2	____
NZ_CP021786	pA85-2	____
NC_010402	p2ABAYE	____
CP002523	p1ABTCDC0715	____
NZ_JHUI01000005	pAB5075	____
NZ_CM003317	pMRSN58-8.7	____
**CP000523**	pAB2	____
**NZ_AEOZ01000236**	p1ABST78	ISAba25, ISAba2, ISAba29, ISAba19, ISAba34, IS15DII, IS26, IS15DIV, IS15DI, ISAba3, ISAba18, ISPa14
**NZ_CP015486**	pORAB01-3	ISAba1, ISAba33
**NZ_CM003741**	plasmid unnamed 1	____
**NZ_CP014217**	plasmid unnamed2	____

LN_3A

ACCESION_NUMBER	PLASMID_NAME	IS
**NZ_CP010398**	**p6200-114.848kb**	**ISAha2, IS1008**
NZ_CP020585	pCBA7_1	ISAba10, ISAha2
NZ_CP016299	plasmid	ISAha2
NZ_CP018257	PAF-673	ISAha2
NZ_CP010780	pAB386	ISAba1, ISAha2, ISAba33

Supplementary_Table_6

NZ_CP016296	pCMCVTAb1-Ab4	ISAha2
NZ_CP016301	plasmid	ISAha2
NZ_AYFW01000101	pABUH4-111	ISAha2
NC_020524	pABTJ2	ISAha2
NZ_AFDM01000010	pOIFC189-111	ISAha2
NZ_CP015484	pORAB01-1	ISAha2

LN_3B

ACCESION_NUMBER	PLASMID_NAME	IS
NC_023031	*ZW85p2*	*ISAba1*
CP023025	pAba10324c	ISAba1

LN_5

ACCESION_NUMBER	PLASMID_NAME	IS
NC_017848	pABTJ1	(2) ISAba1
NZ_CP018144	plasmid	(2) ISAba1
NC_021731	p2BJAB0868	ISAba1
NZ_CP018422	pBJ83	____
NC_021727	p1BJAB07104	ISAba1

LN_7

ACCESION_NUMBER	PLASMID_NAME	IS
AFDA02000009	pNaval18-131	(2) ISAba25, ISEc57, ISAba1, ISAba125, IS1008
NZ_AFDK01000004	pOIFC137-122	ISVsa3, IS1008
NZ_AFDL01000008	pOIFC143-128	ISAba25, ISAba1, ISVsa3, IS1008
NZ_ALAL01000013	pOIFC109-122	ISVsa3, IS1008, ISAba1

Supplementary_Table_6

LN_8

ACCESION_NUMBER	PLASMID_NAME	IS
NZ_CP021344	pB11911	ISAcsp1, ISEc29, (2) IS10A, ISAba34, ISAba14, (2) ISVsa3, ISEc28, ISVsa3, IS10A, ISAba19, (2) IS1008, (2) IS26, (2) IS91, IS15DIV, (2) IS1007
NZ_AP014650	pIOMTU433	ISAcsp1, ISEc29, IS10A, ISVsa3, ISEc28, ISVsa3, IS10A, (2) IS1008, (2) IS26, IS91, IS91
NZ_CP012007	pAB04-1	ISEc29, IS10A, ISAba1, (2) ISVsa3, ISEc28, ISVsa3, IS10A, IS1008, (2) IS26, (2) IS91
**NZ_CP020596**	pHWBA8_1	ISEc29, IS10A, ISAba1, ISAba11, IS17, ISVsa3, ISAba12, IS1008, IS26, IS15DIV, IS15DII, IS1007, IS15DI, ISOur1, ISAba33

LN_10

ACCESION_NUMBER	PLASMID_NAME	IS
NC_017172	pMDR-ZJ06	ISAba24, ISEc29, ISEc28, (2) IS15DI, (2) IS26, IS91
NC_021728	p2BJAB07104	ISAba24, ISEc29, ISEc28, (2) IS15DI, IS91
NC_021732	p3BJAB0868	ISAba24, ISEc29, ISEc28, (2) IS15DI, IS91

LN_11

ACCESION_NUMBER	PLASMID_NAME	IS
NC_010605	pACICU1	(2) ISAba2, (2) ISAba3, (2) IS15DII, (2) IS15DIV
NZ_AEOY01000096	p2ABST2	(2) ISAba2, ISAba3, IS15DII, IS15DIV

LN_12

ACCESION_NUMBER	PLASMID_NAME	IS
NC_010401	p1ABAYE	____
NZ_CM004453	p1M3AC14-8	____

Supplementary_Table_6

LN_14

ACCESION_NUMBER	PLASMID_NAME	IS
NZ_AYFZ01000080	pABUH2a-5.6	____
NZ_AYFH01000057	pABUH2b-5.4	____

LN_15

ACCESION_NUMBER	PLASMID_NAME	IS
CP026340	pAba810CPa	ISAba27
NZ_CP018862	plasmid	ISAba27

LN_17

ACCESION_NUMBER	PLASMID_NAME	IS
NZ_AYFH01000048	pABUH3a-8.2	____
NZ_AYFZ01000083	pABUH3b-7.8	____

LN_18

ACCESION_NUMBER	PLASMID_NAME	IS
NC_019985	pAbNDM-1	ISAba14, (2) ISAba125
NZ_CP010399	p6200-47.274kb	ISAba14, (2) ISAba125

LN_19

ACCESION_NUMBER	PLASMID_NAME	IS
NZ_AFDO01000021	pNaval17-13	ISAjo2
NZ_AFDB02000005	pNaval81-13	ISAba22, ISAjo2.

Supplementary_Table_6

LN_20

ACCESION_NUMBER	PLASMID_NAME	IS
CP023032	pAba7847a	ISAba3 (2) ISAba125
NZ_CP015365	pAba3207a	ISAba3, (2) ISAba125
**NZ_CP033244**	pAba7835a	____

ORPHANS

ACCESION_NUMBER	PLASMID_NAME	IS
NZ_CP015122	plasmid	____
NZ_CM003742	plasmid unnamed 2	____
NZ_CM004454	p2M3AC14-8	ISAba11
CP023023	pAba10324a	____
NZ_LT594096	plasmid:2	____
CP000522	pAB1	____
NZ_CP012953	pD36-1	____
NZ_CP012955	pD36-3	____
NZ_CP012956	pD36-4	ISAba8, ISAba32, ISAba1, IS26, IS15DII, IS15DIV, IS15DI, IS1006, ISAba33,
NZ_CP018255	pAF-401	ISAba1, ISAjo2, ISAba33
NZ_CP021785	pA85-1b	____
AFDA02000008	pNaval18-8.4	____
AFDA02000010	pNaval18-5.7	____
AFDA02000011	pNaval18-7.0	____
CP007579	pAC30b	ISAba24, ISEc29
NZ_AFCZ02000003	pOIFC032-101	ISEc57, ISAba26, ISAba22, ISAba13, ISAba19, ISAba2, ISAba29, ISAba34, ISAba18
NZ_AFCZ02000004	pOIFC032-8.6	____
NZ_ALII01000018	pIS123-12	ISAba22
NZ_ALII01000019	pIS123-18	ISAjo2, ISPa14

Supplementary_Table_6

NZ_CP008709	p3AB5075	____
NZ_CP010400	p6200-9.327kb	____
NZ_AYOI01000002	pABUH5-114	IS26, IS15DIV, IS15DII, IS15DI
NC_021734	pBJAB0715	ISAba43, ISCfr1, ISPsp5, ISAba125, ISAba19, ISAba12, IS17, ISPa94, ISAba29, ISAba2, ISAba34, ISAba3, ISOur1, IS1008, ISAba18, ISPa14
CU468231	p1ABSDF	____
CU468232	p2ABSDF	ISAba7
CU468233	p3ABSDF	ISAba7, ISAba6
NZ_CP013925	pKBN10P02143	____
NZ_CP015485	pORAB01-2	ISAba1, ISAba33
NC_017164	ABKp2	____
NZ_AYFI01000019	pABUH6b-10	____
NZ_AFDB02000003	pNaval81-26	ISAjo2, ISPa14
NZ_CP012005	pAB3	ISAba18, ISAba1, ISAba11, ISVsa3, ISAba12, IS17, ISAba29, ISAba19, ISAba2, ISAba33, ISAba34
NC_010404	p3ABAYE	____
NZ_AFDL01000005	pOIFC143-2.3	____
NZ_AFDL01000007	pOIFC143-6.2	____
NZ_CP020583	pJBA13_2	____
NZ_CP021348	pB8300	ISAba22
CP026339	pAba810CPb	ISAba27
NZ_LN865144	plasmid: II	____

Supplementary_Table_7. Antibiotic resistance genes located in plasmids. **AMINOGLYC.**, Aminoglycosides resistance genes; **SULPH.**, Sulphonamide resistance genes; **B-LACTAM**. Beta-lactam antibiotics resistance genes. **MACR**. Macrolide, Lincosamide and Streptogramin B resistance genes; **PHEN**. Phenicol resistance genes; **TETRAC**. Tetracycline resistance genes; **RIFAMP**. Rifampicin resistance genes, **FLUOROQ**. Fluoroquinolone resistance genes. **TRIMETH.**, Trimethoprim resistance genes. **INT**, Integron; **AbaR**, AbaR family.

LN_1

ACCESION_NUMBER	PLASMID_NAME	AMINOGLYC.	SULPH.	B-LACTAM.	MACR.	PHEN.	TETRAC.	RIFAMP.	FLUOROQ.	TRIMETH.	INT.	AbaR
CP014292	plasmid unnamed1	*aph(3')-Via*	____	____	____	____	____	____	____	____	____	____
CP023033	pAba7847b	*aph(3')-Via*	____	____	____	____	____	____	____	____	____	____
NZ_CP008707	p1AB5075	*aadB, aadA2, strA, strB, aacA4, aph(3')-Via*	*sul1*	*blaGES-11*	____	*cmlA1*	____	____	*aac(6')Ib-cr*	*dfrA7*	*Intl1*	____
CP023030	pAba9102a	*aph(3')-Via*	____	____	____	____	____	____	____	____	____	____
NZ_CP017647	plasmid	____	____	____	____	____	____	____	____	____	____	____
NZ_CP017645	plasmid	____	____	____	____	____	____	____	____	____	____	____
NC_010606	pACICU2	____	____	____	____	____	____	____	____	____	____	____
NZ_CP017643	plasmid	____	____	____	____	____	____	____	____	____	____	____
NZ_CP016302	plasmid	____	____	*blaOXA-23*	____	____	____	____	____	____	____	____
NZ_AYOH01000010	pABUH1-74	*aph(3')-Via*	____	*blaOXA-23*	____	____	____	____	____	____	____	____
CP023028	pAba10042b	*aph(3')-Via*	____	____	____	____	____	____	____	____	____	____
NZ_CP017655	plasmid unnamed	____	____	____	____	____	____	____	____	____	____	____
NZ_CP020593	pUSA2_1	____	____	____	____	____	____	____	____	____	____	____
NZ_CP020594	pUSA15_1	*aph(3')-Via*	____	*blaOXA-23*	____	____	____	____	____	____	____	AbaR4
NZ_CP016297	pCMCVTAb2-Ab4	*aph(3')-Via*	____	*blaOXA-23*	____	____	____	____	____	____	____	____
NZ_HG977523	pCS01A	____	____	____	____	____	____	____	____	____	____	____
CP007580	pAC30c	____	____	____	____	____	____	____	____	____	____	____
NZ_CP033245	pAba7835b	*aph(3')-Via*	____	____	____	____	____	____	____	____	____	____
NZ_ALII01000020	pIS123-67	____	____	____	____	____	____	____	____	____	____	____
AFDA02000007	pNaval18-74	____	____	____	____	____	____	____	____	____	____	____
NZ_CP017649	plasmid	____	____	____	____	____	____	____	____	____	____	____
NZ_CP017651	plasmid	____	____	____	____	____	____	____	____	____	____	____
NZ_CP017653	plasmid	____	____	____	____	____	____	____	____	____	____	____
NZ_AEOY01000095	p1ABST2	____	____	____	____	____	____	____	____	____	____	____
NZ_CP017657	plasmid	____	____	____	____	____	____	____	____	____	____	____
NZ_CP020573	p15A5_1	____	____	____	____	____	____	____	____	____	____	____

NZ_AFDL01000006	pOIFC143-70	____	____	____	____	____	____	____	____	____	____	____
NZ_CP020577	pSSA12_1	____	____	____	____	____	____	____	____	____	____	____
NZ_CP020582	pJBA13_1	____	____	____	____	____	____	____	____	____	____	____
NZ_CP020580	pSSMA17_1	____	____	____	____	____	____	____	____	____	____	____
NZ_CP015366	pAba3207b	*aph(3')-Via*	____	____	____	____	____	____	____	____	____	____
NC_017163	ABKp1	____	____	____	____	____	____	____	____	____	____	____
NZ_CP020589	p15A34_1	____	____	____	____	____	____	____	____	____	____	____
NZ_CP021787	pA85-3	____	____	*blaOXA-23*	____	____	____	____	____	____	____	AbaR4
NZ_CP012008	pAB04-2	____	____	*blaOXA-23*	____	____	____	____	____	____	____	AbaR4
NZ_HG977524	pCS01B	____	____	____	____	____	____	____	____	____	____	____
NZ_HG977527	pCR17A	____	____	____	____	____	____	____	____	____	____	____
NZ_HG977528	pCR17B	____	____	____	____	____	____	____	____	____	____	____
CP002524	p2ABTCDC0715	____	____	____	____	____	____	____	____	____	____	____
NZ_AFDB02000004	pNaval81-67	____	____	____	____	____	____	____	____	____	____	____
NZ_CP014216	plasmid unnamed1	____	____	____	____	____	____	____	____	____	____	____
**NZ_CP008851**	pAC29b	____	____	*blaOXA-23*	____	____	____	____	____	____	____	____

LN_2

ACCESION_NUMBER	PLASMID_NAME	AMINOGLYC.	SULPH.	B-LACTAM.	MACR.	PHEN.	TETRAC.	RIFAMP.	FLUOROQ.	TRIMETH.	INT.	AbaR
NZ_CP006964	pPKAB07	____	____	____	____	____	____	____	____	____	____	____
CP014293	plasmid unnamed2	____	____	____	____	____	____	____	____	____	____	____
NZ_CP008850	pAC29a	____	____	____	____	____	____	____	____	____	____	____
NC_011585	pAB0057	____	____	____	____	____	____	____	____	____	____	____
NZ_CP010782	pA1-1	____	____	____	____	____	____	____	____	____	____	____
NZ_AYEX01000118	pABUH6a-8.8	____	____	____	____	____	____	____	____	____	____	____
NZ_CM003909	pAB0057	____	____	____	____	____	____	____	____	____	____	____
NZ_CP020576	pSSA12_2	____	____	____	____	____	____	____	____	____	____	____
NZ_CP008708	p2AB5075	____	____	____	____	____	____	____	____	____	____	____
NZ_AFDN01000003	pCanadaBC5-8.7	____	____	____	____	____	____	____	____	____	____	____
NC_021730	p1BJAB0868	____	____	____	____	____	____	____	____	____	____	____
NZ_CM003314	pMRSN7339-8.7	____	____	____	____	____	____	____	____	____	____	____
CP007550	pAC12	____	____	____	____	____	____	____	____	____	____	____

CP007578	pAC30a	——	——	——	——	——	——	——	——	——	——	——
NZ_CP020575	p15A5_2	——	——	——	——	——	——	——	——	——	——	——
NZ_CP021786	pA85-2	——	——	——	——	——	——	——	——	——	——	——
NC_010402	p2ABAYE	——	——	——	——	——	——	——	——	——	——	——
CP002523	p1ABTCDC0715	——	——	——	——	——	——	——	——	——	——	——
NZ_JHUI01000005	pAB5075	——	——	——	——	——	——	——	——	——	——	——
NZ_CM003317	pMRSN58-8.7	——	——	——	——	——	——	——	——	——	——	——
**CP000523**	pAB2	——	——	——	——	——	——	——	——	——	——	——
**NZ_AEOZ01000236**	p1ABST78	——	——	*blaOXA-58*	——	——	——	——	——	——	——	——
**NZ_CP015486**	pORAB01-3	——	——	*blaOXA-237*	——	——	——	——	——	——	——	——
**NZ_CM003741**	plasmid unnamed 1	——	——	——	——	——	——	——	——	——	——	——
**NZ_CP014217**	plasmid unnamed2	——	——	——	——	——	——	——	——	——	——	——

LN_3B

ACCESION_NUMBER	PLASMID_NAME	AMINOGLYC.	SULPH.	B-LACTAM.	MACR.	PHEN.	TETRAC.	RIFAMP.	FLUOROQ.	TRIMETH.	INT.	AbaR
NC_023031	*ZW85p2*	——	*sul2*	——	——	——	*tet(B)*	——	——	——	——	——
CP023025	pAba10324c	——	*sul2*	——	——	——	*tet(B)*	——	——	——	——	——

LN_4

ACCESION_NUMBER	PLASMID_NAME	AMINOGLYC.	SULPH.	B-LACTAM.	MACR.	PHEN.	TETRAC.	RIFAMP.	FLUOROQ.	TRIMETH.	INT.	AbaR
NZ_CP007714	pABLAC2	*aadB*	——	——	——	——	——	——	——	——	——	——
NZ_CP012954	pRAY/pD36-2	*aadB*	——	——	——	——	——	——	——	——	——	——
NZ_CM003320	pMRSN3405-6	*aadB*	——	——	——	——	——	——	——	——	——	——
NZ_CM003315	pMRSN4106-6	*aadB*	——	——	——	——	——	——	——	——	——	——
AFDA02000006	pNaval18-6.1	*aadB*	——	——	——	——	——	——	——	——	——	——
NZ_CM003319	pMRSN3942-6	*aadB*	——	——	——	——	——	——	——	——	——	——
NZ_CM003318	pMRSN3527-6	*aadB*	——	——	——	——	——	——	——	——	——	——

LN_5

ACCESION_NUMBER	PLASMID_NAME	AMINOGLYC.	SULPH.	B-LACTAM.	MACR.	PHEN.	TETRAC.	RIFAMP.	FLUOROQ.	TRIMETH.	INT.	AbaR
NC_017848	pABTJ1	____	____	*blaOXA-23*	____	____	____	____	____	____	____	____
NZ_CP018144	plasmid	____	____	*blaOXA-23*	____	____	____	____	____	____	____	____
NC_021731	p2BJAB0868	____	____	____	____	____	____	____	____	____	____	____
NZ_CP018422	pBJ83	____	____	____	____	____	____	____	____	____	____	____
NC_021727	p1BJAB07104	____	____	____	____	____	____	____	____	____	____	____

LN_6

ACCESION_NUMBER	PLASMID_NAME	AMINOGLYC.	SULPH.	B-LACTAM.	MACR.	PHEN.	TETRAC.	RIFAMP.	FLUOROQ.	TRIMETH.	INT.	AbaR
CP023027	pAba10042a	____	____	*blaOXA-72*	____	____	____	____	____	____	____	____
CP023021	pAba9201a	____	____	*blaOXA-72*	____	____	____	____	____	____	____	____
pAba6972a	pAba6972a	____	____	*blaOXA-72*	____	____	____	____	____	____	____	____
CP023035	pAba5845a	____	____	*blaOXA-72*	____	____	____	____	____	____	____	____

LN_7

ACCESION_NUMBER	PLASMID_NAME	AMINOGLYC.	SULPH.	B-LACTAM.	MACR.	PHEN.	TETRAC.	RIFAMP.	FLUOROQ.	TRIMETH.	INT.	AbaR
AFDA02000009	pNaval18-131	*strA,strB*	*sul2*	____	____	____	____	____	____	____	____	____
NZ_AFDK01000004	pOIFC137-122	*strA,strB*	*sul2*	____	____	____	____	____	____	____	____	____
NZ_AFDL01000008	pOIFC143-128	*strA,strB*	*sul2*	____	____	____	____	____	____	____	____	____
NZ_ALAL01000013	pOIFC109-122	*strA,strB*	*sul2*	____	____	____	____	____	____	____	____	____

LN_8

ACCESION_NUMBER	PLASMID_NAME	AMINOGLYC.	SULPH.	B-LACTAM.	MACR.	PHEN.	TETRAC.	RIFAMP.	FLUOROQ.	TRIMETH.	INT.	AbaR
NZ_CP021344	pB11911	*armA, strA, strB*	*sul2, sul1*	*blaPER-7*	*msr(E), mph(E)*	cmlA1	____	*ARR-2*	____	____	IntI1	____
NZ_AP014650	pIOMTU433	*armA, strA, strB*	*sul2, sul1*	*blaPER-7*	*msr(E), mph(E)*	cmlA1	____	*ARR-2*	____	____	IntI1	____
NZ_CP012007	pAB04-1	*armA, strA, strB*	*sul2, sul1*	*blaPER-7*	*msr(E), mph(E)*	cmlA1	*tet(B)*	____	____	____	IntI1	____

**NZ_CP020596**	pHWBA8_1	*aac(3)-Iia, armA*	*sul1, sul2*	*blaPER-7*	*msr(E), mph(E)*	*cmlA1*	*tet(B)*	*ARR-2*	____	____	____	____

LN_10

ACCESION_NUMBER	PLASMID_NAME	AMINOGLYC.	SULPH.	B-LACTAM.	MACR.	PHEN.	TETRAC.	RIFAMP.	FLUOROQ.	TRIMETH.	INT.	AbaR
NC_017172	pMDR-ZJ06	*armA, aadA1, aph(3')-Ic, aac(3)-Ia*	*sul1*	____	*mph(E), msr(E)*	____	____	____	____	____	IntI1	____
NC_021728	p2BJAB07104	*armA, aadA1, aph(3')-Ic*	*sul1*	____	*mph(E), msr(E)*	____	____	____	*aac(6')Ib-cr*	____	IntI1	____
NC_021732	p3BJAB0868	*armA, aadA1, aph(3')-Ic*	*sul1*	____	*mph(E), msr(E)*	*catB8*	____	____	*aac(6')Ib-cr*	____	IntI1	____

LN_11

ACCESION_NUMBER	PLASMID_NAME	AMINOGLYC.	SULPH.	B-LACTAM.	MACR.	PHEN.	TETRAC.	RIFAMP.	FLUOROQ.	TRIMETH.	INT.	AbaR
NC_010605	pACICU1	____	____	(2) *blaOXA-58*	____	____	____	____	____	____	____	____
NZ_AEOY01000096	p2ABST2	____	____	*blaOXA-58*	____	____	____	____	____	____	____	____

LN_14

ACCESION_NUMBER	PLASMID_NAME	AMINOGLYC.	SULPH.	B-LACTAM.	MACR.	PHEN.	TETRAC.	RIFAMP.	FLUOROQ.	TRIMETH.	INT.	AbaR
NZ_AYFZ01000080	pABUH2a-5.6	____	____	*blaOXA-24*	____	____	____	____	____	____	____	____
NZ_AYFH01000057	pABUH2b-5.4	____	____	____	____	____	____	____	____	____	____	____

LN_17

ACCESION_NUMBER	PLASMID_NAME	AMINOGLYC.	SULPH.	B-LACTAM.	MACR.	PHEN.	TETRAC.	RIFAMP.	FLUOROQ.	TRIMETH.	INT.	AbaR
NZ_AYFH01000048	pABUH3a-8.2	____	____	*blaOXA-24*	____	____	____	____	____	____	____	____
NZ_AYFZ01000083	pABUH3b-7.8	____	____	____	____	____	____	____	____	____	____	____

LN_18

ACCESION_NUMBER	PLASMID_NAME	AMINOGLYC.	SULPH.	B-LACTAM.	MACR.	PHEN.	TETRAC.	RIFAMP.	FLUOROQ.	TRIMETH.	INT.	AbaR
NC_019985	pAbNDM-1	*aph(3')-Via*	____	*blaNDM-1*	____	____	____	____	____	____	____	____
NZ_CP010399	p6200-47.274kb	*aph(3')-Via*	____	*blaNDM-16*	____	____	____	____	____	____	____	____

Supplementary_Table_7

LN_20

ACCESION_NUMBER	PLASMID_NAME	AMINOGLYC.	SULPH.	B-LACTAM.	MACR.	PHEN.	TETRAC.	RIFAMP.	FLUOROQ.	TRIMETH.	INT.	AbaR
CP023032	pAba7847a	____	____	*blaOXA-58*	____	____	____	____	____	____	____	____
NZ_CP015365	pAba3207a	____	____	*blaOXA-58*	____	____	____	____	____	____	____	____
**NZ_CP033244**	pAba7835a	*aph(3')-Via*	____	____	____	____	____	____	____	____	____	____

ORPHANS

ACCESION_NUMBER	PLASMID_NAME	AMINOGLYC.	SULPH.	B-LACTAM.	MACR.	PHEN.	TETRAC.	RIFAMP.	FLUOROQ.	TRIMETH.	INT.	AbaR
NZ_CP015122	plasmid	____	____	____	____	____	____	____	____	____	____	____
NZ_CM003742	plasmid unnamed 2	____	____	____	____	____	____	____	____	____	____	____
NZ_CM004454	p2M3AC14-8	____	____	____	____	____	____	____	____	____	____	____
CP023023	pAba10324a	____	____	____	____	____	____	____	____	____	____	____
NZ_LT594096	plasmid:2	____	____	____	____	____	____	____	____	____	____	____
CP000522.1	pAB1	____	____	____	____	____	____	____	____	____	____	____
NZ_CP012953	pD36-1	____	____	____	____	____	____	____	____	____	____	____
NZ_CP012955	pD36-3	____	____	____	____	____	____	____	____	____	____	____
NZ_CP012956	pD36-4	*aph(3')-Ia*	*sul2*	____	____	____	____	____	____	____	____	____
NZ_CP018255	pAF-401	____	____	*blaOXA-235*	____	____	____	____	____	____	____	____
NZ_CP021785	pA85-1b	____	____	____	____	____		____	____	____	____	____
AFDA02000008	pNaval18-8.4	____	____	____	____	____	*tet(39)*	____	____	____	____	____
AFDA02000010	pNaval18-5.7	*aph(3')-Ia*	____	____	____	____	____	____	____	____	____	____
AFDA02000011	pNaval18-7.0	____	____	____	____	____	____	____	____	____	____	____
CP007579	pAC30b	*aph(3')-Ic*	____	____	*msr(E)*	____	____	____	____	____	____	____
NZ_AFCZ02000003	pOIFC032-101	____	____	____	____	____	____	____	____	____	____	____
NZ_AFCZ02000004	pOIFC032-8.6	____	____	____	____	____	____	____	____	____	____	____
NZ_ALII01000018	pIS123-12	____	____	____	____	____	____	____	____	____	____	____
NZ_ALII01000019	pIS123-18	____	____	____	____	____	____	____	____	____	____	____
NZ_CP008709	p3AB5075	____	____	____	____	____	____	____	____	____	____	____

Supplementary_Table_7

NZ_CP010400	p6200-9.327kb	____	____	____	____	____	____	____	____	____	____	____
NZ_AYOI01000002	pABUH5-114	*aph(3')-Ia*	____	____	*msr(E)*	____	____	____	____	____	____	____
NC_021734	pBJAB0715	*aph(3')-Via, aac(3)-Iid*	____	*blaOXA-58*	____	____	____	____	____	____	____	____
CU468231	p1ABSDF	____	____	____	____	____	____	____	____	____	____	____
CU468232	p2ABSDF	____	____	____	____	____	____	____	____	____	____	____
CU468233	p3ABSDF	____	____	____	____	____	____	____	____	____	____	____
NZ_CP013925	pKBN10P02143	____	____	____	____	____	____	____	____	____	____	____
NZ_CP015485	pORAB01-2	____	*sul2*	____	____	____	____	____	____	____	____	____
NC_017164	ABKp2	____	____	____	____	____	____	____	____	____	____	____
NZ_AYFI01000019	pABUH6b-10	____	____	____	____	____	____	____	____	____	____	____
NZ_AFDB02000003	pNaval81-26	____	____	____	____	____	____	____	____	____	____	____
NZ_CP012005	pAB3	____	*sul2*	____	____	____	____	____	____	____	____	____
NC_010404	p3ABAYE	____	____	____	____	____	____	____	____	____	____	____
NZ_AFDL01000005	pOIFC143-2.3	____	____	____	____	____	____	____	____	____	____	____
NZ_AFDL01000007	pOIFC143-6.2	____	____	____	____	____	____	____	____	____	____	____
NZ_CP020583	pJBA13_2	____	____	____	____	____	____	____	____	____	____	____
NZ_CP021348	pB8300	____	____	____	____	____	____	____	____	____	____	____
NZ_LN865144	plasmid: II	____	____	____	____	____	____	____	____	____	____	____

Supplementary_Table_8. Gene flux between plasmid lineages. **TOTAL_KB** the ammoun of different in information that one lineage shares with other. **EFFLUX_GENES** genes that are located within the shared information. Numbers within brakets before gene name indicates the number of times this sequence is present in the shared sequence information.

REFERENCE	QUERY	TOTAL_BP	FLUX_GENES
LN_1	LN_3	2347	(2) Transposase ISAba1
	LN_5	2357	(2) Transposase ISAba1
	LN_7	10039	(2) ISAba25, Orf2 domain protein
			Transposase ISAba25
			Integrase core domain protein
			Transposase ISAba25
			ISAba25 C-terminal element
			(2) Transposase ISAba1
			(2) Transposase ISAba125
	LN_8	8997	(2) Transposase ISAba1
			Aminoglycoside O-phosphotransferase APH(3'')-Ib
			Aminoglycoside O-phosphotransferase APH(6)-Id
			Hypothetical protein (WP_005028401.1)
			Hypothetical protein (WP_000743213.1)
			Class 1 integron integrase IntI1
			Chloramphenicol efflux MFS transporter CmlA5
			Hypothetical protein (AB994_RS01200)
			Quaternary ammonium efflux SMR transporter QacE delta 1
			(2) Sulfonamide-resistant Sul1
	LN_3B	2347	(2) Transposase ISAba1

Supplementary_Table_8

	LN_18	3260	(2) Transposase ISAba125
			Transposase IS30
	LN_20	2193	(2) Transposase ISAba125

REFERENCE	QUERY	TOTAL_bp	FLUX_GENES
LN_2	LN_11	3625	RepB protein
			DNA-binding protein
			Hypothetical protein (WP_000504218.1)
			Hypothetical protein (WP_001014301.1)
			Hypothetical protein (WP_012576333.1)
	LN_13	5544	Toxin-Antitoxin system spITA (COG3514)
			Toxin-Antitoxin system spITA (DUF497)
			TonB dependent receptor
			Hypothetical protein (WP_004781077.1)
			Hypothetical protein (WP_000504218.1)
			Hypothetical protein (WP_001014301.1)
	LN_15	4820	Toxin-Antitoxin system spITA (COG3514)
			Toxin-Antitoxin system spITA (DUF497)
			TonB dependent receptor
			Hypothetical protein (WP_004781077.1)
			Septicolysin
	LN_19	1020	Hypothetical protein (WP_000504218.1)
			Hypothetical protein (WP_001014301.1)
			Hypothetical protein (WP_012576333.1)
			Hypothetical protein (WP_005265294.1)
			Hypothetical protein (WP_005173935.1)

Supplementary_Table_8

	LN_20	2855	DNA-binding protein
			Hypothetical protein (WP_000504218.1)
			Hypothetical protein (WP_001014301.1)
			Hypothetical protein (WP_012576333.1)

REFERENCE	QUERY	TOTAL_bp	FLUX_GENES
LN_3A	LN_7	3693	Transposase IS1008
			S-(hydroxymethyl)glutathione dehydrogenase
			Metal/formaldehyde-sensitive transcriptional repressor
			Transposase ISAba1
	LN_8	1692	S-(hydroxymethyl)glutathione dehydrogenase
			Metal/formaldehyde-sensitive transcriptional repressor

REFERENCE	QUERY	TOTAL_bp	FLUX_GENES
LN_3B	LN_1	2347	(2) Transposase ISAba1
	LN_5	2362	(2) Transposase ISAba1
	LN_7	4221	Transposase ISAba1
			Hypothetical protein (ACINNAV18_A0048)
			Sulfonamide-resistant Sul2
			Phosphoglucomutase/phosphomannomutase, alpha/beta/alpha domain I protein
			Transposase ISVsa3
			Transposase IS1008
	LN_8	4634	(2) Transposase IS6
			Transposase ISVsa3

Supplementary_Table_8

			Hypothetical protein (WP_001445143.1)
			Phosphoglucosamine mutase
			Sulfonamide-resistant dihydropteroate synthase Sul2
			Nucleotide pyrophosphohydrolase

REFERENCE	QUERY	TOTAL_bp	FLUX_GENES
LN_5	LN_1	2357	(2) Transposase ISAba1
	LN_3	2358	(2) Transposase ISAba1
	LN_7	2358	(2) Transposase ISAba1
	LN_8	2358	(2) Transposase ISAba1
	LN_3B	2362	(2) Transposase ISAba1

REFERENCE	QUERY	TOTAL_bp	FLUX_GENES
LN_6	LN_17	1410	Beta-lactamase OXA-72

REFERENCE	QUERY	TOTAL_bp	FLUX_GENES
LN_7	LN_1	10039	(2) ISAba25, Orf2 domain protein
			Transposase ISAba25
			Integrase core domain protein
			Transposase ISAba25
			ISAba25 C-terminal element
			(2) Transposase ISAba1
			(2) Transposase ISAba125

Supplementary_Table_8

	LN_3A	3693	Transposase IS1008
			S-(hydroxymethyl)glutathione dehydrogenase
			Metal/formaldehyde-sensitive transcriptional repressor
			Transposase ISAba1
	LN_5	2358	(2) Transposase ISAba1
	LN_8	95347	Hypothetical protein (ACINNAV18_A0003)
			Hypothetical protein (ACINNAV18_A0004)
			Hypothetical protein (ACINNAV18_A0005)
			Hypothetical protein (ACINNAV18_A0006)
			Hypothetical protein (ACINNAV18_A0007)
			Hypothetical protein (ACINNAV18_A0008)
			Hypothetical protein (ACINNAV18_A0009)
			Hypothetical protein (ACINNAV18_A0010)
			Hypothetical protein (ACINNAV18_A0011)
			Hypothetical protein (ACINNAV18_A0012)
			Hypothetical protein (ACINNAV18_A0013)
			Putative lipoprotein
			Hypothetical protein (ACINNAV18_A0015)
			Hypothetical protein (ACINNAV18_A0016)
			Hypothetical protein (ACINNAV18_A0017)
			Hypothetical protein (ACINNAV18_A0018)
			Hypothetical protein (ACINNAV18_A0019)
			Hypothetical protein (ACINNAV18_A0020)
			Hypothetical protein (ACINNAV18_A0021)
			Transcriptional regulator, TetR family
			Hypothetical protein (ACINNAV18_A0023)
			Hypothetical protein (ACINNAV18_A0024)
			Hypothetical protein (ACINNAV18_A0025)
			Hypothetical protein (ACINNAV18_A0026)
			Hypothetical protein (ACINNAV18_A0027)

Supplementary_Table_8

			Hypothetical protein (ACINNAV18_A0028)
			Hypothetical protein (ACINNAV18_A0029)
			Transcriptional regulator, TetR family
			Hypothetical protein (ACINNAV18_A0031)
			Peptidase, M48 family
			GHKL domain protein
			Hypothetical protein (ACINNAV18_A0034)
			Hypothetical protein (ACINNAV18_A0035)
			Hypothetical protein (ACINNAV18_A0036)
			Hypothetical protein (ACINNAV18_A0037)
			Phage integrase, N-terminal SAM domain protein
			Site-specific tyrosine recombinase XerC
			Hypothetical protein (ACINNAV18_A0040)
			Hypothetical protein (ACINNAV18_A0041)
			Hypothetical protein (ACINNAV18_A0042)
			Hypothetical protein (ACINNAV18_A0046)
			Transposase zinc-binding domain protein
			Hypothetical protein (ACINNAV18_A0055)
			Aminoglycoside resistence strB
			Aminoglycoside resistence strA
			Hypothetical protein (ACINNAV18_A0058)
			Hypothetical protein (ACINNAV18_A0059)
			Hypothetical protein (ACINNAV18_A0060)
			Putative DNA topoisomerase I
			Hypothetical protein (ACINNAV18_A0062)
			Hypothetical protein (ACINNAV18_A0063)
			IS66 C-terminal element
			Hypothetical protein (ACINNAV18_A0068)
			Hypothetical protein (ACINNAV18_A0069)
			PF07308 family protein
			Transglycosylase SLT domain protein
			Hypothetical protein (ACINNAV18_A0072)
			Hypothetical protein (ACINNAV18_A0073)

Supplementary_Table_8

			Hypothetical protein (ACINNAV18_A0074)
			Hypothetical protein (ACINNAV18_A0075)
			Hypothetical protein (ACINNAV18_A0076)
			Hypothetical protein (ACINNAV18_A0077)
			Hypothetical protein (ACINNAV18_A0078)
			Integrase core domain protein
			S-(hydroxymethyl)glutathione dehydrogenase
			Metal-sensitive transcriptional repressor
			Transposase, IS4 family
			Hypothetical protein (ACINNAV18_A0083)
			Hypothetical protein (ACINNAV18_A0084)
			Hypothetical protein (ACINNAV18_A0085)
			Hypothetical protein (ACINNAV18_A0086)
			Conjugal protein TraU
			Hypothetical protein (ACINNAV18_A0088)
			Hypothetical protein (ACINNAV18_A0089)
			Hypothetical protein (ACINNAV18_A0090)
			Hypothetical protein (ACINNAV18_A0091)
			Hypothetical protein (ACINNAV18_A0092)
			TrbI-like protein
			Conjugal protein TraN
			Conjugal protein TraM
			Hypothetical protein (ACINNAV18_A0096)
			Hypothetical protein (ACINNAV18_A0101)
			Putative lipoprotein
			Conjugal protein TraI
			Type II/IV secretion system domain protein
			StbA protein
			Hypothetical protein (ACINNAV18_A0106)
			Hypothetical protein (ACINNAV18_A0107)
			Hypothetical protein (ACINNAV18_A0108)
			Hypothetical protein (ACINNAV18_A0109)
			Hypothetical protein (ACINNAV18_A0110)

Supplementary_Table_8

			Hypothetical protein (ACINNAV18_A0111)
			Hypothetical protein (ACINNAV18_A0112)
			Partition protein ParA
			Partition protein ParB
			ImpB/MucB/SamB family protein
			Peptidase S24-like protein
			Nucleotidyl transferase, PF08843 family (AbiEii/AbiGii toxin family protein)
			PF13338 domain protein
			Hypothetical protein (ACINNAV18_A0119)
			Hypothetical protein (ACINNAV18_A0120)
			Hypothetical protein (ACINNAV18_A0121)
			Hypothetical protein (ACINNAV18_A0122)
			Peptidase, M23 family
			Hypothetical protein (ACINNAV18_A0124)
			Conjugal protein TraY
			Hypothetical protein (ACINNAV18_A0126)
			Hypothetical protein (ACINNAV18_A0127)
			Hypothetical protein (ACINNAV18_A0128)
			Hypothetical protein (ACINNAV18_A0131)
			Hypothetical protein (ACINNAV18_A0132)
			Hypothetical protein (ACINNAV18_A0133)
			Conjugal protein TraW
			Hypothetical protein (ACINNAV18_A0135)
			Hypothetical protein (ACINNAV18_A0136)
			Hypothetical protein (ACINNAV18_A0137)
			Hypothetical protein (ACINNAV18_A0138)
			Hypothetical protein (ACINNAV18_A0139)
			Hypothetical protein (ACINNAV18_A0140)
			Helicase C-like protein
			Hypothetical protein (AB994_RS00210)
			Hypothetical protein (WP_000493808.1)
			Hypothetical protein (WP_000369329.1)
			Hypothetical protein (WP_002038214.1)

			Hypothetical protein (WP_001101148.1)
			Hypothetical protein (WP_000064550.1)
			Hypothetical protein (WP_000568115.1)
			Hypothetical protein (WP_001075210.1)
			Hypothetical protein (WP_000700025.1)
			Hypothetical protein (WP_001226062.1)
			Hypothetical protein (WP_000202450.1)
			Hypothetical protein (WP_000640998.1)
			Hypothetical protein (WP_000481946.1)
			Hypothetical protein (WP_002011618.1)
			Hypothetical protein (WP_000071965.1)
			Hypothetical protein (AB994_RS00290)
			Hypothetical protein (WP_000824500.1)
			H-NS histone
			Hypothetical protein (WP_001102765.1)
			Hypothetical protein (WP_000182799.1)
			Hypothetical protein (WP_000594604.1)
			Helicase
			Hypothetical protein (WP_002011620.1)
			Hypothetical protein (WP_002016363.1)
			Hypothetical protein (WP_000445287.1)
			Hypothetical protein (WP_002011578.1)
			Hypothetical protein (WP_001211405.1)
			Hypothetical protein (WP_000730798.1)
			Hypothetical protein (WP_000871698.1)
			Hypothetical protein (WP_000773909.1)
			Hypothetical protein (WP_000994248.1)
			Hypothetical protein (WP_000236940.1)
			Hypothetical protein (WP_002015162.1)
			Hypothetical protein (WP_001217963.1)
			Hypothetical protein (WP_000123889.1)
			Hypothetical protein (WP_000287492.1)
			Hypothetical protein (WP_000069170.1)

			Hypothetical protein (WP_000956387.1)
			Hypothetical protein (WP_000733607.1)
			Hypothetical protein (WP_002011623.1)
			Hypothetical protein (WP_000343986.1)
			Hypothetical protein (WP_002011569.1)
			Hypothetical protein (WP_000271057.1)
			DNA polymerase V
			DNA polymerase V subunit UmuC
			Hypothetical protein (WP_001177226.1)
			Hypothetical protein (WP_001085267.1)
			Hypothetical protein (WP_000444265.1)
			Hypothetical protein (WP_000765384.1)
			Hypothetical protein (WP_000851072.1)
			Hypothetical protein (WP_000069415.1)
			Hypothetical protein (WP_001135704.1)
			Hypothetical protein (WP_000389973.1)
			Hypothetical protein (WP_001103687.1)
			Hypothetical protein (WP_000706892.1)
			Hypothetical protein (WP_000643578.1)
			Hypothetical protein (WP_000665168.1)
			Hypothetical protein (WP_000064221.1)
			Hypothetical protein (WP_000447535.1)
			Hypothetical protein (WP_000036098.1)
			Hypothetical protein (WP_000608872.1)
			Hypothetical protein (WP_000573069.1)
			Hypothetical protein (WP_001009075.1)
			Hypothetical protein (WP_002015136.1)
			Hypothetical protein (WP_000931461.1)
			Hypothetical protein (WP_004895603.1)
			(2) Transposase IS1008
			Hypothetical protein (WP_000644488.1)
			Hypothetical protein (WP_001046277.1)
			Hypothetical protein (WP_001024681.1)

			Hypothetical protein (WP_001238796.1)
			Hypothetical protein (WP_000504197.1)
			Hypothetical protein (WP_001090439.1)
			Hypothetical protein (WP_000479521.1)
			Hypothetical protein (WP_000913725.1)
			Hypothetical protein (WP_000941930.1)
			Hypothetical protein (WP_000420425.1)
			Hypothetical protein (WP_002015113.1)
			Hypothetical protein (WP_000038505.1)
			Hypothetical protein (WP_002015149.1)
			Hypothetical protein (WP_001101324.1)
			DNA topoisomerase I
			Hypothetical protein (WP_000355854.1)
			Hypothetical protein (WP_000386247.1)
			Hypothetical protein (WP_002011499.1)
			Hypothetical protein (WP_005028401.1)
			Hypothetical protein (WP_000743213.1)
			Hypothetical protein (AB994_RS01065)
			Hypothetical protein (WP_001445143.1)
			Phosphoglucosamine mutase
			Sulfonamide-resistant dihydropteroate synthase Sul2
			(2) Transposase ISVsa3
			Hypothetical protein (WP_000338945.1)
			Tyrosine recombinase
			Hypothetical protein (WP_001085037.1)
			Hypothetical protein (WP_000192313.1)
			Hypothetical protein (WP_000157684.1)
			Hypothetical protein (WP_000370460.1)
			Hypothetical protein (WP_001049486.1)
			DNA mismatch repair protein
			Hypothetical protein (WP_000608397.1)
			Hypothetical protein (WP_001257461.1)
			Hypothetical protein (WP_001061021.1)

Supplementary_Table_8

			Hypothetical protein (WP_000961970.1)
			Hypothetical protein (WP_002015155.1)
			Hypothetical protein (WP_000567442.1)
	LN_3B	4221	Transposase ISAba1
			Hypothetical protein (ACINNAV18_A0048)
			Sulfonamide-resistant Sul2
			Phosphoglucomutase/phosphomannomutase, alpha/beta/alpha domain I protein
			Transposase ISVsa3
			Transposase IS1008
	LN_18	3261	(2) Transposase ISAba125
			Transposase IS30
	LN_20	2174	(2) Transposase ISAba125

REFERENCE	QUERY	TOTAL_bp	FLUX_GENES
LN_8	LN_1	8997	Aminoglycoside O-phosphotransferase APH(3'')-Ib (strA)
			Aminoglycoside O-phosphotransferase APH(6)-Id (strB)
			Hypothetical protein (WP_005028401.1)
			Hypothetical protein (WP_000743213.1)
			Class 1 integron integrase IntI1
			Chloramphenicol efflux MFS transporter CmlA5
			Hypothetical protein (AB994_RS01200)
			Quaternary ammonium efflux SMR transporter QacE delta 1
			(2) Sulfonamide-resistant Sul1
			(2) Transposase ISAba1
	LN_3A	1692	Metal/formaldehyde-sensitive transcriptional repressor
			S-(hydroxymethyl)glutathione dehydrogenase

Supplementary_Table_8

	LN_7	95347	Hypothetical protein (ACINNAV18_A0003)
			Hypothetical protein (ACINNAV18_A0004)
			Hypothetical protein (ACINNAV18_A0005)
			Hypothetical protein (ACINNAV18_A0006)
			Hypothetical protein (ACINNAV18_A0007)
			Hypothetical protein (ACINNAV18_A0008)
			Hypothetical protein (ACINNAV18_A0009)
			Hypothetical protein (ACINNAV18_A0010)
			Hypothetical protein (ACINNAV18_A0011)
			Hypothetical protein (ACINNAV18_A0012)
			Hypothetical protein (ACINNAV18_A0013)
			Putative lipoprotein
			Hypothetical protein (ACINNAV18_A0015)
			Hypothetical protein (ACINNAV18_A0016)
			Hypothetical protein (ACINNAV18_A0017)
			Hypothetical protein (ACINNAV18_A0018)
			Hypothetical protein (ACINNAV18_A0019)
			Hypothetical protein (ACINNAV18_A0020)
			Hypothetical protein (ACINNAV18_A0021)
			Transcriptional regulator, TetR family
			Hypothetical protein (ACINNAV18_A0023)
			Hypothetical protein (ACINNAV18_A0024)
			Hypothetical protein (ACINNAV18_A0025)
			Hypothetical protein (ACINNAV18_A0026)
			Hypothetical protein (ACINNAV18_A0027)
			Hypothetical protein (ACINNAV18_A0028)
			Hypothetical protein (ACINNAV18_A0029)
			Transcriptional regulator, TetR family
			Hypothetical protein (ACINNAV18_A0031)
			Peptidase, M48 family
			GHKL domain protein
			Hypothetical protein (ACINNAV18_A0034)
			Hypothetical protein (ACINNAV18_A0035)

Supplementary_Table_8

			Hypothetical protein (ACINNAV18_A0036)
			Hypothetical protein (ACINNAV18_A0037)
			Phage integrase, N-terminal SAM domain protein
			Site-specific tyrosine recombinase XerC
			Hypothetical protein (ACINNAV18_A0040)
			Hypothetical protein (ACINNAV18_A0041)
			Hypothetical protein (ACINNAV18_A0042)
			Hypothetical protein (ACINNAV18_A0046)
			Transposase zinc-binding domain protein
			Hypothetical protein (ACINNAV18_A0055)
			Aminoglycoside resistence strB
			Aminoglycoside resistence strA
			Hypothetical protein (ACINNAV18_A0058)
			Hypothetical protein (ACINNAV18_A0059)
			Hypothetical protein (ACINNAV18_A0060)
			Putative DNA topoisomerase I
			Hypothetical protein (ACINNAV18_A0062)
			Hypothetical protein (ACINNAV18_A0063)
			IS66 C-terminal element
			Hypothetical protein (ACINNAV18_A0068)
			Hypothetical protein (ACINNAV18_A0069)
			PF07308 family protein
			Transglycosylase SLT domain protein
			Hypothetical protein (ACINNAV18_A0072)
			Hypothetical protein (ACINNAV18_A0073)
			Hypothetical protein (ACINNAV18_A0074)
			Hypothetical protein (ACINNAV18_A0075)
			Hypothetical protein (ACINNAV18_A0076)
			Hypothetical protein (ACINNAV18_A0077)
			Hypothetical protein (ACINNAV18_A0078)
			Integrase core domain protein
			S-(hydroxymethyl)glutathione dehydrogenase
			Metal-sensitive transcriptional repressor

Supplementary_Table_8

			Transposase, IS4 family
			Hypothetical protein (ACINNAV18_A0083)
			Hypothetical protein (ACINNAV18_A0084)
			Hypothetical protein (ACINNAV18_A0085)
			Hypothetical protein (ACINNAV18_A0086)
			Conjugal protein TraU
			Hypothetical protein (ACINNAV18_A0088)
			Hypothetical protein (ACINNAV18_A0089)
			Hypothetical protein (ACINNAV18_A0090)
			Hypothetical protein (ACINNAV18_A0091)
			Hypothetical protein (ACINNAV18_A0092)
			TrbI-like protein
			Conjugal protein TraN
			Conjugal protein TraM
			Hypothetical protein (ACINNAV18_A0096)
			Hypothetical protein (ACINNAV18_A0101)
			Putative lipoprotein
			Conjugal protein TraI
			Type II/IV secretion system domain protein
			StbA protein
			Hypothetical protein (ACINNAV18_A0106)
			Hypothetical protein (ACINNAV18_A0107)
			Hypothetical protein (ACINNAV18_A0108)
			Hypothetical protein (ACINNAV18_A0109)
			Hypothetical protein (ACINNAV18_A0110)
			Hypothetical protein (ACINNAV18_A0111)
			Hypothetical protein (ACINNAV18_A0112)
			Partition protein ParA
			Partition protein ParB
			ImpB/MucB/SamB family protein
			Peptidase S24-like protein
			Nucleotidyl transferase, PF08843 family (AbiEii/AbiGii toxin family protein)
			PF13338 domain protein

Supplementary_Table_8

			Hypothetical protein (ACINNAV18_A0119)
			Hypothetical protein (ACINNAV18_A0120)
			Hypothetical protein (ACINNAV18_A0121)
			Hypothetical protein (ACINNAV18_A0122)
			Peptidase, M23 family
			Hypothetical protein (ACINNAV18_A0124)
			Conjugal protein TraY
			Hypothetical protein (ACINNAV18_A0126)
			Hypothetical protein (ACINNAV18_A0127)
			Hypothetical protein (ACINNAV18_A0128)
			Hypothetical protein (ACINNAV18_A0131)
			Hypothetical protein (ACINNAV18_A0132)
			Hypothetical protein (ACINNAV18_A0133)
			Conjugal protein TraW
			Hypothetical protein (ACINNAV18_A0135)
			Hypothetical protein (ACINNAV18_A0136)
			Hypothetical protein (ACINNAV18_A0137)
			Hypothetical protein (ACINNAV18_A0138)
			Hypothetical protein (ACINNAV18_A0139)
			Hypothetical protein (ACINNAV18_A0140)
			Helicase C-like protein
			Hypothetical protein (AB994_RS00210)
			Hypothetical protein (WP_000493808.1)
			Hypothetical protein (WP_000369329.1)
			Hypothetical protein (WP_002038214.1)
			Hypothetical protein (WP_001101148.1)
			Hypothetical protein (WP_000064550.1)
			Hypothetical protein (WP_000568115.1)
			Hypothetical protein (WP_001075210.1)
			Hypothetical protein (WP_000700025.1)
			Hypothetical protein (WP_001226062.1)
			Hypothetical protein (WP_000202450.1)
			Hypothetical protein (WP_000640998.1)

Supplementary_Table_8

			Hypothetical protein (WP_000481946.1)
			Hypothetical protein (WP_002011618.1)
			Hypothetical protein (WP_000071965.1)
			Hypothetical protein (AB994_RS00290)
			Hypothetical protein (WP_000824500.1)
			H-NS histone
			Hypothetical protein (WP_001102765.1)
			Hypothetical protein (WP_000182799.1)
			Hypothetical protein (WP_000594604.1)
			Helicase
			Hypothetical protein (WP_002011620.1)
			Hypothetical protein (WP_002016363.1)
			Hypothetical protein (WP_000445287.1)
			Hypothetical protein (WP_002011578.1)
			Hypothetical protein (WP_001211405.1)
			Hypothetical protein (WP_000730798.1)
			Hypothetical protein (WP_000871698.1)
			Hypothetical protein (WP_000773909.1)
			Hypothetical protein (WP_000994248.1)
			Hypothetical protein (WP_000236940.1)
			Hypothetical protein (WP_002015162.1)
			Hypothetical protein (WP_001217963.1)
			Hypothetical protein (WP_000123889.1)
			Hypothetical protein (WP_000287492.1)
			Hypothetical protein (WP_000069170.1)
			Hypothetical protein (WP_000956387.1)
			Hypothetical protein (WP_000733607.1)
			Hypothetical protein (WP_002011623.1)
			Hypothetical protein (WP_000343986.1)
			Hypothetical protein (WP_002011569.1)
			Hypothetical protein (WP_000271057.1)
			DNA polymerase V
			DNA polymerase V subunit UmuC

			Hypothetical protein (WP_001177226.1)
			Hypothetical protein (WP_001085267.1)
			Hypothetical protein (WP_000444265.1)
			Hypothetical protein (WP_000765384.1)
			Hypothetical protein (WP_000851072.1)
			Hypothetical protein (WP_000069415.1)
			Hypothetical protein (WP_001135704.1)
			Hypothetical protein (WP_000389973.1)
			Hypothetical protein (WP_001103687.1)
			Hypothetical protein (WP_000706892.1)
			Hypothetical protein (WP_000643578.1)
			Hypothetical protein (WP_000665168.1)
			Hypothetical protein (WP_000064221.1)
			Hypothetical protein (WP_000447535.1)
			Hypothetical protein (WP_000036098.1)
			Hypothetical protein (WP_000608872.1)
			Hypothetical protein (WP_000573069.1)
			Hypothetical protein (WP_001009075.1)
			Hypothetical protein (WP_002015136.1)
			Hypothetical protein (WP_000931461.1)
			Hypothetical protein (WP_004895603.1)
			(2) Transposase IS1008
			Hypothetical protein (WP_000644488.1)
			Hypothetical protein (WP_001046277.1)
			Hypothetical protein (WP_001024681.1)
			Hypothetical protein (WP_001238796.1)
			Hypothetical protein (WP_000504197.1)
			Hypothetical protein (WP_001090439.1)
			Hypothetical protein (WP_000479521.1)
			Hypothetical protein (WP_000913725.1)
			Hypothetical protein (WP_000941930.1)
			Hypothetical protein (WP_000420425.1)
			Hypothetical protein (WP_002015113.1)

Supplementary_Table_8

			Hypothetical protein (WP_000038505.1)
			Hypothetical protein (WP_002015149.1)
			Hypothetical protein (WP_001101324.1)
			DNA topoisomerase I
			Hypothetical protein (WP_000355854.1)
			Hypothetical protein (WP_000386247.1)
			Hypothetical protein (WP_002011499.1)
			Hypothetical protein (WP_005028401.1)
			Hypothetical protein (WP_000743213.1)
			Hypothetical protein (AB994_RS01065)
			Hypothetical protein (WP_001445143.1)
			Phosphoglucosamine mutase
			Sulfonamide-resistant dihydropteroate synthase Sul2
			(2) Transposase ISVsa3
			Hypothetical protein (WP_000338945.1)
			Tyrosine recombinase
			Hypothetical protein (WP_001085037.1)
			Hypothetical protein (WP_000192313.1)
			Hypothetical protein (WP_000157684.1)
			Hypothetical protein (WP_000370460.1)
			Hypothetical protein (WP_001049486.1)
			DNA mismatch repair protein
			Hypothetical protein (WP_000608397.1)
			Hypothetical protein (WP_001257461.1)
			Hypothetical protein (WP_001061021.1)
			Hypothetical protein (WP_000961970.1)
			Hypothetical protein (WP_002015155.1)
			Hypothetical protein (WP_000567442.1)
	LN_10	22559	Class 1 integron integrase IntI1
			(2) Transposase IS26
			Hypothetical protein (WP_001189351.1)
			Mph(E) family macrolide 2'-phosphotransferase

Supplementary_Table_8

			ABC-F type ribosomal protection protein Msr(E)
			Transposase ISEc29
			Hypothetical protein (WP_002026779.1)
			Hypothetical protein (ABZJ_RS19730)
			ArmA family 16S rRNA (guanine(1405)-N(7))-methyltransferase
			Hypothetical protein (WP_012512981.1)
			Hypothetical protein (WP_000520337.1)
			IS5/IS1182 family transposase pseudo
			Transposase IS91
			Sulfonamide-resistant dihydropteroate synthase Sul1
			Quaternary ammonium compound efflux SMR transporter QacE delta
			Hypothetical protein (WP_000683476.1)
			Transposase IS15DIV
			Hypothetical protein (WP_002038995.1)
			Hypothetical protein (AB994_RS01270)
			Hypothetical protein (WP_004786457.1)
			RepB family plasmid replication initiator protein
	LN_11	3532	Transposase ISAba34
			Transposase IS15DIV
			Transposase IS26
			Transposase ISAba2
	LN_3B	4634	(2) Transposase IS6
			Transposase ISVsa3
			Hypothetical protein (WP_001445143.1)
			Phosphoglucosamine mutase
			Sulfonamide-resistant dihydropteroate synthase Sul2
			Nucleotide pyrophosphohydrolase

REFERENCE	QUERY	TOTAL_bp	FLUX_GENES

Supplementary_Table_8

LN_10	LN_1	2177	Class 1 integron integrase IntI1
			Sulfonamide-resistant dihydropteroate synthase Sul1
			Quaternary ammonium compound efflux SMR transporter QacE delta
	LN_8	22559	Class 1 integron integrase IntI1
			(2) Transposase IS26
			Hypothetical protein (WP_001189351.1)
			Mph(E) family macrolide 2'-phosphotransferase
			ABC-F type ribosomal protection protein Msr(E)
			Transposase ISEc29
			Hypothetical protein (WP_002026779.1)
			Hypothetical protein (ABZJ_RS19730)
			ArmA family 16S rRNA (guanine(1405)-N(7))-methyltransferase
			Hypothetical protein (WP_012512981.1)
			Hypothetical protein (WP_000520337.1)
			IS5/IS1182 family transposase pseudo
			Transposase IS91
			Sulfonamide-resistant dihydropteroate synthase Sul1
			Quaternary ammonium compound efflux SMR transporter QacE delta
			Hypothetical protein (WP_000683476.1)
			Transposase IS15DIV
			Hypothetical protein (WP_002038995.1)
			Hypothetical protein (AB994_RS01270)
			Hypothetical protein (WP_004786457.1)
			RepB family plasmid replication initiator protein
	LN_11	2112	(3) Transposase IS6

REFERENCE	QUERY	TOTAL_bp	FLUX_GENES
LN_11	LN_2	3625	RepB protein
			DNA-binding protein

Supplementary_Table_8

			Hypothetical protein (WP_000504218.1)
			Hypothetical protein (WP_001014301.1)
			Hypothetical protein (WP_012576333.1)
	LN_8	3532	Transposase ISAba34
			Transposase IS15DIV
			Transposase IS26
			Transposase ISAba2
	LN_10	2112	(3) Transposase IS6
	LN_13	1125	Hypothetical protein (WP_000504218.1)
			Hypothetical protein (WP_001014301.1)
	LN_19	1248	Hypothetical protein (WP_012576333.1)
			Hypothetical protein (WP_000504218.1)
			Hypothetical protein (WP_001014302.1)
			Hypothetical protein (WP_005265294.1)
			Hypothetical protein (WP_005173935.1)
	LN_20	7511	DNA-binding protein
			Hypothetical protein (WP_004895776.1)
			Carbapenem-hydrolyzing class D beta-lactamase OXA-58
			(2) Transposase ISAba3
			AraC family transcriptional regulator
			Carbapenem-hydrolyzing class D beta-lactamase OXA-58
			AraC family transcriptional regulator
			Hypothetical protein (WP_012576333.1)
			Hypothetical protein (WP_000504218.1)
			Hypothetical protein (WP_001014301.1)

Supplementary_Table_8

REFERENCE	QUERY	TOTAL_bp	FLUX_GENES
LN_12	LN_17	1501	Hypothetical protein (WP_005000600.1)
			Mobilization protein MOBQ

REFERENCE	QUERY	TOTAL_bp	FLUX_GENES
LN_13	LN_2	5544	Toxin-Antitoxin system spITA (COG3514)
			Toxin-Antitoxin system spITA (DUF497)
			TonB dependent receptor
			Hypothetical protein (WP_004781077.1)
			Hypothetical protein (WP_000504218.1)
			Hypothetical protein (WP_001014301.1)
	LN_11	1125	Hypothetical protein (WP_000504218.1)
			Hypothetical protein (WP_001014301.1)
	LN_15	4443	Toxin-Antitoxin system spITA (COG3514)
			Toxin-Antitoxin system spITA (DUF497)
			TonB dependent receptor
			Hypothetical protein (WP_004781077.1)
	LN_19	1307	Hypothetical protein (WP_004781077.1)
			Hypothetical protein (WP_000504218.1)
			Hypothetical protein (WP_001014301.1)
			Hypothetical protein (WP_005265294.1)
	LN_20	2440	DNA-binding protein
			Hypothetical protein (WP_000504218.1)
			Hypothetical protein (WP_001014301.1)

REFERENCE	QUERY	TOTAL_bp	FLUX_GENES

Supplementary_Table_8

LN_14	LN_17	1086	Carbapenemase OXA-24

REFERENCE	QUERY	TOTAL_bp	FLUX_GENES
LN_15	LN_2	4820	Toxin-Antitoxin system spITA (COG3514)
			Toxin-Antitoxin system spITA (DUF497)
			TonB dependent receptor
			Hypothetical protein (WP_004781077.1)
			Septicolysin
	LN_13	4443	Toxin-Antitoxin system spITA (COG3514)
			Toxin-Antitoxin system spITA (DUF497)
			TonB dependent receptor
			Hypothetical protein (WP_004781077.1)

REFERENCE	QUERY	TOTAL_bp	FLUX_GENES
LN_17	LN_6	1410	Beta-lactamase OXA-72
	LN_12	1501	Hypothetical protein (WP_005000600.1)
			Mobilization protein MOBQ
	LN_14	1086	Carbapenemase OXA-24

REFERENCE	QUERY	TOTAL_bp	FLUX_GENES
LN_18	LN_1	3260	(2) Transposase ISAba125
			Transposase IS30
	LN_7	3261	(2) Transposase ISAba125
			Transposase IS30

Supplementary_Table_8

	LN_20	3261	(3) Transposase ISAba125

REFERENCE	QUERY	TOTAL_bp	FLUX_GENES
LN_19	LN_2	1920	Hypothetical protein (WP_000504218.1)
			Hypothetical protein (WP_001014301.1)
			Hypothetical protein (WP_012576333.1)
			Hypothetical protein (WP_005265294.1)
			Hypothetical protein (WP_005173935.1)
	LN_11	1248	Hypothetical protein (WP_012576333.1)
			Hypothetical protein (WP_000504218.1)
			Hypothetical protein (WP_001014302.1)
			Hypothetical protein (WP_005265294.1)
			Hypothetical protein (WP_005173935.1)
	LN_13	1307	Hypothetical protein (WP_004781077.1)
			Hypothetical protein (WP_000504218.1)
			Hypothetical protein (WP_001014301.1)
			Hypothetical protein (WP_005265294.1)
	LN_20	1832	Hypothetical protein (WP_000504218.1)
			Hypothetical protein (WP_005265294.1)
			Hypothetical protein (WP_005173935.1)
			Hypothetical protein (WP_001014301.1)

REFERENCE	QUERY	TOTAL_bp	FLUX_GENES
LN_20	LN_1	2193	(2) Transposase ISAba125
	LN_2	2855	DNA-binding protein
			Hypothetical protein (WP_000504218.1)

Supplementary_Table_8

			Hypothetical protein (WP_001014301.1)
			Hypothetical protein (WP_012576333.1)
	LN_7	2174	(2) Transposase ISAba125
	LN_11	7511	DNA-binding protein
			Hypothetical protein (WP_004895776.1)
			Carbapenem-hydrolyzing class D beta-lactamase OXA-58
			(2) Transposase ISAba3
			AraC family transcriptional regulator
			Carbapenem-hydrolyzing class D beta-lactamase OXA-58
			AraC family transcriptional regulator
			Hypothetical protein (WP_012576333.1)
			Hypothetical protein (WP_000504218.1)
			Hypothetical protein (WP_001014301.1)
	LN_13	2440	DNA-binding protein
			Hypothetical protein (WP_000504218.1)
			Hypothetical protein (WP_001014301.1)
	LN_18	3261	(3) Transposase ISAba125
	LN_19	1832	Hypothetical protein (WP_000504218.1)
			Hypothetical protein (WP_005265294.1)
			Hypothetical protein (WP_005173935.1)
			Hypothetical protein (WP_001014301.1)

Supplementary_Table_9. Replication proteins of *A. baumannii* plasmids which are identical in sequence to replication proteins in the genomes of other *Acinetobacter* species and in other genera. **LN**, lineage. **ID_REP**, GenBank accession number of the Rep proteins analyzed in this work. Column marked with ***ACINETOBACTER***, show that the Rep protein have identical matches within this genus. *, label a small protein annotated as replication protein with now homologues. Column label as **OUT_OF_*ACINETOBACTER*** show in which genomes the Rep protein has identical matches. ** label a HTH proteins mistakenly annotated as replication protein in GenBank. Plasmids in bold have more than one Rep protein.

PLASMID	LN	ID_REP	GR	*ACINETOBACTER*	OUT_OF_*ACINETOBACTER*
p1ABSDF	orphan	CAP02936.1	GR1	*A. baumannii*	
pAB2	orphan	WP_001205343.1	GR2	*A. indicus, A. pittii*	*Enterococcus faecium, Klebsiella pneumonie , Providencia rettgeri,*
pORAB01-3	orphan	WP_001205341.1	GR2	*A. baumannii, A. johnsonii, A. pittii, A. soli*	
p1ABST78	orphan	WP_001205343.1	GR2	*A. baumannii, A. indicus, A. pittii,*	*Enterococcus faecium, Klebsiella pneumonie , Providencia rettgeri,*
plasmid unnamed2	orphan	WP_060853654.1	GR2	*A. baumannii,*	
p1ABTCDC0715	LN_2	ADX94286.1	GR2	*A. baumannii*	
pAC12	LN_2	AHX30527.1	GR2	*A. baumannii*	*Klebsiella pneumonie*
pAC30a	LN_2	AHX67213.1	GR2	*A. baumannii*	*Klebsiella pneumonie*
plasmid unnamed2	LN_2	AQU58924.1	GR2	*A. baumannii*	*Klebsiella pneumonie*
plasmid unnamed2	LN_2	AQU58933.1	GR2	*A. baumannii*	*Klebsiella pneumonie*
**pACICU1/ p2ABST2**	LN_11	WP_001205343.1	GR2	*A. baumannii, A. indicus, A. pittii*	*Klebsiella pneumonie, Enterococcus faecium, Providencia rettgeri*
pHWBA8_1	orphan	WP_000534216.1	GR3	*A. baumannii,*	*Providencia stuartii, Escherichia coli, Citrobacter freundii, Klebsiella pneumoniae, Proteus mirabilis, Enterobacter sp., Salmonella enterica, Klebsiella quasipneumoniae, Shewanella putrefaciens, Raoultella planticola, Klebsiella oxytoca, Klebsiella michiganensis, Vibrio alginolyticus, Vibrio parahaemolyticus, Raoultella ornithinolytica, Enterobacter hormaechei, Vibrio cholerae, Pseudomonas putida, Pseudomonas aeruginosa, Enterobacter cloacae,*
pAF-401	orphan	WP_000845851.1	GR3	*A. baumannii, A. pittii, A. nosocomialis*	*Methylococcus capsulatus, K. pneumoniae,*

pB11911/pMDR-ZJ06	LN_8/ LN_10	WP_000534216.1	GR3	*A. baumannii*	*Klebsiella pneumonie, Providencia stuartii, Escherichia coli, Citrobacter freundii, Proteus mirabilis, Enterobacter sp., Salmonella enterica, Klebsiella quasipneumoniae, Shewanella putrefaciens, Raoultella planticola, Klebsiella oxytoca, Pseudomonas aeruginosa, Vibrio alginolyticus, Vibrio parahaemolyticus, Raoultella ornithinolytica, Enterobacter cloacae, Klebsiella aerogenes, Enterobacter hormaechei*
pD1279779/ pABLAC1	LN_17/ LN_22	WP_000845851.1	GR3	*A. baumannii, A. pittii*	*Klebsiella pneumonie , Methylococcus capsulatus, Sphingobium sp.*
pIOMTU433	LN_8	WP_042634593.1	GR3	*A. baumannii*	*Klebsiella pneumonie, Vibrio cholerae, Klebsiella quasipneumoniae, Enterobacter kobei*
pAC29b	orphan	WP_002001291.1	GR6	*A. baumannii,*	*Klebsiella pneumonie*
p2ABTCDC0715	LN_1	ADX94329.1	GR6	*A. baumannii*	*Klebsiella pneumonie*
pAC30c	LN_1	AHX67284.1	GR6	*A. baumannii*	*Klebsiella pneumonie*
plasmid unnamed1	LN_1	AQU58831.1	GR6	*A. baumannii*	
pA85-3	LN_1	WP_002001291.1	GR6	*A. baumannii,*	*Klebsiella pneumonie*
pAba7847b	LN_1	WP_002031424.1	GR6	*A. baumannii, A. pitti, A. nosocomilis*	
pSSA12_1	LN_1	WP_014462889.1	GR6	*A. baumannii*	
pAB04-2	LN_1	WP_079265452.1	GR6	*A. baumannii*	
plasmid	LN_1	WP_079280812.1	GR6	*A. baumannii*	
pAC29b	orphan	WP_002001291.1	GR6	*A. baumannii,*	*Klebsiella pneumoniae,*
p2ABTCDC0715	LN_1	ADX94329.1	GR6	*A. baumannii*	*Klebsiella pneumonie*
pAC30c	LN_1	AHX67284.1	GR6	*A. baumannii*	*Klebsiella pneumonie*
plasmid unnamed1	LN_1	AQU58831.1	GR6	*A. baumannii*	
pA85-3	LN_1	WP_002001291.1	GR6	*A. baumannii,*	*Klebsiella pneumonie*
pAba7847b	LN_1	WP_002031424.1	GR6	*A. baumannii, A. pitti, A. nosocomilis*	
pSSA12_1	LN_1	WP_014462889.1	GR6	*A. baumannii*	
pAB04-2	LN_1	WP_079265452.1	GR6	*A. baumannii*	

plasmid	LN_1	WP_079280812.1	GR6	*A. baumannii*	
p3ABSDF	orphan	CAP02976.1 /WP_000798284.1	GR7	*A. baumannii*	
pAba10324a	orphan	WP_078377890.1	GR7	*A. baumannii,*	
p3ABSDF	orphan	WP_000798284.1	GR7	*A. baumannii*	
pAba10324a	orphan	WP_078377890.1	GR7	*A. baumannii,*	
pNaval18-8.4	orphan	EJP48482.1	GR8	*A. baumannii*	*Klebsiella oxytoca*
plasmid:2	orphan	WP_001292329.1	GR8	*A. baumannii, A gerneri, A. indicus*	*Salmonella enterica*
p6200-9.327kb	orphan	WP_038350249.1	GR8	*A. baumannii,*	
p3ABSDF	orphan	CAP02983.1	GR9	*A. baumannii, A. schindleri*	
**pACICU1/ p2ABST2**	LN_11	WP_000845976.1	GR10	*A. baumannii*	
p1ABAYE	LN_12	WP_001031297.1	GR11	*A. baumannii, A. pittii, A. nosocomialis, A. wuhouensis, A. johnsonii, A. colistiniresistens*	*Nakamurella silvestris*
p2ABSDF	orphan	CAP02944.1/ WP_001038666.1	GR12	*A. baumannii, A. haemolyticus, A. variabilis*	
plasmid unnamed 2	orphan	WP_001038666.1	GR12	*A. baumannii, A. haemolyticus, A. variabilis*	
pABUH3a-8.2	LN_18	WP_005804946.1	GR12	*A. baumannii, A. nosocomilais, A. pittii, A. haemolyticus, A. bereziniae, A. seifertii, A. proteolyticus*	
p3ABAYE	orphan	WP_000064928.1	GR13	*A. baumaii, A. nosocomialis, A. pittii,*	
p4ABAYE	LN_9	WP_001180321.1	GR14	*A. baumannii*	
pA85-1	LN_9	WP_031943495.1	GR14	*A. baumannii*	
p3ABSDF	orphan	CAP02992.1	GR15	*A. baumannii*	
pNaval17-13	LN_20	WP_000987942.1	GR15	*A. baumannii,*	
pA85-1a	LN_23	WP_088631515.1	GR16	*A. baumannii*	

pAB1	orphan	ABO13850.1	GR17**	*A. baumannii,*	
p2ABSDF	orphan	CAP02966.1	GR18	*A. baumannii*	
pNaval18-7.0	orphan	EJP48327.1	GR20	*A. baumannii, A. bereziniae,*	
pABUH6b-10	orphan	WP_001208778.1	GR20	*A. baumannii, A. nosocomialis, A. pittii*	
pIS123-12	orphan	WP_001208779.1	GR20	*A. baumannii, A. pittii, A. llwoffii*	*Klebsiella pneumoniae, Neisseria meningitidis*
pCS01C	LN_13	WP_001208776.1	GR20	*A. baumannii*	
pOIFC032-101	orphan	WP_000818857.1	GR24	*A. baumannii, A. pittii, A. indicus,*	
ZW85p2	LN14	WP_000818856.1	GR24	*A. baumannii, A. pitti, A. nosocomilis*	*Klebsiella pneumonie*
p6200-114.848kb	LN_3	WP_000818857.1	GR24	*A. baumannii, A. pitti,*	
pABTJ1	LN_5	WP_000633173.1	GR25	*A. baumannii*	
pAba7835a	orphan	WP_063558588.1	GR26	*A. baumannii,*	
pAba7847a	LN_21	WP_063558588.1	GR26	*A. baumannii*	
pABUH2b-5.4	LN15	WP_004282236.1	GR27	*A. baumannii, A. bereziniae, A. lwoffii, A. soli*	*Prolinoborus fasciculus, Neisseria meningitidis, Pseudomonas sp.*
pAba810CPa	LN18	WP_004729701.1	GR27	*A. baumannii, A. lwoffii*	*Prolinoborus fasciculus*
pABUH2a-5.6	LN_15	WP_032021082.1	GR27	*A. baumannii*	
pIS123-18	orphan	WP_000185725.1	GR28	*A. baumannii,*	
pNaval81-26	orphan	WP_000185726.1	GR28	*A. baumannii,*	
pAba10042a	LN_6	WP_012780181.1	GR29	*A. baumannii, A. calcoaceticus, A. pittii, A. soli, A. wuhouensis*	
pOIFC143-2.3	orphan	WP_000711927.1	GR30	*A. baumannii,*	*Gallaecimonas pentaromativorans*
pABUH5-114	orphan	WP_000095317.1	GR31	*A. baumannii*	
pD36-4	orphan	WP_000140303.1	GR32	*A. baumannii*	
pKBN10P02143	orphan	WP_059273206.1	GR33	*A. baumannii,*	

pAC30b	orphan	AHX67224.1	*	*A. baumannii,*	
pAB3	orphan	WP_000743064.1	GR34	*A. baumannii, A. lwoffii, A. bereziniae,*	*Shigella flexneri, Escherichia coli, Stenotrophomonas maltophilia, Gallibacterium anatis, Shigella dysenteriae, Nitrosomonas eutropha, Achromobacter xylosoxidans, Sphingopyxis granuli, Oblitimonas alkaliphila, Morganella morganii, Providencia stuartii, Bordetella bronchiseptica, Achromobacter denitrificans, Alcaligenes faecalis, Melaminivora sp., Shigella sonnei, Citrobacter freundii, Enterobacter cloacae, Paracoccus alcaliphilus, Mycobacteroides abscessus, Shigella sonnei, Citrobacter koseri, Klebsiella pneumoniae, Oligella urethralis, Oligella ureolytica, Aeromonas hydrophila, Gallibacterium anatis, Shinella sp., Comamonas granuli, Pseudomonas monteilii, Thauera butanivorans, Sphingobium faniae, Thauera butanivorans, Enterobacter kobei, Ignatzschineria sp., Pusillimonas sp., Ignatzschineria cameli,*

Supplementary_Table_10. GenBank accession numbers of Mexican isolates and their Sequence type following Bartual (ST_B) and Pasteur (ST_P) schemes.

STRAIN	ST_B	ST_P	ACCESSION NUMBER
7847	208	-----	NZ_CP023031.1, CP023032.1, CP023033.1
7835	227	422	CP033243.1, CP033244.1, CP033245.1
9102	231	1	CP023029.1, CP023030.1
5845	417	2	NZ_CP023034.1, CP023035.1
10042	473	2	NZ_CP023026.1, CP023027.1, CP023028.1
11510	758	156	NZ_CP018861.2 NZ_CP018862.2
810CP	758	156	NZ_CP026338.1, CP026339.1, CP026340.1
10324	771	10	NZ_CP023022.1, CP023023.1, CP023024.1, CP023025.1
9201	934	422	NZ_CP023020.1, CP023021.1
3207	1321	422	NZ_CP015364.1, NZ_CP015365.1, NZ_CP015366.1
