# Supplementary material for: Structure and Evolution of Acinetobacter baumannii Plasmids
Source: Front Microbiol. 2020 Jun 18;11:1283. doi: 10.3389/fmicb.2020.01283 (PMC7315799; doi:10.3389/fmicb.2020.01283)
Supplement: MATERIAL S1 — List in fasta format of genes encoding representative replication proteins of each one of the GR homology groups. [file Data_Sheet_3.zip › Supplementary Materials S2.docx]

Rep Protein sequences of the representative members of each GR homology group

>GR1_CU468231.1_ABSDF_p10001_NCBI CAP02936.1 putative replication protein

MKKICVLMKKELVVKDNALINASYNLDLSEQRLILLAILEARQSNTPNDKDLTIHAESYINHFNVHRNTAYKVLKDACKSLFDRRFSYQKLTQKGNIENVISRWVQRISYVENEALVRIKFSDDVVPLITNLEKHFTSYELEQVSSLTSVYAIRLYELLIAWRSTGKVILVELEELRLKLGIESHEYKRMGQFKEKVLHLAIDQINKYTDIKAEYEQHKRGRSIIGFSFKFKQKQQPQKADSKRAPNTPDFFVKMTDAQRHLFANKMSEMPEMSKYSQGTESYQQFAIRIADMLLEPEKFRELYPILEKAGFKG

>GR2_NC_010605.1_ACICU_RS18410_NCBI WP_001205343.1 RepB family plasmid replication initiator protein

MRDLVVKDNALINASYNLDLVEQRLILLAIVEARESGKGINANDPLEVHADSYINQFGVHRNTAYQALKDACKDLFARQFSYQEKKANGNIRNVMSRWVSQIAYNDNEATVDLIFAPAVVPFITRLEEQFTKYELQQVSSLSSAYAIRLYELLIQWRSTGKTPTIELQEFRKKLGVLDNEYLRMAHLKERVLELSIKQINEHTDITVKYEQHKRGRSISGFSFTFKQKKKDSPSIERDPNTLELFSKMTDAQRHMFANKLSELPEMGRYSQGTESYQQFAVRIAEMLQDPAQFKELYPYLKKVGYMPSNKKDTVNG

>GR3_1_GU978997.1_p203_repA_NCBI ADM89092.1 plasmid replication protein Aci 3

MKTELIVKDNALINASYNLDLVEQRLILLAIVEARESGKGINANDPLTVHAESYINQFGVHRNTAYQALKDACDDLFARQFSYQSLSEKGNIINHKSRWVSEVAYIDNEAVVRLIFAPAIVPLITRLEEQFTKYEIQQISNLTSAYAVRLYEILIAWRSTGKTPLITLSDFRQKIGVLDTEYKRMYDFKKYVLDIALKQVNEHTDITVKVEQHKTGRSITGFSFSFKQKKSVTKSAKSIGVSEDITITLTDAQRYSFASKLSELPEMGKLSQGTESYEQFAVRIADMLKQPEKLKELTPLLRKVGFQ

>GR3_2_GU978996.1_Abap736_repA_NCBI ADM89091.1 plasmid replication protein Aci 7

MKTELIVKDNALINASYNLDLVEQRLILLAILEARESGKGINANDPLTVHAESYINQFGVHRNTAYQALKDACDDLFVRQFSYQSLSEKGNVINHKSRWVSEVAYIDNEAVVRLIFAPAIVPLITRLEEQFTKYEIQQISNLTSAYAVRLYEILIAWRSTGKTPLITLYDFRQKIGVLDTEYKRMYDFKKYVLDIALKQVNEHTDITVKVEQHKTGRSITGFSFSFKQKKSATQSVGSKRDPNTLDPFSTMTDKQRHLFASKLSELPEMSKYSQGTESYQQFAVRIAGMLQDTERFREINSFAQKK

>GR4_GU978998.1_p844_repA_NCBI ADM89093.1 plasmid replication protein Aci 4

MRDLVVKDNALINASYNLDLVEQRLILLAIVEARDSGRGINANDPLEVHAESYVNQFNVARQTAYQALKDACKDLFVRQFSYQEINKRGNVENVLSRWVSEIRYIDDEATVKLIFAPAIVPLITRLEEQFTKYELQQISNLSSAYAVRLYELLIAWRSTGQTPIIELAEFRKKIGVLDDEYTRMGNFKDRVLNLAIAQINEHTDIKVQCQQHKKGRNISGFSFTFKQKKVVIANNKKQTTLEIFSKFTDAQRHFFANKLSELPEMNKYSQGTESYSQFAVRISEMLKDLQKFEELLPYLEKVGFNAK

>GR5_GU978999.1_p537_repA_NCBI ADM89094.1 plasmid replication protein Aci 5

MRDLVVKDNALINASYNLDLVEQRLILLAIVEARESGKGINANNPLEVHAESYINQFNVARQTAYQALKDASKDLFARQFSYQEMNKRGNIENVLSRWVSEIRYIDAEATVKLIFAPAIVPLITKLEEQFTKYELQQVSNLSSAYAVRLYELLIAWRSTGQTPVIELEEFRKKIGVLDDEYTRMGNFKDRVLHLAIDQVNEFTDITVKYEQHKKGRSIYGFSFSFKQKKNVNKPNLEARDQNTLDIFTKLTDAQRHLFANKLSELPEMSKYSQGTESYPQFAVRIAEMLLDAEKFKELYPYLVKVGFQTK

>GR6_NC_010606.1_ACICU_RS18590_NCBI WP_000217892.1 Frameshifted Plasmid replicase

MENLYFFNERNKYLKDIKYLIFLLLQNSIEDASIEHQKLISMSVRILNNGVLYKMDFSSLISFKNELINLNKELNNGNK

>GR7_CU468233.1_ABSDF_p30002_NCBI CAP02976.1 DNA replication protein

MKNSLVVKDNALINASYNLELTEQRLIMLAIINARESGQGITADSKLEIHASDYAKLFNVSIDASYKALKEAVNNLFNRQFSYTAEYKRTGKTGVVRSRWVSRIFYVDDLALLEITFAPDVVPLVTRLEEHFTSYQAKQVAHLTSKYATRLYELLIAWREVGKVPQIEISTFRNRLGLLENEYTAMSDFKKRVLEPSIKQINEHTDITVTYEQHKKGRLISGFSFKLKQKQQPKIEVKRDPNTPDFFVKMTDAQRHLFANKMSEMPDMSKYSQGTESYQQFAIRIADMLLEPEKFRELYPCLEKAGFQPA

>GR8_1_GU979000.1_p11921_repA_NCBI ADM89095.1 plasmid replication protein Aci 8

MSELIVKDNALIQASYTLDTVEQRLILLAIAEARETGHGINENSLLQVHASSYINTFNVEKHTAYTVLRDASKSLFDRYVTYHDINPKTDKDRSFHCRWVDKIGYEPQSGIVFLRFTQDIVPLITRLEENFTKYELQQVSRLTSSYAIRLYELLIQWGSRGKTPTFDLHVFRNRLGVEDGQYKTMCNFKQFVLDFALKQINQFTDIIAKYEQHKSGRKITGFSFTFKFKNNKNVKEKLVEKTEFYKLTESQLDLFAKKLAHLPELGHLADEGMSYEEFYSKLKSILKDPEQQKKLVPYFEKAGLNPK

>GR8_2_AY541809.1_AY541809_repM_NCBI AAT09649.1 DNA replication protein

MSELIVKDNALIQASYTLDTVEQRLILLAIAEARETGHGITENSLLEVHASSYINTFNVEKHTAYTVLREASKSLFDRYVTYHDINPKTGKDRSFHCRWVDKIGYESQSGIIFLRFTQDIVPLITRLEENFTKYELQQVSRLSSSYAIRLYELLIQWRSAGKTPLFDLSIFRQQLGVKPHQYKTMSNFKTYVLDFALKQVNELTDITAKYEQHKKGRSISGFSFTFKQKKMSNLPIKNKRDPDTIDIFSKMTDAQRHLFSHKLSELPEMGKYSHGTESYPQFAVRIAEMLQNPEKFKELYPYLQKVGFKAA

>GR9_CU468233.1_ABSDF_p30009_NCBI CAP02983.1 DNA replication protein

MANDLVIKNNALIDASYTLSLVEQRLIGLALVKANNQHQEITSDTVLTIHAGEYAQQFNVDGSVAYRALKEASERLFLRYFSYTLYGLDFGKEYTLKRPKKLKDGDIPTIMKSRWVQKVGYTESEGLLHFQLTSDVVRLVANSKEYFTSYYLSQTTEFTSTYATRLFELLMKWKNVGHIPFIEIEQLRGQLGVEPKQYKIISNFKLRVLDVAVEQVNQYSDYKIEYEQHKQGRTITGFSFKFQPKATKTKKIESRRDPNTPDFFIKMTDAQRHLFANKMSEMPEMIKYSQGTESYQQFTIRIADMLLQPEKFRELYPILEKAGFKG

>GR10_NC_010605.1_ACICU_RS18440_NCBI WP_000845976.1 RepB family plasmid replication initiator protein

MKTELVVKDNALINASYNLELAEQRLILLSIVKARETGRGITSDSRLEVHASDYMKQFNVEKSAAYEVLKSASESLFNRYFSYKEQRHDGTEFVVKSRWVSRVAYAPNVAILEVTFAPDVVPLITRLEQHFTSYQLKQVSQLTSKYAIRLYEMLIAWRNVGKCSFELINLRDSLGIASDEYKQMGHFKSRVLDASIAQINEYTDIKVTYEQQKNGRTISGFTFKLKPKQVQQEITILDTKASLIPSDLTPNQRVTFASKLSKLQELGGKAEPGEEVEAFAKRIELWLEDEKKLKMLTPFLYQVGFKKAKPKKVSQ

>GR11_NC_010401.1_ABAYE_RS00005_NCBI WP_001031297.1 RepB family plasmid replication initiator protein

MNKENSYDKSYPVTTMAIQNKVTECFKSMSVDEKRILIMASPIARNVDASEQDQILISAQQFADDCGIKVNSAYKQIENASKKLVDRSFSYVNDRGKKVYSNWVIDATYEDAGISLRFTSIVLVMLKILDKYNPYTRYKKDVVLKLKKDYSIDFYHLAKKNQAKNGFELTLDEMFTEFGLPESYRDLRNLKRRVLKSSLDEINEFTDVTVDYSPVKKGRSVVGFKFTVKEKSKPKLIAPERDPKTIDMFCNLSDAQINKYSAILSKLSELSDLSNFQDYPSFALWISGILRDPKSVREETAKRIFKALHSKTDFKP

>GR12_CU468232.1_ABSDF_p20001_NCBI CAP02944.1 putative replication protein

MNKNHVVKSNQVIEASYQLSAVEQRIVLAAISRIPKNQPITDDELYPVSINELRQLGVHEKTAYRDLKEGINRLYERSINLSIDDKSIKMRWVQEVQFLDSQSVIGIRFSKPILPFISNLSREFTKYALSDIAGINSGYGIRIYELLVQYRQIGKREISVENLRTMLELGKKYPLFADFKKRVIDTAIDQINECSPLNVTYEQKKTGRKVTSIIFSFKEKTKSISHQNTDVPKEFYKLTDAQINMFGNQLSRLHELSHLAQQGESYDDLAITIKDMLRDPKQQKQFLPYLKNLGFKL

>GR13_NC_010404.1_ABAYE_RS00110_NCBI WP_000064928.1 RepB family plasmid replication initiator protein

MSNHNEPEELEHLPYCIGNIRHNGVVSTSNRLIRPIELSSNEYKALLYAMAVANYGEKNNQDREITEQTYIYLHKDDLGELLGLDKKNSINVAIDRIYKELSSRVAHFVIEEPVDDNKRKVKKVHSVVPIIRELRWEDDAKNALQIRFTSEVLPYFTRLANGNFTTYQLKDLFALDSVTSMSLYSYFIKNEFKYANQDSYEVELSLENIKALIDIGEKKYDRWVDFRRYVLDKIIEEINERTSLQLEYDTIKKGRPIVGVRFKILNRHATEVVVSNTNDKTQIYLDVNFDDNALVKELGAKFDMTVRSWYIYANDPNYKQFSKWFKTEGCLTESQANVIVNDIMFQMDFAVAGSSMSEFKKEMKYKLKNNPNFVKENRKRLNEIFGKDVI

>GR14_NC_010403.1_ABAYE_RS00085_NCBI WP_001180321.1 hypothetical protein

MQNLDKKKPLLSDSLATGDNKGFASPKGDQHRDRITRFGILKHRSKQQENYLFSLAKIKENYHADVKNDESIRAMKTAQKLNGCGNFLLFKNFYTINQIKLAKFQACSEHLLCPFCAGIRASKAIQKYSERVDQVLSENPRLKPVMITFTVKNGVDLGERFTHLIKSFRTLIERRRDYIKKGRGFNEFCKINGAMYSYENTYNEKTNEWHPHIHMFALLDDWIDQDELSQYWQSITGDSMVVDIRRAKKQKDLGYSGAAAEVCKYALKFGDLSVEKTWEAFKVLKGKRLSGAFGSLWGVKIPESLIDDLPDDSDLPYLEMIYKFVFSKKSYYDLQLTRHVEPTGKDDADELRGEEGRNLLVSMDGRGASDAGRARTGALAPQHGRKKQHWQIPPVTRVRVRKRIRRWDGYLCVLHL

>GR15_CU468233.1_ABSDF_p30018_NCBI CAP02992.1 replication protein

MEIKMMSPSKKELVVKSNQVIEASYQLSSTEQRIVLAAISKISRAEDITDDEIYRVTIDDLKKLGVHEKTAYRDLKDGVNRLYDRSINLAINDESIKMRWIQSIRFLESKSVVGIRFSKEILPFISNLSREFTKYSLSDIAGMSSAYAIRIYELLSQYRSIGKREIPIESLRSMLELGKRYPLSADLKRWVIDTAVDQINEHSPLNVSYQQIKTGRKVTHIQFTFKEKSKNIEHKSEQNDFYKLTDSQINMFGNQLSRLHEVSHLAQQGESYDDLAIKIKDMLRDRIQQKQLIPHLKNLGFKA

>GR16_L77992.1_pAB49_repA_NCBI AAA99423.1 replication protein

MSMNIFYGENPSSASLKSLENSQSLGIKTKSHVMLTPQGFQRVHDYLLQDQSRKLLPKERVSKCRRLRIDKTKTRTVMYNEHREKAHYGNVQICGSIWSCPVCAKQITQKRRNELGKGIESWKTVHNGSVYLLTLTFSHSPDQSLKSNLEGLKRAMKRFYETTRVQAIFKKLSVFHKIKGLEVTYGQNGWHPHHHVLLLAEHHDLRFKDYTSELTELWIKACIKSGLNAPSMRHGLDLRNGSYADQYVSKWGLEDELSKGHVKKGRNGGFTPFDLLNFSIEDNEIYGKKPSKLFQEFAISMKGARQLVWSRGLKKLLGIEEKSDEELAVETDKASITLNRVEDLVFELLCRYQLRHQYLEAIKHDYETGSFGSGLADQLIEQVVNYEIKQMQQVFS

>GR17_CP000522.1_A1S_3461_NCBI ABO13850.1 DNA replication protein

MAKLSLSEVSKRFNVSRSTLYRAIKEGRISRNADGYFDVAEVIRCFGEPSKKHEQNQEIDKPKDDTDLRQLVDFMRKEIDSYKDREKRYLDQIDRFQLLLGHKESEEKMSHDTSVRQTNDTPCDNNHETHKDTVNQEFYSTESPHDTPLTHLNEAPQNISKTHHETKKKRGLFGRVLNAVFDND

>GR18_CU468232.1_ABSDF_p20025_NCBI CAP02966.1 putative replication protein

MLFSYLITRNTKMSKLLVVKANNIIEASYQLSLNEQRLILAAIACIPKGEEVTDNTGYCVTRESFIELGVNPKTASREIREACDRLFNRVITITTEAGTFKTRWVQDIMKYNSDWALANPEFIQEVAGSDPYAEDYILAAIRFSKSVLPFISNLSSNFTQYFLQDIAGVSSGYSVRFYELMMQFKSTGYRKIRLDDLRNMLDLNNKYPLTADLKRWVIDTAIDELNEKSPITIKYKLLKTGRKFTHIELKFKQKLSPKKIESQRDQKTIDMFSNLSDSQIKTYSSVLSKVHSISDLADNKDYSAFAIWIANVLRDPTSVREETAKRIFKTLRTETDFKG

>GR19_GQ861437.1_GQ861437_rep1_NCBI ACX70400.1 putative replicase

MLIVKDNALINASYNLELVEQRLILLAIIEARQNGKGINTNDHLIVHASTYIEHFNVEKHSAYMSLKEACKNLFARQFSYEEINPNGSSTQYTSRWVSKIGYTKKEGTVHIIFAPDVVPLITRLEKHFTSYELEQVAQLQSKYATRLYEILIAWRSTGKVPEISLSEFRAKLGVSDSEYKIISNFKLRVLDVAVSQINKYTDITVTYEQHKKGRTIIGFSFRFKQKQLAKKIESKRDLNTPDFFIKMTDAQRHLFANKMSEMPEMSSYSQGTES

>GR20_NC_012813_pABAVE01_01_NCBI YP_002967453.1 DNA replication protein B

MRELVVKDNALINASYNLDLVEQRLILLAIVEARESGKGINANDPLEVHAEGYINQFGVHRNTAYQALKDACNDLFARQFSYQKINERGNIENYRSRWVSEIGYVDNEAVVKLIFAPAIVPLITRLEEHFTKYELQQVSNLSSAYAVRLYELLIAWRSTGSTPIIEVSDFRQRIGVLDTEYKRMERFKTSVLELAIKQINEHTDITVKYEQHKRGRSISGFSFTFKQKKKDNPPIERDPNTLDLFTKMTDAQRHLFANKLSELPEMGRYSQGTESYPQFAIRIAEMLQDPDRIKELYPYLKKVGYMPSNKKDTVNG

>GR21_KY984046.1_repAci21_NCBI AUO31881.1 Initiator Replication protein

MGELVVKSNDLINASYNLGVVEQRLLLLCIIAARKKDRVLSPSDIFYIHASEYIEQFDVDRSVAYRALAEGIKGIYDSEIKLTSKNSRKKINIRWCWKAEYDEDHATVGVAFTDDVIPLISALEQRFTSYDIDQIAKLTSKYAIRLYELVIAWRSINKTPVFELEDFRNKLGLGVSEYKTMSNFNSNVLNIAIQQINKFTDIKIKVHKHKKGVRIVGFSFELTQRKMKNQNSTKDTFYRLTDSQINMFGNQLSRLHEVAHLAVEGESYEILAAKIKEMLRDPIQQKQFLPHLQNLGFKA

>GR22_KY984047_repAci22_NCBI initiator RepB protein

MKNDLVVKDNALINASYNLDTTEQRLILLAIVQAREVSKDVDANSTLEVHAHHYMKQFNVDKHAAYEGLKNAASNLFERKFSYKGIHEGTQQEKIVKSRWVSKIAYVDSAGIVELTFAPDVIPLITQLEKSFTAYELKQISSLTSKYAIRLYELLIQWRSVGKTPMFDIDDFRFKLGLAEGEYAKMANFKVRVLDIALNQINELTDITASYEQHKVGRTISGFSFSFKPKQHVDAITHNKPKKLTDKQIQFFANKLAHHDPFASKKAAVGESYADLEKRLLIELQDVEFVRKYASVLKELGLEV

>GR23_KY984047_repAci23_NCBI plasmid replication protein Aci23

MSELIVKDNALIQASYTLDTVEQRLILLAIAEARETGHGINENSLLQVHASSYINTFNVEKHTAYTVLRDASKSLFDRYVTYHDINPKTDKDRSFHCRWVDKIGYEPQSGIVFLRFTQDIVPLITRLEENFTKYELQQVSRLTSSYAIRLYELLIQWGSRGKTPTFDLHVFRNRLGVEDGQYKTMCNFKQFVLDFALKQINQFTDIIAKYEQHKSGRKITGFSFTFKFKNNKNVKEKLVEKTEFYKLTESQLDLFAKKLAHLPELGHLADEGMSYEEFYSKLKSILKDPEQQKKLVPYFEKAGLNPK

>GR24_NZ_AFCZ02000003.1_ACIN5032_RS22220_NCBI WP_000818857.1 RepB family plasmid replication initiator protein

MAELIRNSDVYKANALINASYALDTAEQRIILLAILVSRNKNADLTAETIIEIPASLYAQKFNTTVSAAYKTLKEAEDTLFERRFSYTTMRNGKIEVVRSRWVSRVSYVKDDALLTITLAPDVIPLVTKLEGTFTKYAIDNLRDVTSKYGIRLYELVASWKNSDIRKTPVYDFEDFRAKMGLLPHEYRDKKNPESTDMTNFNKRVLKPAIDQINSFTDLFITEKKIKTGRNITGIYFEVSLKTDNFIEGEAKEIHDSKPSSDSAKKTGTPLENIRLPKVSTQEFLGSDLSEEDLNKENPLKEFIVESGVYKSAIEKPVEEKDEFELNGIKRLYEALLKLDEGVTKEYVREYAQIKGVTLQHALIELYNSKRPA

>GR25_NC_017848.1_ABTJ_RS19085_NCBI WP_000633173.1 plasmid replicase

MITSNIFATDNKSILISRLYENLPRKPYCTNDFFGLRIREKKSAISHSHIQFNHPSFKRYIVIDADYAGAATAWRYEFAENIPVPNLIVTNPENSHCHFYYELSAPVSFTDSSSKKAQEFYNAVSKKLTEVLRGDTNYTGLIAKNPAHEKWIVEAPRIETYSLHELVEHLELHPHEYRSIPGQNSKQEQVQCINGRNDHLFHTVRVKAYVDVRDFRSKTYPQWEEHVRQLLVDHNLELNNPLPYSEIKATAKSIAKYCWKKDGYCYQEFCDRQISKAKKGGQAKADKYIELRKKAVALLRKGKNKRLIAQLLKVSYRSVLRWLYNVKLAAAIMHLRDLKNMCDNAQNQILAAFVASLAVLFLDEFIYDFTQDDILTINLTFNIKMLI

>GR26_NZ_CP015365.1_Aba3207_RS19230_NCBI WP_063558588.1 RepB family plasmid replication initiator protein

MADLVVKSNKLVQALQTLTLSETRLLQLAIVDARETGQGLSAEEPLELNASRYATAFNVSPDAAYLALVEAEDSLFKRQFTITNEDGTLTKSRWIQDANYRKGEGRILVTLTRVVIEHVTKIDGFEQYFTSYHLKKTSDFKSVYAVRLYELLMQWKSVGKTPIYELNKFRSQLGIGVNEYDRMEAFKRRVLDIAIKQINELSDITVKYEQHKKGRAISGFSFAFKQKKTNQPIADKRDPNTLDLFSKMTDAQRHMFANKLSELPEMGKYSQGTESYQQFAVRIAEMLQDPEKIKELSPYLKKVGYMPSNKKDTVNG

>GR27_NZ_AYFH01000057.1_P676_RS10150_NCBI WP_004282236.1 RepB family plasmid replication initiator protein

MDENKKTYPPSWVVMQNNIQECFKSMNIDEKRILILASPIARTTQATEKDPIMITAEKFAEECGIKTHSAYTQLEVASRNLIKRSFSYNNERGKRVLSNWVIDCIYEDGGIAIRFPEIVLLMLTEFDKLNPYTKYKKDIVLSLKKDYSFDFYHLAKKHQAMGKFEMSLERIRTEFGLPESYHDLSNLKKRVINPSLDEITANTDIALTYENVKKGRSVVGFKFTVREKPKPRLIASEREQETLDIFRSLSDGQINTYSSILSKVGSISDLAGAKDYQAFAIWIANILRDPKSVREETAKRIFKALRTETDFKG

>GR28_NZ_AFDB02000003.1_ACINNAV81_RS23405_NCBI WP_000185726.1 replication protein RepB

MTNTNKLVVKDNALIDASFNLSLIEQRIMLLAIVEARESNSLSPDTPIEVSVSDYIHQFKVDSNNAYALLKDASKTLKRREFSYLDRYKGIEALSTANWVNKVTYVDKSGLIVLYLSHEVISLISKLSEQFTKYYIEQVSEFKSKYSIRLYELIIKWLSVAKTEKYSINDLRSKLGLGVEEYSTMTNFKSNVLDKAINEINKHTDIIVDYQQFKKGRVITDIQFFIKSKARPSKQTNTTKQSFYQMNDAQINLFGNQLSRLHELSHLANQGESYDELAIKIKDMLRDPIQQKQLLPHLKNLGFKA

>GR29_CP023021_Aba9201_20650_NCBI WP_000360254.1 RepB family plasmid replication initiator protein

MDKNQIVKSNQVIEASYQLSAVEQRIVLAAISRIPKSQPITDDELYPVSVNELQLLGVHEKTAYRDLKEGINRLYERSINLSIDDKSIKMRWVQEIQFLDSQSIIGIRFSKPILPFISNLSREFTKYALSDIAGINSGYGIRIYELLVQYRQIGKREISVENLRSMLELGKKYPLFADFKKRVIDTAVDQINECSPLSVSYEQKKTGRKVTHIQFSFKEKSKSINHLNEPEKFYKLTDAQVHMFGNQLSRLHELSHLATQGESYDDLAATIKEMLRDPIQQKQFIPHLRNLGFKG

>GR30_NZ_AYOI01000002.1_V427_RS00680_NCBI WP_000095317.1 RepB family plasmid replication initiator protein

MSSIVKSNPKVKKHNNLTQAHFFNVSVIAYRLILLAGTDKFLENMLKSGENTYIRITAHDYHNLYGSSSDMSGSYKAIKDAPDDLLNAKLKYKRLKTESDPGRWVGGINWVQDARYNDELKCVEILFSTTVLPLLANVRKSFTYYNLRHIGRLSSMHSIRMYELMMMWRKSGKTPDLTVSYMKNFLGVPDNEYSDPKELKFFTAQVIKKSVKEVTSKTNIEMDFEVVRGEKRATIGYSFSHKLKALPEGEQPEQEELEDDNEGGGDPSKLLPNNDDDPELPF

>GR31_NZ_CP012956.1_AN415_RS19570_NCBI WP_000140303.1 RepB family plasmid replication initiator protein

MTESEPLQKESNYIPYCIGNIRHNGVVSTSNRLIRPIELSANEYKALLYAMAVANYSEKNRVNGEITEQTYIYLYKDDLADLLGLNKRNSINVAIDRIYKELSSRVAHFIIEEPADDGKRKTKKVHSVVPIIRELRWEDDSKNAIQIRFTNEVLPYFTQLAGGNFTTYQLKHLFALDSVASMSLYTYFIKNEFKYANQKSYEVPLLLENLKAVIDINETKYDRWVDFRRYVLDKIVAEINENTDLQLEYETVKKGRPIVGVNFKLHHRIADKAPDEIAVIEKIYLDVPFEDNAFVKELGAKFDTNIRSWYIFNNHENYQQFKKWFKKVGCLTDSQANIVINDTLFQMDFAEIGMGLNDFKRNMKHKLKNNPEFVQSIRERLNDIFGKELI

>GR32_NZ_CP013925.1_KBNAB1_RS20020_NCBI WP_059273206.1 plasmid replicase

MFGMTNNNLALASNDSLTIVLKRFYNNLPDKPYHSNGFDVEGLKINRKIEAIKKKYIQFNHPKWKKYILIDIDRPGAVTDWLYESPHLPAPNLIIENRKNGHAHFVYELIDAVSFTERSSLKAKNYYNAVEKALTSELGGDERYNGVVGKNPYSEEWRTSTYRTEAYHLKDLASKLELTTMGLTPIEMPQKAQNDECAINGRNDEVFHSVRHLAYKDIRDFKNNADLLFNHWFDHVLKLVQEKNSFFINPMDYKECTHIAKSISEYCWRNHEECYKQFVERQRNKGSKGGTSRSAKYEEARRMTKQLFRQGVSLKQIAEKLNISYRTAVRYTKGLLRIKLLSFNDINNLRKSALADKKARSEAKCIKSERSESINNAFNWCDSSQNQVLAARHTHPAPFGVFFKKLLSFTFKNLKFERLKGGAIFYYYGKIP

>GR33_NZ_CP012005.1_ACX60_RS18650_NCBI WP_000743064.1 replication protein C

MKKPKHDLTHVRHDPAHCLAPGLFRSLKRGDRKRCKLDVTYTFGEDESMRFVGFEPLGADDMRLLQGIVALGGPNGILLTPEPTSETGRQLRLFLEPRFEAIEQDGLVVRESLTKLLSETGMTDSGDNIKALKASLLRMSNVTILVTKGRRQAAFHLMSHAFDETDGRLWVALNPRIAEAILGHRPYARIDMAEVRVLQTDPARLMHQRLCGWIDPGKSGRVELDTLCGYVWPDEANAEAMKKRRQTARKALAELAAVGWVVNEYAKGKWEIKRPGPTATAPVYRRNVPLLPS
